# Trophic Interactions Are Key to Understanding the Effects of Global Change on the Distribution and Functional Role of the Brown Bear

**DOI:** 10.1111/gcb.70252

**Published:** 2025-06-04

**Authors:** Pablo M. Lucas, Wilfried Thuiller, Lauren Talluto, Ester Polaina, Jörg Albrecht, Nuria Selva, Marta De Barba, Vincenzo Penteriani, Maya Guéguen, Niko Balkenhol, Trishna Dutta, Ancuta Fedorca, Shane C. Frank, Andreas Zedrosser, Ivan Afonso‐Jordana, Hüseyin Ambarlı, Fernando Ballesteros, Andriy‐Taras Bashta, Cemal Can Bilgin, Neda Bogdanović, Edgars Bojārs, Katarzyna Bojarska, Natalia Bragalanti, Henrik Brøseth, Mark W. Chynoweth, Duško Ćirović, Paolo Ciucci, Andrea Corradini, Daniele De Angelis, Miguel de Gabriel Hernando, Csaba Domokos, Aleksander Dutsov, Alper Ertürk, Stefano Filacorda, Lorenzo Frangini, Claudio Groff, Samuli Heikkinen, Bledi Hoxha, Djuro Huber, Otso Huitu, Georgeta Ionescu, Ovidiu Ionescu, Klemen Jerina, Ramon Jurj, Alexandros A. Karamanlidis, Jonas Kindberg, Ilpo Kojola, José Vicente López‐Bao, Peep Männil, Dime Melovski, Yorgos Mertzanis, Paolo Molinari, Anja Molinari‐Jobin, Andrea Mustoni, Javier Naves, Sergey Ogurtsov, Deniz Özüt, Santiago Palazón, Luca Pedrotti, Aleksandar Perović, Vladimir N. Piminov, Ioan‐Mihai Pop, Marius Popa, Maria Psaralexi, Pierre‐Yves Quenette, Georg Rauer, Slaven Reljic, Eloy Revilla, Urmas Saarma, Alexander P. Saveljev, Ali Onur Sayar, Çagan H. Şekercioğlu, Agnieszka Sergiel, George Sîrbu, Tomaž Skrbinšek, Michaela Skuban, Anil Soyumert, Aleksandar Stojanov, Egle Tammeleht, Konstantin Tirronen, Aleksandër Trajçe, Igor Trbojević, Tijana Trbojević, Filip Zięba, Diana Zlatanova, Tomasz Zwijacz‐Kozica, Laura J. Pollock

**Affiliations:** ^1^ Institute of Nature Conservation, Polish Academy of Sciences Kraków Poland; ^2^ Department of Biology and Biotechnologies “Charles Darwin” Sapienza Università di Roma Rome Italy; ^3^ Departamento de Biología Vegetal y Ecología Universidad de Sevilla Sevilla Spain; ^4^ Univ. Grenoble Alpes, Univ. Savoie Mont Blanc, CNRS, LECA Grenoble France; ^5^ Department of Ecology University of Innsbruck Innsbruck Austria; ^6^ Department of Ecology Swedish University of Agricultural Sciences Uppsala Sweden; ^7^ Senckenberg Biodiversity and Climate Research Centre (SBiK‐F) Frankfurt am Main Germany; ^8^ Estación Biológica de Doñana CSIC Sevilla Spain; ^9^ Departamento de Ciencias Integradas, Facultad de Ciencias Experimentales, Centro de Estudios Avanzados en Física, Matemáticas y Computación Universidad de Huelva Huelva Spain; ^10^ Biotechnical Faculty, Department of Biology, University of Ljubljana Ljubljana Slovenia; ^11^ DivjaLabs Ltd. Ljubljana Slovenia; ^12^ Department of Evolutionary Ecology National Museum of Natural Sciences (MNCN‐CSIC) Madrid Spain; ^13^ Wildlife Sciences University of Goettingen Goettingen Germany; ^14^ European Forest Institute Bonn Germany; ^15^ Department of Wildlife National Institute for Research and Development in Forestry “Marin Drăcea” Brasov Romania; ^16^ Department of Silviculture Transilvania University of Brasov Brasov Romania; ^17^ Faculty of Technology, Natural Sciences and Maritime Sciences, Department of Natural Sciences and Environmental Health, University of South‐Eastern Norway Bø i Telemark Norway; ^18^ Departament de Territòri, Paisatge e Gestion Ambientau Conselh Generau d'Aran Vielha Spain; ^19^ Department of Wildlife Ecology and Management, Faculty of Forestry No: 130 Düzce University Düzce Türkiye; ^20^ Brown Bear Foundation Santander Spain; ^21^ Institute of Ecology of the Carpathians NAS Ukraine Lviv Ukraine; ^22^ National Park “Skolivski Beskydy” Skole Lviv Region Ukraine; ^23^ Department of Biology Middle East Technical University Ankara Türkiye; ^24^ Faculty of Biology University of Belgrade Belgrade Serbia; ^25^ Estonian University of Life Sciences Tartu Estonia; ^26^ Servizio Foreste e Fauna Provincia Autonoma di Trento Trento Italy; ^27^ Norwegian Institute for Nature Research (NINA) Trondheim Norway; ^28^ Department of Wildland Resources Utah State University Vernal Utah USA; ^29^ Animal Ecology Unit, Research and Innovation Centre Fondazione Edmund Mach San Michele all'Adige Italy; ^30^ Department of Conservation Biology Estación Biológica de Doñana (EBD‐CSIC) Sevilla Spain; ^31^ Milvus Group Bird and Nature Protection Association Tîrgu Mureș Romania; ^32^ Wildlife Society Sofia Bulgaria; ^33^ Hunting and Wildlife Program Kastamonu University Araç Kastamonu Türkiye; ^34^ Department of Agri‐Food, Environmental and Animal Sciences University of Udine Udine Italy; ^35^ Natural Resources Institute Finland Oulu Finland; ^36^ Protection and Preservation of Natural Environment in Albania Tirana Albania; ^37^ Faculty of Veterinary Medicine University of Zagreb Zagreb Croatia; ^38^ Natural Resources Institute Finland Helsinki Finland; ^39^ Department for Forestry, Biotechnical Faculty University of Ljubljana Ljubljana Slovenia; ^40^ ARCTUROS, Civil Society for the Protection and Management of Wildlife and the Natural Environment Florina Greece; ^41^ Faculty of Environmental Sciences and Natural Resource Management Norwegian University of Life Sciences Ås Norway; ^42^ Norwegian Institute for Nature Research Trondheim Norway; ^43^ Department of Wildlife, Fish and Environmental Studies, Faculty of Forest Sciences Swedish University of Agricultural Sciences Umeå Sweden; ^44^ Biodiversity Research Institute (CSIC—Oviedo University—Principality of Asturias), Oviedo University Mieres Spain; ^45^ Estonian Environment Agency Tallinn Estonia; ^46^ Macedonian Ecological Society Skopje North Macedonia; ^47^ “Callisto” Wildlife and Nature Conservation Society Thessaloniki Greece; ^48^ Progetto Lince Italia Tarvisio Italy; ^49^ Adamello‐Brenta Natural Park Strembo Italy; ^50^ Central Forest State Nature Biosphere Reserve Zapovedniy Tver Region Russian Federation; ^51^ Nature Conservation Centre Ankara Türkiye; ^52^ Fauna and Flora Service Generalitat of Catalonia Barcelona Spain; ^53^ Stelvio National Park Bormio Italy; ^54^ Servizio Faunistico Provincia Autonoma di Trento Trento Italy; ^55^ Centre for Protection and Research of Birds of Montenegro—CZIP Podgorica Montenegro; ^56^ Department of Game Resources Russian Research Institute of Game Management and Fur Farming Kirov Russian Federation; ^57^ Research and Development Institute for Wildlife and Mountain Resource Miercurea Ciuc Romania; ^58^ The Association for the Conservation of Biological Diversity (ACDB) Focsani Romania; ^59^ Department of Ecology, School of Biology Aristotle University Thessaloniki Greece; ^60^ Research and Scientific Support Direction French Biodiversity Agency Villeneuve de Rivière France; ^61^ WWF Austria Vienna Austria; ^62^ Institute of Wildlife Biology and Game Management University of Natural Resources and Life Sciences Vienna Austria; ^63^ Oikon Ltd.—Institute of Applied Ecology Zagreb Croatia; ^64^ Department of Zoology, Institute of Ecology and Earth Sciences University of Tartu Tartu Estonia; ^65^ Department of Game and Wildlife Cankiri Karatekin University Çankırı Merkez/Çankırı Türkiye; ^66^ School of Biological Sciences University of Utah Salt Lake City USA; ^67^ Department of Molecular Biology and Genetics Koç University İstanbul Türkiye; ^68^ Carpathian Widlife Society Zvolen Slovakia; ^69^ Institute of Biology Karelian Research Centre, Russian Academy of Sciences Petrozavodsk Russian Federation; ^70^ Faculty of Natural Science and Mathematics University of Banja Luka Banja Luka Bosnia and Herzegovina; ^71^ Faculty of Ecology Independent University of Banja Luka Banja Luka Bosnia and Herzegovina; ^72^ Ecology and Research Association Banja Luka Bosnia and Herzegovina; ^73^ Tatra National Park Zakopane Poland; ^74^ Faculty of Zoology Sofia University “St. Kliment Ohridski” Sofia Bulgaria; ^75^ Department of Biology McGill University Montréal Canada

**Keywords:** climate change, community, ecosystem, food web, habitat, human impact, land use, predator–prey, species distribution model, *Ursus arctos*

## Abstract

Biotic interactions are expected to influence species' responses to global changes, but they are rarely considered across broad spatial extents. Abiotic factors are thought to operate at larger spatial scales, while biotic factors, such as species interactions, are considered more important at local scales within communities, in part because of the knowledge gap on species interactions at large spatial scales (i.e., the Eltonian shortfall). We assessed, at a continental scale, (i) the importance of biotic interactions, through food webs, on species distributions, and (ii) how biotic interactions under scenarios of climate and land‐use change may affect the distribution of the brown bear ( *Ursus arctos* ). We built a highly detailed, spatially dynamic, and empirically sampled food web based on the energy contribution of 276 brown bear food species from different taxa (plants, vertebrates, and invertebrates) and their ensemble habitat models at high resolution across Europe. Then, combining energy contribution and predicted habitat of food species, we modelled energy contribution across space and included these layers within Bayesian‐based models of the brown bear distribution in Europe. The inclusion of biotic interactions considerably improved our understanding of brown bear distribution at large (continental) scales compared with Bayesian models including only abiotic factors (climate and land use). Predicted future range shifts, which included changes in the distribution of food species, varied greatly when considering various scenarios of change in biotic factors, providing a warning that future indirect climate and land‐use change are likely to have strong but highly uncertain impacts on species biogeography. Our study confirmed that advancing our understanding of ecological networks of species interactions will improve future projections of biodiversity change, especially for modelling species distributions and their functional role under climate and land‐use change scenarios, which is key for effective conservation of biodiversity and ecosystem services.

## Graphical abstract

This study shows that what animals eat—and where their food lives—can strongly influence where the animals themselves are found, even across entire continents. By modeling the diet and habitat of the brown bear across Europe, the research reveals that including food web relationships improves predictions of where bears live now and where they might move in the future as climate and land‐use change. These findings highlight the importance of considering species interactions, not just climate, when planning for biodiversity conservation.

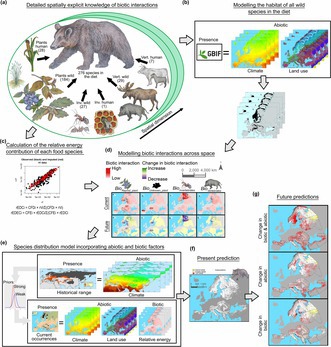

## 1 Introduction

In the current biodiversity crisis (Pereira et al. 2024), understanding how the distribution of species will be impacted by global changes, such as climate and land‐use changes (Chen et al. 2011; IPCC 2014), is critical for conserving biodiversity and securing associated ecosystem services (Urban et al. 2016) including human food systems (O'Neill et al. 2017). One of the major outstanding challenges is to capture the complexity of biological responses when making predictions about how species will respond to global changes, as their distributions are shaped by a complex set of abiotic (fundamental niche) and biotic factors (realized niche) (Hutchinson 1957; Nogues‐Bravo 2009; Payne et al. 2024).

Species distribution shifts in response to global changes are highly variable (Lucas, González–Suárez, et al. 2016; Pacifici et al. 2020; Le Luherne et al. 2024), and species' ecological traits such as the climate niche, habitat specificity, and mobility determine the redistribution of species (Pacifici et al. 2017; Pacifici et al. 2020; Carroll et al. 2024). Furthermore, species within an ecological community can shift their habitat at different rates and directions, altering the original overlap of species, with congruous, convergent, divergent, or contracted shifts (Durant et al. 2007; Carroll et al. 2024). These differences in the redistribution of species within the same community can lead to a change in the spatial overlap between prey and predator, strengthening or weakening their interactions (Durant et al. 2007; Carroll et al. 2024). Predators will respond differently to prey redistribution depending on their ecological traits (e.g., trophic position and diet breadth) and environmental factors (e.g., availability of alternative prey) (Carroll et al. 2024). They can respond by prey‐switching (diet generalist species), or the predator's fitness might be affected—change of predator realized niche—causing bottom‐up effects on the predator's population and, in the worst scenario, its local extinction (diet specialist species or absence of alternative preys) (Ferreras et al. 2011; Carroll et al. 2024). At the community level, this can lead to changes in interaction strengths and food web topologies. In summary, communities and biotic interactions are dynamic over time and are influenced by global changes (Blois et al. 2013; Bartley et al. 2019). However, most existing biogeographical studies that aim to predict species responses to global changes rely primarily on abiotic factors, especially climate. This is, in part, a result of the perceived differences in scale, with abiotic factors thought to operate at larger spatial scales (Willis and Whittaker 2002), and biotic factors, such as species interactions, being considered more important at local scales within communities (Willis and Whittaker 2002). Such reliance on abiotic factors when explaining large‐scale species distributions has also resulted from extremely sparse data on species interactions. This knowledge gap on species interactions, also termed the Eltonian shortfall (understood as the lack of knowledge on intra‐ and interspecific interactions, but also as the physiological tolerances of species, and the effects of species on ecosystems), severely limits our understanding of large‐scale biodiversity patterns (Hortal et al. 2015).

**FIGURE 1:**
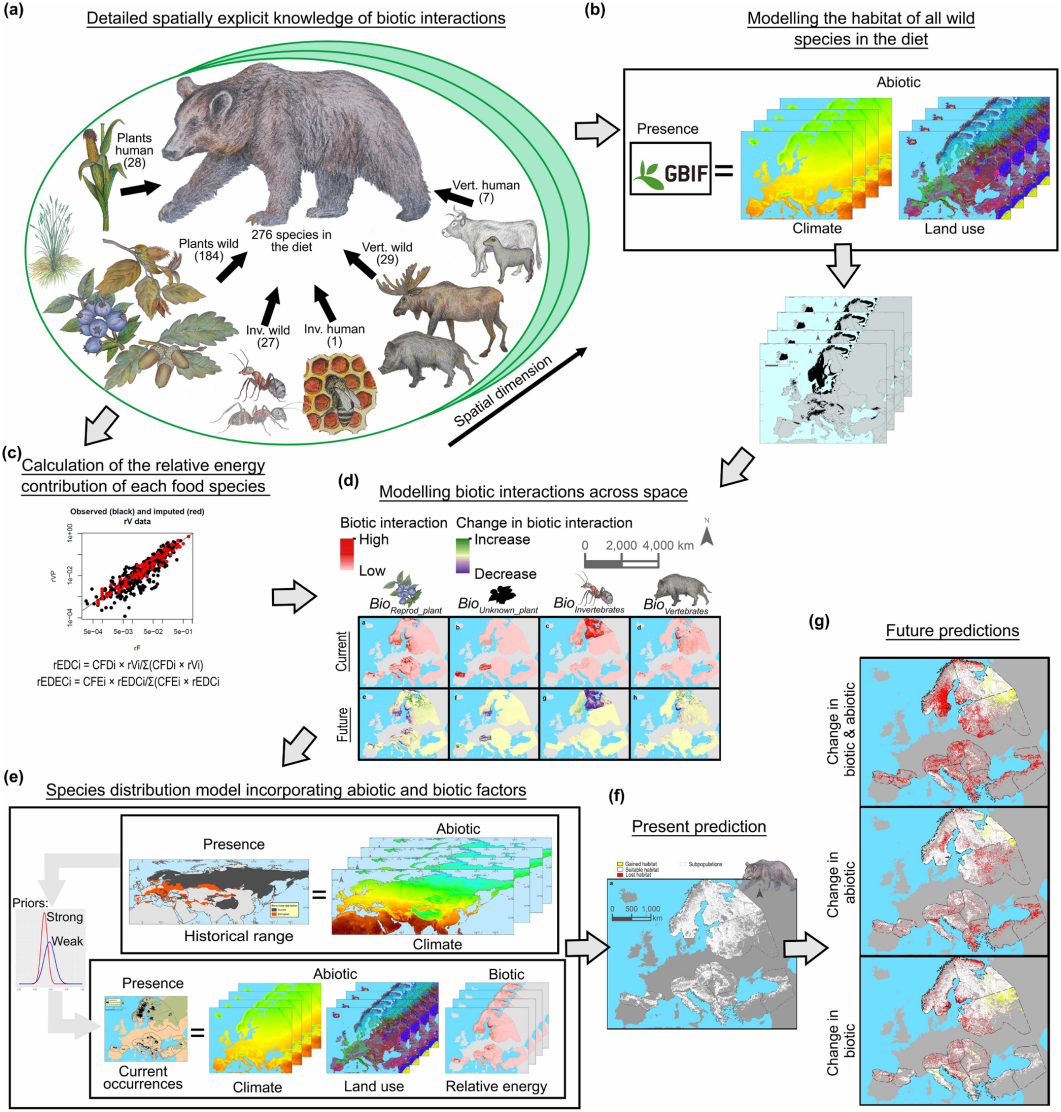
Diagram showing our model system to assess the importance of biotic interactions for understanding the consequences of global change for biodiversity. (a) Construction of a database with detailed explicit knowledge of biotic interactions (in our model system, brown bear food species in Europe) based on a literature review which accounts for the spatial variability of interactions. (b) Fitting ensemble species distribution models (SDMs) for wild food species and calculation of habitat suitability for the current and three future (2040) shared socioeconomic pathways, which were included using predictions for climate (Karger et al. 2017) and land‐use changes (Schipper et al. 2020). (c) Calculation of the relative energy contribution of each food species in different subpopulations/space. (d) Calculation of quantitative and binary proxies of biotic interactions across the space and predictions of the spatial biotic interactions for each scenario. (e) Fit of a species distribution model for the brown bear combining historical and current data and incorporating abiotic factors, which refer to the effects of global changes that directly impact the brown bear, including temperature changes (i.e., affecting hibernation and reproduction) and land‐use changes (i.e., decreasing suitable habitat), and biotic factors, which refer to the effects of global changes through biotic interactions such as changes in the availability of other species as food sources. (f, g) Current and future predictions for brown bear distribution considering both abiotic and biotic factors.

Despite these limitations, studies are beginning to incorporate species interactions for understanding species distribution shifts due to global changes (Guisan and Thuiller 2005; Penteriani et al. 2019; Bas et al. 2025; Hao et al. 2025). These studies have demonstrated that adding information on other species significantly improves the understanding of distribution changes (Wisz et al. 2013; Pollock et al. 2014) and allows assessing changes in the ecosystem structure and functioning (Bas et al. 2025; Hao et al. 2025), suggesting that species interactions are a valuable component in understanding the effects of global changes on biodiversity (Carroll et al. 2024; Hao et al. 2025). However, the approaches used to include species interactions usually face three important limitations: (1) They typically only include spatial co‐occurrence as a surrogate for species interactions (Meier et al. 2010; Belmaker et al. 2015), (2) they usually use a binary measure for interaction, for example, presence/absence of an interaction between *Species*_*A*_ and *Species*_*B*_, and (3) it is assumed there is no spatial variation in the interaction, for example, interaction between *Species*_*A*_ and *Species*_*B*_ is considered constant in all ecosystems (Banašek‐Richter et al. 2009). These assumptions could be improved with: (1) real data on ecological interactions, for example studying the trophic interactions among species; (2) describing the interactions among species with quantitative measures, for example, measuring the relative energy obtained from food/consumed species; and (3) incorporating the spatial variability of those interactions among different ecosystems (Dormann et al. 2018; Galiana et al. 2018; Blanchet et al. 2020), for example, measuring different values of the relative energy from food/consumed species across geographic space. Therefore, to advance our understanding of species distributions, it is necessary to adopt a cross‐scale approach merging community ecology represented by local‐scale biotic interactions over broader scales (Wisz et al. 2013) with abiotic factors (Lavergne et al. 2010; Boulangeat et al. 2012) based on detailed community ecology knowledge (Figure 1) (Dudenhöffer et al. 2022; Carroll et al. 2024).

Here, we assess whether considering detailed diet data at large spatial scales helps to understand future consequences of global change for the redistribution of species and their role in the ecosystem structure and functioning. Specifically, we tested (i) how biotic interactions, based on diet, change over space, (ii) whether species' geographic distributions are better estimated by quantitative (continuous) or binary proxies of biotic interactions, (iii) whether species' geographic distributions are better explained when combining biotic (e.g., prey availability) and abiotic factors (e.g., climate and land use), and (iv) whether or not future range shifts differ when considering biotic interactions in addition to abiotic factors. Trophic interactions are among the most important biotic factors determining species distributions and are fundamental to ecosystems (Braga et al. 2019). We used as a model system a top predator and generalist omnivore species with a strong impact on ecosystems (Penteriani and Melletti 2020) and with several of its subpopulations at extinction risk (McLellan et al. 2017), the brown bear *Ursus arctos* in Europe and Türkiye (formerly known as Turkey). This model system has a broader applicability to understanding ecosystems in general, higher‐level predators in particular, and could be extended to other species.

## 2 Material and Methods

### 2.1 Biotic Interactions

**FIGURE 2:**
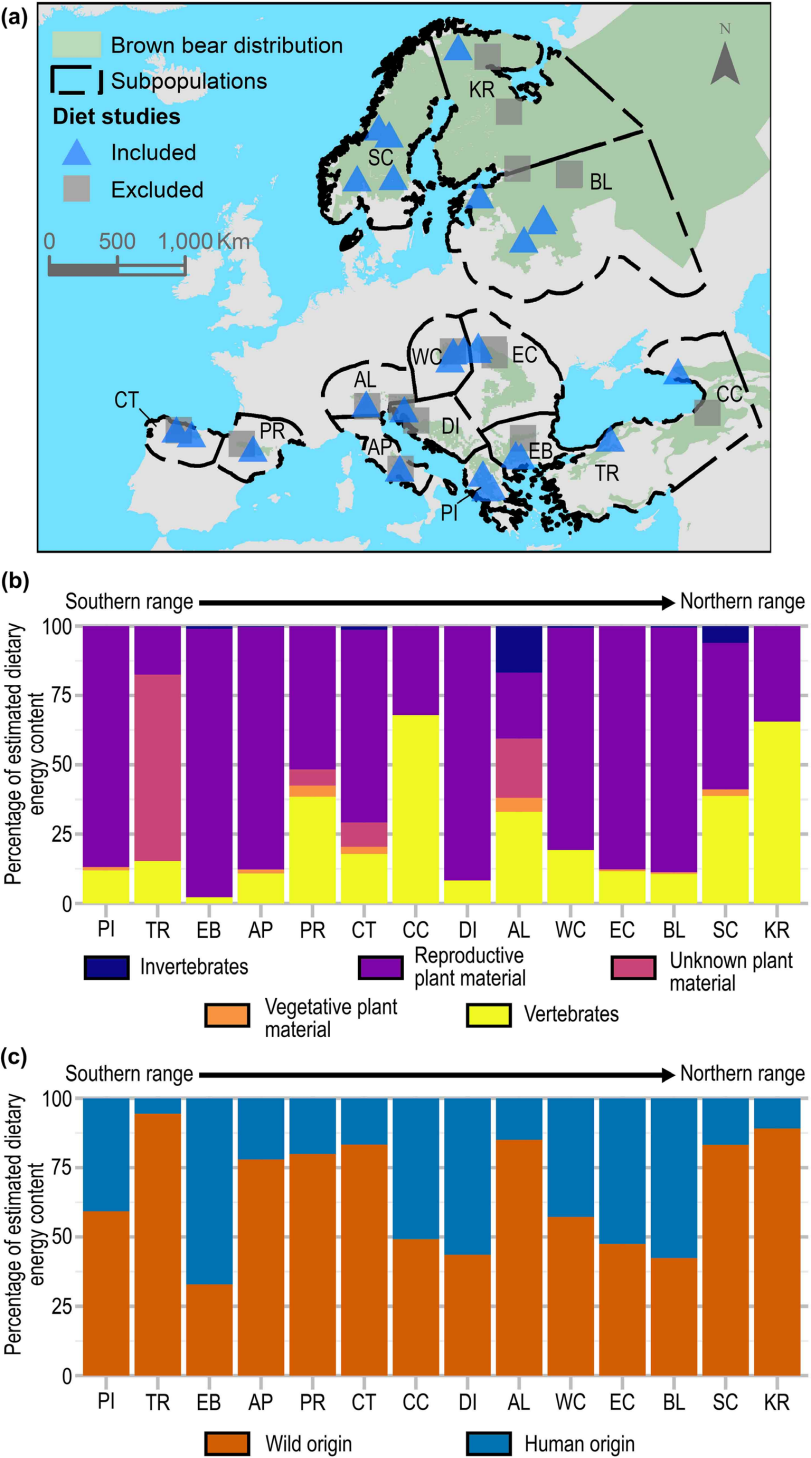
Brown bear diet in Europe. (a) Map showing the 14 brown bear subpopulations considered: Pindos (PI), Türkiye (TR), East Balkan (EB), Apennine (AP), Pyrenees (PR), Cantabrian (CT), Caucasian (CC), Dinaric (DI), Alpine (AL), Western Carpathian (WC), Eastern Carpathian (EC), Baltic (BL), Scandinavian (SC), and Karelian (KR). We also mark the location of all the studies of brown bear diet reviewed, indicating if they were ultimately included (*n* = 31) or not (*n* = 16) for the calculation of biotic interactions at species level and for the associations between diet and environmental variables; note that not all studies are visible due to the overlapping of locations. (b) Relative estimated dietary energy content (rEDEC, a proxy for the relative importance of each item in the diet) identified at the species level for each food category, for each of the 14 brown bear subpopulations in Europe. (c) Proportion of the rEDEC for wild species and those of human origin.

To obtain knowledge of biotic interactions, we reviewed studies of brown bear diet in Europe and Türkiye, constructed a unique, highly detailed, spatially explicit database of trophic interactions (*Trophic Database*; Figures 1a and 2; Tables S1–S4 and Figures S1; see Figure S2 for a detailed diagram of methods), and calculated the relative energy contribution of each food item (Figure 1c).

### 2.2 Review of Brown Bear Diet Studies

We reviewed 47 studies of brown bear diet by searching in SCI Journals, master's and PhD theses, and gray literature (research that is either unpublished or has been published in noncommercial form, for example, technical reports, conference proceedings; Tables S1 and S2 and Figure S1). For each study, we recorded three types of information: study area location; the type of samples, for example, brown bear scat or the stomachs of dead individuals; and the number of samples. Additionally, within each study and for each food item we recorded two parameters: (1) the relative frequency of occurrence (*rF*), calculated as the number of occurrences of food item *i* divided by the total number of occurrences of all food items, that is, *rF*_*i*_ = *f*_*i*_/∑*f*_*i*_; and (2) the relative volume (*rV*), calculated as the volume of food item *i* divided by the total volume of all food items, that is, *rV*_*i*_ = *v*_*i*_/∑*v*_*i*_.

### 2.3 Calculating Energy Available From Food Items

Because not all studies reported estimates of *rV*, we used the strong relationship between *rF* and *rV* (*r* = 0.86, 0.81–0.90 95% CI from bootstrapped correlation coefficients; Figures S3–S5) to impute *rV* for those studies with missing data. Then, we used *rV* to calculate. The relative estimated dietary energy content (rEDEC) (Hewitt and Robbins 1996) of each food item *i* in each study: rEDEC_*i*_ = CF_*Ei*_ × rEDC_*i*_/∑(CF_*Ei*_ × rEDC_*i*_) (Appendix S1, Tables S2–S4).

### 2.4 Calculating Associations Between Diet and Environmental Variables

To address question (i), namely whether biotic interactions change over space and if they are explained by environmental factors, we used averaged linear models predicting the relative energy contribution of different food categories (i.e., reproductive plants, vegetative plants, unknown plants, invertebrates, and vertebrates; Table S5) and the diet diversity using these food categories as a function of climate (Karger et al. 2017) and land use (Schipper et al. 2020) variables. For consistency with climate and land‐use data, we selected 31 studies conducted between 1989 and 2018 which had sufficient taxonomic resolution (genus and/or species; Table S1) to calculate the association between latitude, land cover, and climate variables and the rEDEC in each diet category. We first calculated the value of climate (Karger et al. 2017) and land use (Schipper et al. 2020) variables (the 19 bioclimatic variables and 8 variables describing the percentage of land use by cell; for a full description see of variables see Tables S6 and S7) within a buffer area of 18 km (an area of 1018 km^2^) around the site locations of the selected studies calculated using ArcMap 10.5 (Esri Inc. 2016). We eliminated highly correlated variables using the variance inflation function (VIF) (Dormann et al. 2013) in the R package usdm (Naimi 2017). Then, we calculated all possible linear models explaining the percentage of each diet category using all possible combinations of the remaining uncorrelated variables as predictors, using the package MuMIn (Barton 2009) in R. Using the subset of best models (delta Akaike information criterion, corrected, AICc < 3) we calculated an average model using the subset option. We also calculated models explaining diet diversity (among the diet categories) as a function of land use and climate variables in the buffer areas. We first calculated three indexes of diversity for the diet categories (Simpson, Shannon, and Inverse Simpson) (Fisher et al. 1943; Hurlbert 1971) using the R package vegan (Oksanen et al. 2013). We fitted linear models explaining each diversity index as a function of the uncorrelated variables previously calculated. We calculated, for each index, all possible models using all possible combinations from the uncorrelated variables, and using the subset of best models (delta AICc < 3) we calculated an average model using the subset option.

### 2.5 Calculation of a Representative Diet for Each Subpopulation

We used the selected 31 studies of brown bear diet (Table S1) to calculate the representative diet for each subpopulation (Appendix S1).

Similarly to Banašek‐Richter et al. (2004), where consumed biomass was used as a quantitative descriptor for the flow of energy in the food system, we used the rEDEC previously calculated, which provides a more realistic version of the flow of energy in the brown bear food web system. From the diet studies within each subpopulation, Subp, we calculated for each food species (*S*), the rEDEC_SubpS_, and assumed it to be a representative rEDEC in that subpopulation:

$${\text{rEDEC}}_{\text{SubpS}}=\sum_{i=1}^{n}\frac{\left(Z_{i}\times {\text{rEDEC}}_{\mathrm{Si}}\right)}{\sum_{i=1}^{n}Z_{i}}$$

where rEDEC_SubpS_ is the representative rEDEC in the subpopulation Subp for food species *S*, *i* is each diet study within the subpopulation Subp (Figure S1 and Table S1), *n* is the number of diet studies in the subpopulation *Subp* (Table S1), *Z* is the number of sampling units (*n* scats, or *n* of stomachs analysed) in each study (we use this term to give more importance to studies with more data; Table S1), and rEDEC_*Si*_ is the rEDEC of food species *S* in diet study *i* (Table S4).

### 2.6 Calculation of Habitat Suitability for Each Wild Food Species

We fitted ensemble species distribution models (SDMs) at 1 km^2^ resolution for each wild food species in the *Trophic Database* (Tables S5 and S8), using Global Biodiversity Information Facility (GBIF) occurrences (GBIF 2018, 2023). GBIF data are spatially biased, and this may produce wrong modelling of species distribution if it is not addressed correctly (Rondinini et al. 2006; Beck et al. 2014; Kittle et al. 2018). To reduce the spatial bias, we first reduced the number of occurrences in oversampled regions using spatial filtering by aggregating points of occurrence into presences into equal‐area grid cells (Dormann et al. 2007; Phillips et al. 2009; Kramer‐Schadt et al. 2013; Aiello‐Lammens et al. 2015; Cimatti et al. 2021) of 1 × 1 km using a Conic Equal Area projection (Europe Albers Equal Area Conic). Each grid cell that contained at least one occurrence point was assigned a “1” (considering only once a pixel with one or more occurrences) (Cimatti et al. 2021). Furthermore, we excluded species with < 50 “presence” grid cells from the analyses. In addition, we applied a second technique/recommendation to reduce the spatial bias in GBIF data which consists in selecting the pseudo‐absences following the same spatial bias as presence data (Phillips et al. 2009; Wisz and Guisan 2009; Iturbide et al. 2015). To that end, following previous studies (Chefaoui and Lobo 2008; Iturbide et al. 2018), for each species we selected pseudo‐absences randomly among the 1 × 1 km grid cells within a 10 km buffer and outside a 3 km buffer around each pixel with presence calculated using Idrisi (Clark Labs 2012). Pseudo‐absences were assigned a “0,” and we selected the same number of pseudo‐absences as presences. We used climate and land‐use variables (Karger et al. 2017; Schipper et al. 2020) as predictors selected the variables to calculate the habitat suitability of food species following a three‐step procedure: (1) preselection of variables, (2) filtering of correlated variables, and (3) selection of best variables for each species (for a detailed description of the calculation of habitat suitability of food species see Appendix S1). Using these six selected variables, we modelled the habitat of each food species applying ensemble modelling, a statistical technique that improves the robustness of predictions (Araujo and New 2007), using the R package Biomod2 (Thuiller et al. 2016) (Appendix S1).

Using the fitted ensemble models, we predicted the habitat suitability of each food species for the current and three future coupled scenarios of climate and land use. Future scenarios coupled the climate data from the Institut Pierre Simon Laplace Model CM5A‐MR (IPSL‐CM5A‐MR) (Mignot and Bony 2013) from the CHELSA (Karger et al. 2017) database and land‐use forecasts from the GLOBIO 4 (Schipper et al. 2020) database for the year 2050 (Appendix S1).

From the IPSL‐CM5A‐MR from CHELSA, we selected the RCP2.6 scenario, RCP6.0 scenario, and the RCP8.5 scenario. Thus, combining the future scenarios from the GLOBIO 4 database and from CHELSA, we obtained three future socioeconomic shared pathways (SSPs); namely SSP1‐2.6, SSP3‐6.0, and SSP5‐8.5.

### 2.7 Brown Bear Presence Databases

We constructed two databases of brown bear presence: (1) a database of brown bear at the scale of its geographic range using historical distribution data, the *Range Database*, and (2) a database of brown bear using current data, the *Occurrence Database*.

The *Range Database* contains data, at a low spatial resolution (50 × 50 km), on the Eurasian historical distribution of brown bears, areas of current presence, and areas which were occupied in the past but where the species has been extirpated. We discarded the North American brown bear distribution due to large differences in life‐history traits between Eurasian and North American subspecies (Zedrosser et al. 2011; Penteriani and Melletti 2020) (Appendix S1 and Figure S6). Across the pixels of presences and absences, we applied a random environmentally stratified sampling procedure following section 7.4.3 in Guisan et al. (2017). Environmentally stratified sampling design consists in designing a method to sample the environmental space (e.g., temperature, precipitation). First, it is necessary to create stratums, a subset of the environmental space where the sampling will be applied. We used climate variables (*Clim_3*, *Clim_4*, *Clim_8*, and *Clim_9*) to create stratums, subsets of the environmental space with similar climate conditions, and then we selected an equal number of presences and absences by stratum (equal number variant). This last selection was used to model the distribution of brown bears at the range scale. The environmental stratified sampling design, although it represents a more complex approximation, has the advantage over the spatially random approach of being more likely to include rare stratums (Guisan et al. 2017).

The *Occurrence Database* contains > 3.2 million brown bear occurrences with an uncertainty of < 1 km^2^ in Europe and Türkiye, comprising data from 23 countries and 14 subpopulations (all European and Turkish subpopulations), and for the period 1989–2018 (Tables S9–S11 and Figures S2 and S6). Based on identified individuals and on estimations from each research group reporting the data, the *Occurrence Database* contains data from more than 3350 brown bear individuals (> 900 linked to GPS and VHF collared individuals, > 1200 linked to genetic analysis, and 1295 estimated based in expert knowledge; Appendix S1, Tables S9 and S10 show more information/detail of datasets including the number of individuals, original occurrences and sampling methods used). Most of these occurrences (98%) were obtained from telemetry, VHF, and GPS collars. The use of telemetry data within SDMs provides more objective information about species distribution compared to other methods (Dambach and Rödder 2011) but these data are highly spatially autocorrelated (SAC) and may produce pseudoreplication if this is not considered (Holloway and Miller 2017). Among the methods applied to reduce SAC and avoid pseudoreplication from telemetry data in SDMs, spatial filtering is probably the most widely used (Holloway and Miller 2017), and it allows avoiding this problem in other sources of data, for example, tracks or sightings (Lucas, Herrero, et al. 2016; Grilo et al. 2019), and the combination of different data sources (Bogdanović et al. 2023). Thus, we applied spatial filtering and aggregated the occurrences into presences in equal‐area grid cells of 1 × 1 km using a Conic Equal Area projection (Europe Albers Equal Area Conic), considering only once a grid with one or more occurrences. Each grid cell that contained at least one occurrence was assigned as “presence” (“1”). We obtained more than 100,000 grid cells with the presence of brown bears showing a spatial bias between the different subpopulations, with a minimum of 471 presences for the Türkiye subpopulation and a maximum of 45,119 presences for the Scandinavian subpopulation (Table S11 and Figure S2). Then, we excluded presences occurring in nonterrestrial systems (i.e., presences in lakes or seas). We applied a second filter to the grid cells with presences to reduce their SAC, to avoid overrepresentation of some subpopulations due to a larger number of individuals or greater sampling effort, and also because of computing limitations. Our second filter consisted in subsampling the presence data by selecting a maximum of 2000 presences for each subpopulation, and for subpopulations with less than 2000 presences available, we selected all presences available (Table S11). Pseudo‐absences were extracted randomly within a 5 km buffer around pixels with brown bear presences, and we selected the same number of pseudo‐absences as presences for each subpopulation. In total, we used 24,908 presences, which were split into two sets, one set to train the model (19,926 presences, 80%) and the other to validate the model (4982 presences, 20%). Proportionally, we reduced to a 0.77% our initial data, from 3.2 million occurrences to 24,908 grid presences. This reduction in filtering the data is between 1 and 3 orders of magnitude bigger than previous studies using GPS collar data to fit SDMs (Maiorano et al. 2015; Coxen et al. 2017; Chibeya et al. 2021; Bogdanović et al. 2023), and it supposes an average of approximately 7.4 grid presences per bear individual. The training data were used in all models using the *Occurrence Database* (for the comparison of biotic proxies explaining brown bear distribution and for modelling brown bear distribution at a fine scale with the Bayesian models, BMs).

### 2.8 Modelling the Potential Energy Available to the Brown Bear Across Space

To assess (ii) whether species' geographic distributions are better explained by quantitative or binary proxies of biotic interactions, we calculated two proxies to calculate spatial variables describing the biotic interactions between the brown bear and food species in its diet. The first was a quantitative measure of biotic interactions (*Biotic variables*) obtained by multiplying the rEDEC_SubpS_, the relative energy contribution described at the species level in each subpopulation (defined as parts of the distribution of the species that are isolated from others and/or present different environmental characteristics and/or conservation status; Figure 2a; See Appendix S1 and Figure S7) by the habitat suitability of each species, and then combining the values for each food category (Figure 2b). The second was a binary measure of biotic interactions (*Biotic_binary variables*) calculated by multiplying the current habitat suitability by 1 or 0 depending on, respectively, whether or not an interaction with a given food species was observed in each subpopulation, and then combining the values for each food category. For each food category, we fitted and evaluated (Akaike information criterion, AIC, based) two univariable SDMs explaining brown bear distribution using the brown bear *Occurrence Database*: (a) a model using *Biotic variables* as predictors and (b) a model using *Biotic_binary* variables as predictors.

### 2.9 Bayesian Brown Bear Species Distribution Model

To assess (iii), whether species' geographic distributions are better explained when combining biotic and abiotic factors, we fitted and evaluated (widely applicable information criterion, WAIC, based) three Bayesian models (BMs) to explain brown bear distribution using data from the brown bear *Occurrence Database* as a response variable: (1) a model with abiotic (climate and land‐use variables) and biotic predictors (using the best overall proxies, *Biotic variables* or *Biotic_binary*, from the previous univariable SDMs), (2) a model with abiotic predictors only, and (3) a model with biotic predictors only (Figure 1e). To minimize bias or the truncation of the environmental space when using only current data (Thuiller et al. 2004; Talluto et al. 2016), two of these BMs utilized historical range data (*Range Database*): the abiotic and biotic BM, and the abiotic BM. Historical range data was included using a Bayesian hierarchical model (BHM) which combined models with historical geographic range as a response variable and historical climate variables as predictors (see Section 2.10) and models with current data (see Section 2.11). For the SDMs explaining brown bear distribution, we used as climate variables bioclimatic variables, which are variables derived from monthly temperature and precipitation values with a biological meaning. We included isothermality, which is the mean diurnal range divided by the temperature annual range (*Clim_3*), temperature seasonality (*Clim_4*), mean temperature of the wettest quarter (*Clim_8*), and mean temperature of the driest quarter (*Clim_9*; Karger et al. 2017 for a detailed description). For land use, we included the rate of urban areas (*Urban*), the rate of broadleaved forested areas (*Broadleaved Forest*), the rate of coniferous areas (*Coniferous Forest*), and a measure of the rate of natural areas at landscape scale (a 11 × 11 km window; *Natural Landscape*). To address (iv), whether future range shifts differ when considering biotic factors or not, we used the best BMs and assessed changes in the potential distribution of the brown bear combining the three previous SSPs with three scenarios of change: (1) change in abiotic and biotic variables, (2) change in abiotic variables, and (3) change in biotic variables (Figure 1d).

### 2.10 Species Distribution Model of the Historical Range

We used a model of the historical distribution of the brown bear (based on historical range data and climate data) to inform models of the current distribution (Appendix S1). Specifically, we fitted a species distribution model with presences/absences from the *Range Database* (50 × 50 km resolution) as a function of historical bioclimatic variables (Appendix S1). We selected bioclimatic variables by first dropping those with a VIF over a threshold of 10 using the R package usdm (Guisan et al. 2017; Naimi 2017). We further refined the variables by selecting the four best historical climate variables (Table S12) on the basis of the AIC of univariate binomial GLMs (logit link) with linear and quadratic effects. We then fitted a final “historical” istribution model with a Bayesian binomial GLM using these four variables (*Clim_3*, *Clim_4*, *Clim_8* and *Clim_9*) and a stratified sampling of presences/absences from the brown bear *Range Database*. We extracted the mean and standard deviation of the parameter estimates for use as an informed prior for the models described below (Tables S13–S17 and Figures S8–S10).

### 2.11 Modelling and Predicting Brown Bear Distribution at a Fine Scale

We modelled the distribution of the brown bear at a high spatial resolution (1 *×* 1 km; Table S18) using the training selection of presences/pseudo‐absences from the brown bear *Occurrence Database* as the response variable (*N* presences = 19,926; *N* pseudo‐absences = 19,926; Tables S9–S11) and three types of predictor variables: climate, land‐use, and biotic. We selected four variables to include within each type. For climate variables, we selected the same four variables used in the model of the historical brown bear distribution (but with values for the current climate, obtained from the CHELSA database (Karger et al. 2017)). For land‐use and biotic variables, we selected the four best (AIC‐based) uncorrelated variables determined by a univariable GLMM (Tables S19–S21).

We fitted three Bayesian models (BMs) explaining brown bear distribution using different combinations of factors (abiotic, biotic, or both): (1) a model with abiotic (climate and land‐use variables) and biotic predictors (using biotic variables), (2) a model with abiotic predictors only, and (3) a model with biotic predictors only (Table S18). Note that only models including climate variables can use the historical priors, and thus the model based solely on biotic variables can only use current data. We also fitted a null model with only the intercept using only current presences/pseudo‐absences.

The two Bayesian hierarchical models (BHMs) which used historical range data (the model with abiotic and biotic predictors and the model with biotic predictors only) used noninformative priors for land use and biotic variables and informative priors based on the historical distribution model as defined above. We assumed equal confidence for the historical and current data, and thus the mean and SD of the parameter estimates from the historical distribution model were not modified and used directly as priors. The biotic model used noninformative priors for all variables. All models were Bayesian binomial GLMMs calculated with a logit link, using the Hamiltonian Monte Carlo algorithm in Stan (Stan Development Team 2023, mc‐stan.org). We used Stan with the rstan, rstanarm, and loo packages in R to fit and assess the diagnostics of the models (Stan Development Team 2020; Goodrich et al. 2022; Vehtari et al. 2022). Models were diagnosed by calculating the model coefficients (best estimates and their SE), Monte Carlo standard error (MCSE), confidence intervals (10%, 50% and 90%), number of effective sample size (*N*_eff_), and the potential scale reduction factor on split chains (*R*hat; at convergence *R*hat = 1). In addition, we plotted the Markov chain from each model parameter to check whether chains were stationary, whether the path stayed within the posterior distribution (the mean value of the chain is stable from the beginning to the end) and had a good mixing, whether each successive sample within each parameter is not highly correlated with the previous sample (there is a zig‐zag motion of each path) (McElreath 2020). We evaluated and compared the models using the widely applicable information criterion (WAIC) (Vehtari et al. 2017). The best model (based on the WAIC) was evaluated using the validation subset of the brown bear *Occurrence Database* (*N* presences = 4982; *N* pseudo‐absences = 4982; Table S11). As we used pseudo‐absences, we established a cutoff for the potential distribution based on the 90th percentile training presence (Liu et al. 2005; Bean et al. 2012), that is, leaving out 10% of the observed presences of the training dataset.

In addition to those models, and in order to evaluate whether there was an effect of combining different data, we fitted two simple Bayesian models (not hierarchical) for the two BHMs without considering the information of the historical range; we used noninformative priors for all predictors (Table S18).

We used the best model to predict the distribution of brown bear in all subpopulations for the current and nine future climate/land use change scenarios which considered combinations of three SSPs (SSP1‐2.6, SSP3‐6.0, and SSP5‐8.5) with changes in (1) abiotic and biotic variables, (2) abiotic variables, and (3) biotic variables. For the biotic variables, the climate and land‐use scenarios were used indirectly to predict the influence of climate and land use on the habitat of species in the brown bear diet, which was then summarized as energy available in the space as described above (Table S22). For the current and each of the nine future climate/land‐use change scenarios for the distribution of brown bear, we calculated different descriptors related to the conservation status of species (Lucas et al. 2019; Ramírez‐Delgado et al. 2022), such as area of the distribution, percentage of the distribution occupied, and distribution included in protected areas using the World Database of Protected Areas (UNEP and IUCN 2017).

To assess error propagation in the Bayesian hierarchical model using abiotic and biotic factors, we applied a sensitivity analysis over the biotic variables, a technique widely used in fields of ecology and climate change (Rogelj et al. 2012; Barabás et al. 2014). This technique allowed us to quantify how the uncertainty from the biotic variables affects the model's overall uncertainty. For this, we first find the extremes of the biotic variable input (i.e., Figure 1d), with a 95% confidence interval on the strength of the inputs. Then we rerun the Bayesian model with the two extremes to get an upper and lower bound for how much the biotic layer affects uncertainty in the outputs and calculated the current prediction for these new models. We found that estimates and predictions changed slightly (Tables S23–S26 and Figures S11 and S12); predictions showed a high correlation with the original prediction (correlation = 0.9999991 for the model using the lower bound and correlation = 0.9999994 for models using the upper bound). All statistical analyses were performed in the R program, versions 3.1.2 (R Development Core Team 2017) and 4.02 (R Development Core Team 2020) for Bayesian analysis.

## 3 Results

### 3.1 Spatial Variation of Biotic Interactions

From the literature search, we identified trophic interactions between the brown bear and 276 food species (Figure 1a; Table S5). In total, 76.8% of species were plants and 23.2% were animals (13.0% vertebrates, 10.1% invertebrates). When focusing only on trophic interactions described at the species level, we found that the relative energy contribution of each food category varied among subpopulations; for example, in the Scandinavian subpopulation, 51% of the energy was of vertebrate origin compared to just 4% for the East Balkan subpopulation (Tables S27–S29). Also, the proportion of energy from human‐derived sources (*n* = 36 species) strongly varied among subpopulations; for example, in the Karelian subpopulation, only 2% of the energy was from human‐derived sources compared to 93% for the East Balkan subpopulation (Figure 2b,c; Table S30).

**FIGURE 3:**
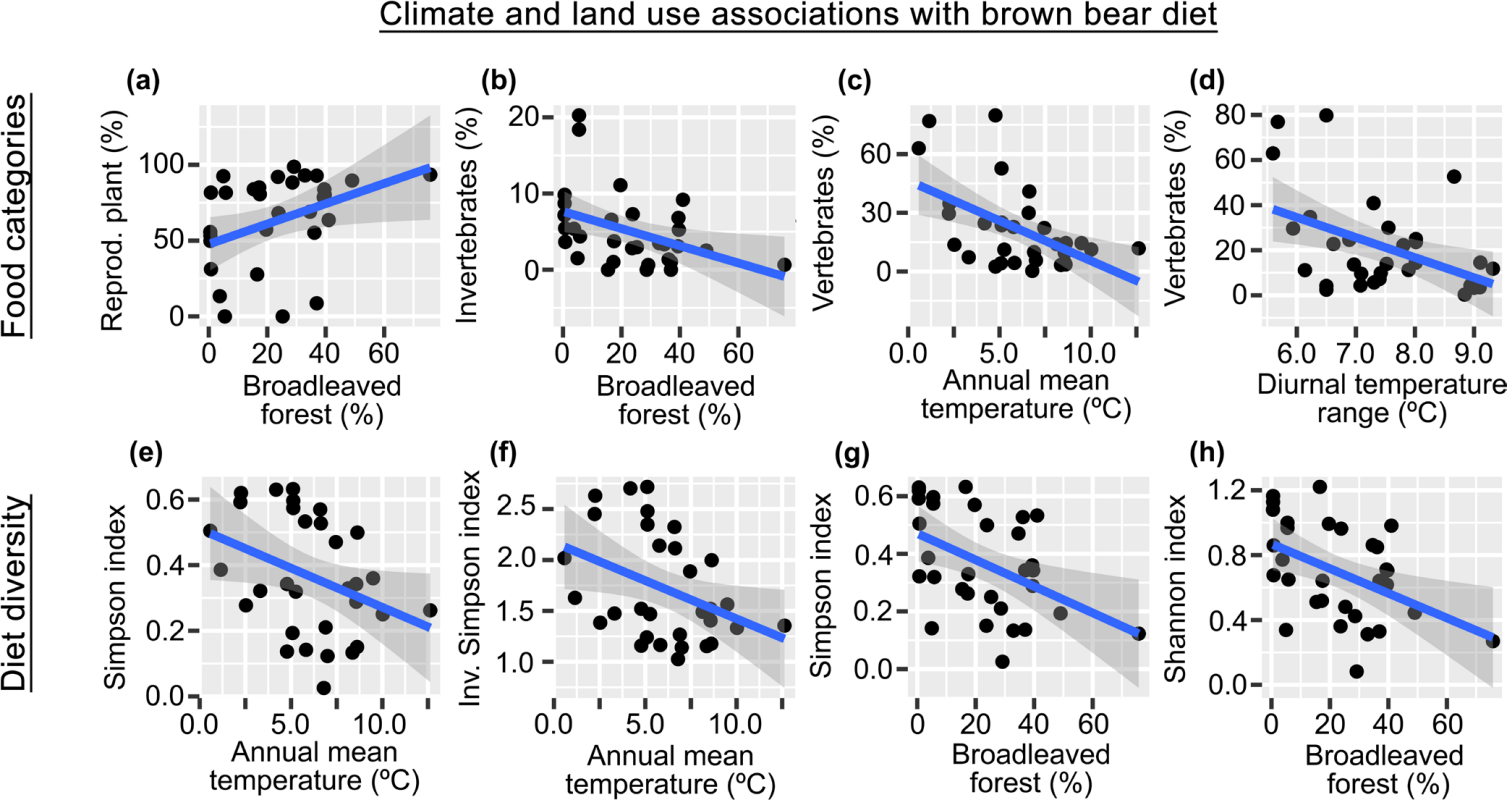
Linear models associating brown bear diet with environmental variables. (a) Association of relative estimated dietary energy content (rEDEC) from reproductive plant with percentage of broadleaved forest. (b) Association of rEDEC from invertebrates with percentage of broadleaved forest. (c) Association of rEDEC from vertebrates with annual mean temperature. (d) Association of rEDEC from vertebrates with diurnal temperature range. (e) Association of Simpson index of diversity (rEDEC‐based) with annual mean temperature. (f) Association of inverse Simpson diversity index (rEDEC‐based) with annual mean temperature 0. (g) Association of Simpson diversity index (rEDEC‐based) with percentage of broadleaved forest. (h) Association of Shannon diversity index (rEDEC‐based) with percentage of broadleaved forest. All predictors of these models showed significant associations (*p* values < 0.05).

Using all food items in each study site (*n* > 1300; not only those described at the species level), we found that the relative energy contribution to the diet of the brown bear for all food categories (apart from the vegetative plant category), as well as the diversity of food categories, was driven by climate and land use (climate and land‐use variables showed *p* values < 0.05; see Figure 3 and Tables S31–S50 for detailed model fits including parameter estimates and uncertainties and Appendix S2 for supplementary results). For example, bears consumed (based on the rEDEC) proportionally more reproductive plant parts (e.g., fleshy fruits and nuts) and fewer invertebrates in areas with more broadleaved forest cover (Figure 3a,b, respectively), and proportionally more vertebrates in areas with climates exhibiting lower annual mean temperatures (Figure 3c) or a smaller diurnal temperature range (Figure 3d). Similarly, bears tend to have more diverse diets when they occur in areas with lower annual mean temperature (Figure 3e,f) or in areas with lower broadleaved forest cover (Figure 3g,h).

### 3.2 Quantitative Versus Binary Proxies of Biotic Interactions to Explain Geographic Distributions

Among all wild food species (*n* = 240; Table S5), we were able to build robust SDMs and predict the current/future habitat suitability for 205 species (SDM_Food_: average sensitivity of 78.4%, specificity of 69.2% and TSS of 0.48; see Tables S8 and S51–S56 for detailed model fits). We then contrasted, based on AIC, whether brown bear distribution was better explained by quantitative (*Biotic variables*) or binary proxies of biotic interactions (*Biotic_binary*). For the vegetative plant food category, we found that the binary proxy for the interaction was sufficient to best explain brown bear distribution. Conversely, for all other food categories (i.e., reproductive plants, unknown plants, invertebrates, and vertebrates), including a quantitative measure of the interactions with species better explained the distribution of brown bears (see Table S21 for model comparison based on AIC).

### 3.3 The Role of Abiotic and Biotic Factors in Explaining Species' Geographic Distributions

**FIGURE 4:**
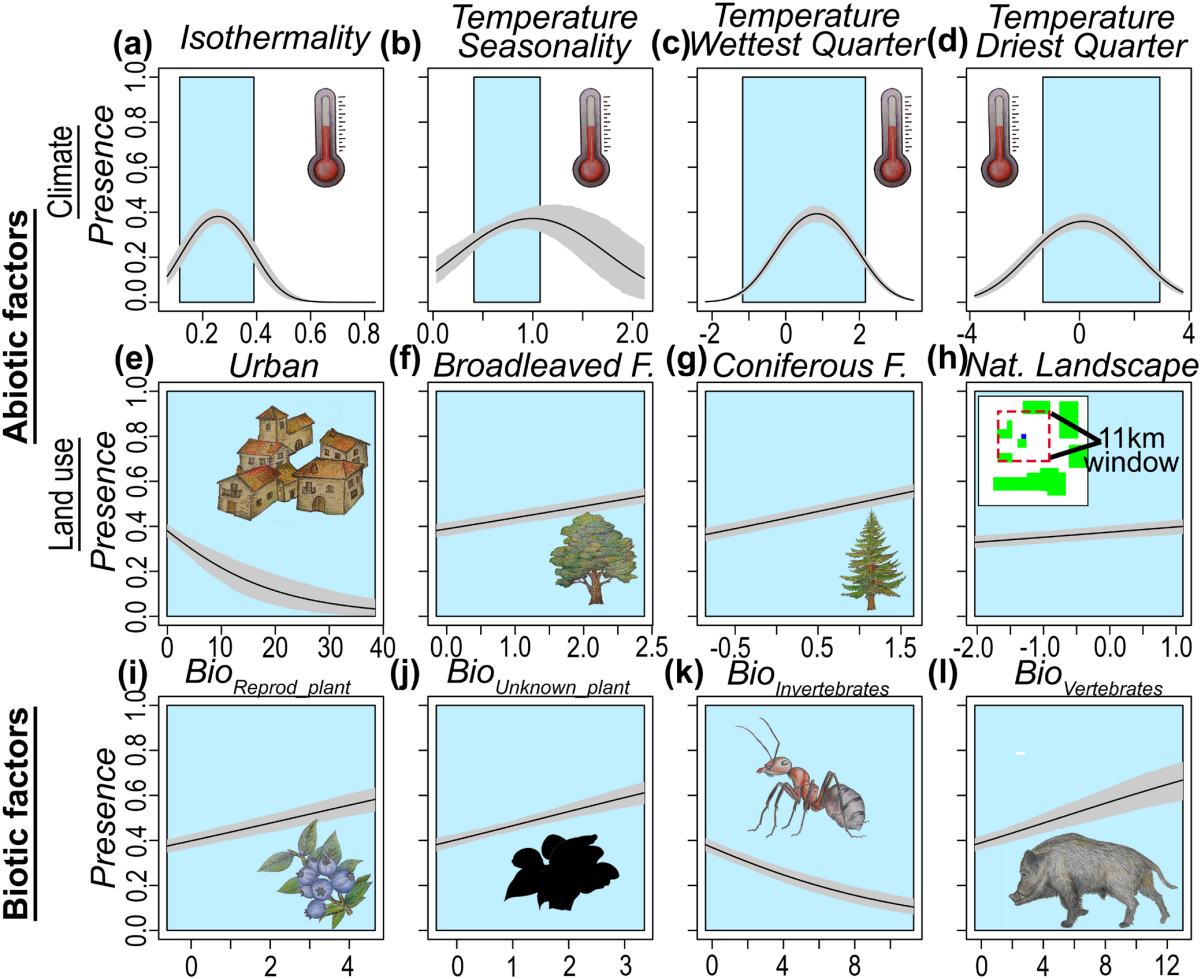
Partial response plots of brown bear distribution to both abiotic and biotic variables. The distribution model for brown bear including both abiotic and biotic factors was fitted combining both historical (*Range Database*) and current data (*Occurrence Database*). The continuous line represents the mean response value, and the grey area shows the model uncertainty (95% confidence interval). The blue area indicates the range of values of the current data. Isothermality (*Clim_3*), temperature seasonality (*Clim_4*), mean temperature of the wettest quarter (*Clim_8*), and mean temperature of the driest quarter (*Clim_9*).

**FIGURE 5:**
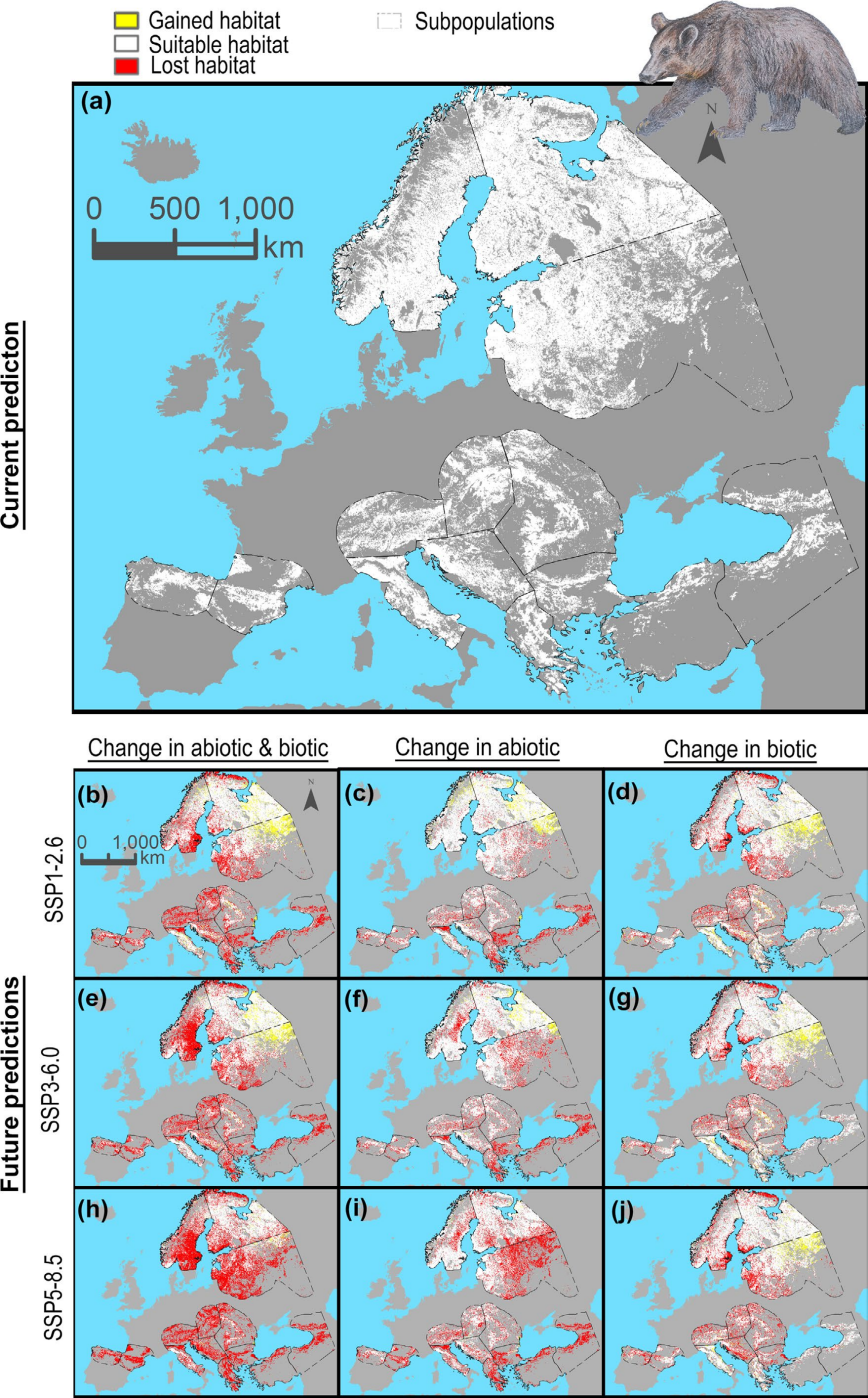
Brown bear habitat predictions. Prediction of brown bear habitat for current conditions (a). Future predictions of habitat for climate change scenarios SSP1‐2.6, SSP3‐6.0, and SSP5‐8.5 considering changes in both abiotic and biotic factors (b, e, h), changes in abiotic factors only (c, f, i), and changes in biotic factors only (d, g, j). The predicted area only includes a buffer area of 200 km around the current distribution to avoid extrapolating biotic variables into an environmental space where there is no information about the trophic interactions of brown bears.

When we compared the three Bayesian models (BMs), we found that the model combining abiotic and biotic factors was the best (WAIC_AbioticBiotic_ = 52,675.0 ± 93.1; Figure 4) and significantly improved the understanding of brown bear distribution compared to models using either abiotic or biotic factors (delta WAIC_Abiotic_ = 340.0 ± 95.5; delta WAIC_Biotic_ = 1679.9 ± 59.1; see Tables S18 and S57–S67 and Figures S11–S18 for detailed model fits including parameter estimates and uncertainties). The model combining abiotic and biotic factors showed a good performance compared with a Null model using only the intercept (delta WAIC_Null_ = 2539.7 ± 0.0) and yielded a high rate to correctly classify the presences of brown bear (true positive rate = 0.90; Table S68), with a low rate to correctly classify the pseudo‐absences of brown bear (true negative rate = 0.21), but see Leroy et al. (2018). The model threshold for classifying presence/absence was intentionally selected to have a true‐positive rate = 0.90 to overestimate the current distribution of the species as it has locally been eliminated from potentially suitable areas (Faurby and Araújo 2018) (see Methods), and similarly to other studies of large carnivores which have suffered important range contraction (Grilo et al. 2019). The predictions showed a current potential distribution for the brown bear of 2,794,314 km^2^ (Figure 5a; Table S69), with large areas that could host brown bears but currently do not.

In terms of species response to the selected abiotic and biotic variables, brown bear presence showed a bell‐shaped response to most climate variables, but as expected, a negative response to the percentage of urban areas, and a positive association with forests and natural landscapes (Figure 4). While the response of brown bear presence to most biotic variables/food categories was positive (i.e., reproductive plants, unknown plants, and vertebrates), it showed a surprising negative association with the invertebrate food category (see Section 4 and Table S58).

### 3.4 The Effect of Biotic Factors in Future Range Shifts

The predictions of biotic interactions, based on the best measures of interaction (*Biotic variables*), for future SSPs showed important differences. The potential available energy for the brown bear from all food species (*Bio*_*All_species*_) was predicted to be reduced by 53% under SSP3‐6.0. Importantly, when focusing on the different food categories, future predictions for biotic variables showed differences by food category and spatially varied by subpopulation (Figure 1d; Figure S19 and Table S22).

Those predicted future changes in biotic interactions affected the projected distribution of brown bears in the future. Our model showed a drastic range reduction that was more marked when considering both abiotic and biotic variables, with an overall reduction in 36%, as compared to either biotic (reduction in 13%) or abiotic (reduction in 20%) variables only. Range reduction was most pronounced in the south‐eastern subpopulations, for example, 93% reduction in the East Balkan subpopulation and 86% reduction in the Türkiye subpopulation (Figure 5; Tables S69–S72). Importantly, the spatial variability of future changes in biotic interactions and by food category described above translated into different effects across the brown bear range, as abiotic and biotic factors acted differently among the different subpopulations. For example, according to the SSP3‐6.0 scenario, in the Alpine subpopulation, biotic variables were associated with a habitat reduction in 44% compared to 32% for abiotic variables, whereas in Türkiye, biotic variables explained a comparatively smaller habitat reduction in 6% (Tables S69–S72).

## 4 Discussion

We demonstrate the importance of trophic interactions in explaining species distributions at large (continental) scales. Specifically, we found that (1) trophic interactions are highly variable across geographic space and are determined by climate and land‐use variables, (2) reliably estimating these biotic factors requires accounting for quantitative measures of biotic interactions, (3) including biotic interactions significantly improves our understanding of species distributions, and (4) the consideration of biotic interactions in future projections has important effects on predicting future consequences of climate and land‐use changes for species distributions and for ecosystem functioning. Our findings are based on a species with a wide abiotic niche, and which is a top‐predator diet generalist (Penteriani and Melletti 2020). Species with other ecological traits, such as different abiotic niche breadth, trophic position, diet breadth, or with a limited mobility capacity, might show different responses to land use and climate change (Carroll et al. 2024). Climate and human land use showed an association with biotic interactions through different ecosystems, suggesting that future scenarios of climate and land use may indirectly affect the brown bear diet. Climate and land use were used as proxies as they explain species richness and community (Coelho et al. 2023) which is the real/direct factor explaining biotic interactions. Future changes in brown bear food webs (our predator‐energy system) are caused by/based on overlap dynamics of predator abiotic niche and prey distributions (Durant et al. 2007; Carroll et al. 2024). At the continental level, two patterns prevail, divergent shifts, and contracted shifts, both causing a decrease in the available energy, but in some areas, we can observe congruous shifts, with bears and energy redistributing in similar directions (e.g., Scandinavian subpopulation) and convergent shifts, with bear available energy increasing the overlap with bear abiotic niche (e.g., Karelian subpopulation) (Durant et al. 2007; Carroll et al. 2024). This spatial variability, among the different subpopulations, in the overlap dynamic shows the complexity of the indirect effects of biotic interactions over species distributions and may be caused by several factors: (1) different magnitudes and directions of global changes in the different communities, for example, subpopulations around the Mediterranean will face higher increases in temperature and a reduction in precipitation (Karger et al. 2017); (2) there are different prey species, with different ecological traits, among the different subpopulations, for example, Southern subpopulations depend more on plant species with a narrower niche, whereas northern subpopulations have a higher proportion of ungulates in their diet. We did not account for species mobility, which may increase the contracted shifts for prey species of lower mobility. Overall, we would expect increasing homogenization in the diet among the different subpopulations, with more importance of those species with certain ecological traits such as a wide abiotic niche or higher mobility and a reduction in the available energy to bears in general (Carroll et al. 2024). However, reorganization of food webs implies different responses for top‐predators (Hoeks et al. 2020; Hao et al. 2025). As a diet generalist, the brown bear may switch to more available prey in response to a decline in overlap with important species in their diet (Ferreras et al. 2011) which could imply changes in the trophic position of the brown bear. In certain areas, energy reductions may lead to a decrease in bear abundance, as it has been observed in black bears (Laufenberg et al. 2018), and in some cases can contribute to a local extinction, joining other factors, for example, in subpopulations currently at high extinction risk or subpopulations facing an intense contraction of the abiotic niche (e.g., Türkiye subpopulation) (Sıkdokur et al. 2025). Importantly, changes in brown bear food webs can result in an increase in human–wildlife conflict if wild prey species availability decreases and bears switch to a diet based on anthropogenic sources (Chynoweth et al. 2016; Laufenberg et al. 2018; Abrahms et al. 2023; Kurth et al. 2024; Sıkdokur et al. 2024), which is a problem for the conservation of species (Pooley et al. 2017).

Ecosystem structure and functioning will be importantly affected by the reorganization of food webs driven by global changes (Sinclair 2003; Preisser et al. 2007). As large carnivores play a key role in ecosystems, a change in their abundance or trophic position determine important cascade effects, such as changes in abundance of herbivores and mesopredators and a decrease in the autotroph biomass (Hoeks et al. 2020). In the case of European and Türkiye ecosystems, the important range contraction of the historical distribution of brown bear (see historical range in Figure 1e and Figure S6) and its low abundance in some areas (several European subpopulations at extinction risk due to small population size) may have already caused an important change in ecosystem functionality and structure which should be considered for rewilding (Araújo and Alagador 2024).

The distribution of brown bears in Europe depends on climate variables, influencing the physiological functioning of the species (Bozinovic et al. 2011; Rogers et al. 2021; Kurth et al. 2024), as well as land use, such as forested areas and continuous natural areas which could be used as shelters (Grilo et al. 2019; Penteriani et al. 2019; Penteriani and Melletti 2020; Sıkdokur et al. 2025). This is in line with previous studies showing the importance for brown bear distribution of minimum temperature of the coldest month and annual mean temperature (Luna‐Aranguré et al. 2020; Sıkdokur et al. 2025) and with the importance of forest cover (Penteriani et al. 2019; Sıkdokur et al. 2025). However, here we show that even biotic factors shape brown bear distribution, represented here as the relative energy derived from food resources available to the species. According to our results, brown bears select areas that maximize this available energy (i.e., positive response of brown bear to most biotic variables), which may be explained by the high energy requirements of the species (White and Seymour 2003)^44^ and the absence of strong interspecific competition for food resources (Braz et al. 2020). The negative association with the availability of energy from invertebrates may be related to their negative correlation with isothermality (*Clim_3*; Correlation = −0.47), which has great importance for brown bear distribution and exhibits a bell‐shaped response (Figure 4a). This could indicate that areas with high available energy from invertebrate species are located in less suitable environments. In addition, the low relative energy represented by invertebrates in the brown bear diet—an average of 2% among all subpopulations (Figure 2b and Table S29)—may suggest that they represent opportunistic consumption rather than intentional/preferred prey, which may not influence the distribution of brown bears. Our results showed that brown bears currently have a large amount of suitable habitat that could be occupied. While the potential adaptability of brown bears to diverse food resources and the challenges of predicting future energy availability are promising research avenues, it is paramount to underscore the significant impact of humans on brown bear distribution and their conservation (Morales‐González et al. 2020; Ashrafzadeh et al. 2022). Furthermore, climate and land‐use changes have the potential to greatly reduce suitable bear habitats. Hence, safeguarding forests, minimizing landscape fragmentation, and preserving the species communities that interact with brown bears play vital roles in mitigating the effects of these drivers (Li et al. 2020).

The use of SDMs is one of the most advanced and widely used tools to understand the factors delimiting species distributions and to predict the effects of global change on biodiversity (Guisan and Thuiller 2005). Our results show the importance of additionally considering biotic factors and taking an ecosystem approach to properly understand species distributions (Romero et al. 2018; Antão et al. 2022), especially for modelling species distributions under climate and land‐use change scenarios (Thuiller et al. 2018; O'Gorman et al. 2023). However, as biotic interactions are highly complex, their inclusion needs to be accounted for on the basis of ecological studies that consider the spatial heterogeneity of these interactions and provide quantitative estimates for them (Banašek‐Richter et al. 2004; Banašek‐Richter et al. 2009). Our approximation was a simplification of the true biotic interaction networks, and other aspects such as competition, parasitism, the potential plasticity of different subpopulations to alter their diet in future scenarios, and/or other dimensions including temporal variation at seasonal, interannual, and other spatial scales may be relevant and could be considered in future studies. For example, the important diet adaptability of the brown bear and food preferences were not considered. It is expected that future communities where brown bears will be present will be different in species composition but also in their abundance, and this will have an important role in the potential interactions; for example, we would expect that brown bears would select species with higher energy such as big ungulates (Hayward and Kerley 2008; Niedziałkowska et al. 2019). It is also possible that novel habitats suitable for brown bears in both abiotic and biotic conditions could emerge under different climate change scenarios in areas currently outside brown bear distribution. In general, implementation of projections including biotic interactions in other species currently faces two big challenges: (1) detailed and extended information about ecological interaction networks, and (2) high‐quality data about species presences/occurrences. To overcome these challenges, global‐scale monitoring initiatives with open‐source principles, open‐source databases on species ecology, and the reduction of spatial and taxonomic biases will be of primary importance (Meyer et al. 2015; Delgado‐Baquerizo et al. 2016). This new generation of projections has a wider applicability over all species, allows decoupling abiotic and biotic factors, will better identify the drivers responsible for species distributions, and will enhance the predictions regarding the effects of global change on species, including agriculture or livestock species; overall they will generate important knowledge to conserve biodiversity, ecosystem services, and to secure the human food system.

## Author Contributions

**Pablo M. Lucas:** conceptualization, data curation, formal analysis, investigation, methodology, software, visualization, writing – original draft, writing – review and editing. **Wilfried Thuiller:** conceptualization, formal analysis, funding acquisition, methodology, resources, software, supervision, visualization, writing – original draft, writing – review and editing. **Lauren Talluto:** formal analysis, methodology, resources, software, visualization, writing – original draft, writing – review and editing. **Ester Polaina:** formal analysis, methodology, software, visualization, writing – original draft, writing – review and editing. **Jörg Albrecht:** data curation, formal analysis, funding acquisition, investigation, methodology, software, visualization, writing – review and editing. **Nuria Selva:** data curation, funding acquisition, investigation, project administration, writing – review and editing. **Marta De Barba:** data curation, funding acquisition, project administration, writing – review and editing. **Vincenzo Penteriani:** visualization, writing – original draft, writing – review and editing. **Maya Guéguen:** formal analysis, methodology, resources, software, writing – review and editing. **Niko Balkenhol:** funding acquisition, funding acquisition, writing – review and editing. **Trishna Dutta:** writing – review and editing. **Ancuta Fedorca:** funding acquisition, writing – review and editing. **Shane C. Frank:** investigation, writing – review and editing. **Andreas Zedrosser:** funding acquisition, investigation, writing – review and editing. **Ivan Afonso‐Jordana:** investigation, writing – review and editing. **Hüseyin Ambarlı:** investigation, writing – review and editing. **Fernando Ballesteros:** investigation, writing – review and editing. **Andriy‐Taras Bashta:** investigation, writing – review and editing. **Cemal Can Bilgin:** investigation, writing – review and editing. **Neda Bogdanović:** investigation, writing – review and editing. **Edgars Bojārs:** investigation, writing – review and editing. **Katarzyna Bojarska:** writing – review and editing. **Natalia Bragalanti:** investigation, writing – review and editing. **Henrik Brøseth:** data curation, investigation, writing – review and editing. **Mark W. Chynoweth:** investigation, writing – review and editing. **Duško Ćirović:** investigation, writing – review and editing. **Paolo Ciucci:** investigation, writing – review and editing. **Andrea Corradini:** investigation, writing – review and editing. **Daniele De Angelis:** data curation, investigation, writing – review and editing. **Miguel de Gabriel Hernando:** investigation, writing – review and editing. **Csaba Domokos:** investigation, writing – review and editing. **Aleksander Dutsov:** investigation, writing – review and editing. **Alper Ertürk:** investigation, writing – review and editing. **Stefano Filacorda:** investigation, writing – review and editing. **Lorenzo Frangini:** investigation, writing – review and editing. **Claudio Groff:** investigation, writing – review and editing. **Samuli Heikkinen:** investigation, writing – review and editing. **Bledi Hoxha:** investigation, writing – review and editing. **Djuro Huber:** investigation, writing – review and editing. **Otso Huitu:** investigation, writing – review and editing. **Georgeta Ionescu:** investigation, writing – review and editing. **Ovidiu Ionescu:** investigation, writing – review and editing. **Klemen Jerina:** investigation, writing – review and editing. **Ramon Jurj:** investigation, writing – review and editing. **Alexandros A. Karamanlidis:** investigation, writing – review and editing. **Jonas Kindberg:** data curation, investigation, writing – review and editing. **Ilpo Kojola:** investigation, writing – review and editing. **José Vicente López‐Bao:** investigation, writing – review and editing. **Peep Männil:** investigation, writing – review and editing. **Dime Melovski:** investigation, writing – review and editing. **Yorgos Mertzanis:** investigation, writing – review and editing. **Paolo Molinari:** investigation, writing – review and editing. **Anja Molinari‐Jobin:** investigation, writing – review and editing. **Andrea Mustoni:** investigation, writing – review and editing. **Javier Naves:** data curation, investigation, writing – review and editing. **Sergey Ogurtsov:** data curation, investigation, writing – review and editing. **Deniz Özüt:** investigation, writing – review and editing. **Santiago Palazón:** investigation, writing – review and editing. **Luca Pedrotti:** investigation, writing – review and editing. **Aleksandar Perović:** investigation, writing – review and editing. **Vladimir N. Piminov:** investigation, writing – review and editing. **Ioan‐Mihai Pop:** investigation, writing – review and editing. **Marius Popa:** investigation, writing – review and editing. **Maria Psaralexi:** investigation, writing – review and editing. **Pierre‐Yves Quenette:** investigation, writing – review and editing. **Georg Rauer:** investigation, writing – review and editing. **Slaven Reljic:** investigation, writing – review and editing. **Eloy Revilla:** formal analysis, investigation, writing – review and editing. **Urmas Saarma:** investigation, writing – review and editing. **Alexander P. Saveljev:** investigation, writing – review and editing. **Ali Onur Sayar:** investigation, writing – review and editing. **Çagan H. Şekercioğlu:** investigation, writing – review and editing. **Agnieszka Sergiel:** investigation, writing – review and editing. **George Sîrbu:** investigation, writing – review and editing. **Tomaž Skrbinšek:** investigation, writing – review and editing. **Michaela Skuban:** investigation, writing – review and editing. **Anil Soyumert:** investigation, writing – review and editing. **Aleksandar Stojanov:** investigation, writing – review and editing. **Egle Tammeleht:** investigation, writing – review and editing. **Konstantin Tirronen:** investigation, writing – review and editing. **Aleksandër Trajçe:** investigation, writing – review and editing. **Igor Trbojević:** investigation, writing – review and editing. **Tijana Trbojević:** investigation, writing – review and editing. **Filip Zięba:** investigation, writing – review and editing. **Diana Zlatanova:** investigation, writing – review and editing. **Tomasz Zwijacz‐Kozica:** investigation, writing – review and editing. **Laura J. Pollock:** conceptualization, formal analysis, methodology, resources, supervision, visualization, writing – original draft, writing – review and editing.

## Conflicts of Interest

The authors declare no conflicts of interest.

## Supporting information


Data S1.

## Data Availability

The data and R code that support the findings of this study are openly available in Zenodo at https://zenodo.org/records/15364702 (trophic interactions database), https://zenodo.org/records/15375279 (range database), https://doi.org/10.5281/zenodo.15336808 (R code) and Github at https://github.com/PabloMLucas/Trophiclinks‐shape‐global‐change‐effects (R code). This specimen represents an endangered or threatened species. The specific locality has been removed from the online record to protect this species from over‐collection. These data may be supplied to researchers on request. The database of occurrences of species in the brown bear diet obtained from GBIF, has been mirrored on Zenodo (https://doi.org/10.15468/dd.4whdmm). Temperature and precipitation data were obtained from CHELSA via Dryad at https://doi.org/10.5061/dryad.kd1d4. Land use/cover was obtained from GLOBIO 4 at https://www.globio.info/globio‐data‐downloads (Land use versions for 2015 and scenarios 2050 SSP1 RCP2.6, 2050 SSP3 RCP6.0 and 2050 SSP5 RCP8.5 from Schipper et al. 2020). Protected areas were obtained from the World Database on Protected Areas at https://www.protectedplanet.net/.

## References

[gcb70252-bib-0001] Abrahms, B. , N. H. Carter , T. J. Clark‐Wolf , et al. 2023. “Climate Change as a Global Amplifier of Human–Wildlife Conflict.” Nature Climate Change 13: 224–234. 10.1038/s41558-023-01608-5.

[gcb70252-bib-0002] Aiello‐Lammens, M. E. , R. A. Boria , A. Radosavljevic , B. Vilela , and R. P. Anderson . 2015. “spThin: An R Package for Spatial Thinning of Species Occurrence Records for Use in Ecological Niche Models.” Ecography 38: 541–545. 10.1111/ecog.01132.

[gcb70252-bib-0003] Antão, L. H. , B. Weigel , G. Strona , et al. 2022. “Climate Change Reshuffles Northern Species Within Their Niches.” Nature Climate Change 12: 587–592. 10.1038/s41558-022-01381-x.

[gcb70252-bib-0004] Araújo, M. B. , and D. Alagador . 2024. “Expanding European Protected Areas Through Rewilding.” Current Biology 34: 3931–3940.e5. 10.1016/j.cub.2024.07.045. 39151433

[gcb70252-bib-0005] Araujo, M. B. , and M. New . 2007. “Ensemble Forecasting of Species Distributions.” Trends in Ecology & Evolution 22: 42–47. 10.1016/j.tree.2006.09.010. 17011070

[gcb70252-bib-0006] Ashrafzadeh, M. R. , R. Khosravi , A. Mohammadi , et al. 2022. “Modeling Climate Change Impacts on the Distribution of an Endangered Brown Bear Population in Its Critical Habitat in Iran.” Science of the Total Environment 837: 155753. 10.1016/j.scitotenv.2022.155753. 35526639

[gcb70252-bib-0007] Banašek‐Richter, C. , L. F. Bersier , M.‐F. Cattin , et al. 2009. “Complexity in Quantitative Food Webs.” Ecology 90: 1470–1477. 10.1890/08-2207.1. 19569361

[gcb70252-bib-0008] Banašek‐Richter, C. , M. F. Cattin , and L.‐F. Bersier . 2004. “Sampling Effects and the Robustness of Quantitative and Qualitative Food‐Web Descriptors.” Journal of Theoretical Biology 226: 23–32. https://www.sciencedirect.com/science/article/pii/S0022519303003059. 14637051 10.1016/s0022-5193(03)00305-9

[gcb70252-bib-0009] Barabás, G. , L. Pásztor , G. Meszéna , and A. Ostling . 2014. “Sensitivity Analysis of Coexistence in Ecological Communities: Theory and Application.” Ecology Letters 17: 1479–1494. 10.1111/ele.12350. 25252135

[gcb70252-bib-0010] Bartley, T. J. , K. S. McCann , C. Bieg , et al. 2019. “Food Web Rewiring in a Changing World.” Nature Ecology & Evolution 3: 345–354. 10.1038/s41559-018-0772-3. 30742106

[gcb70252-bib-0011] Barton, K. 2009. “Mu‐MIn: Multi‐Model Inference.” R package version 0.12.2/r18. http://R‐Forge.R‐project.org/projects/mumin/.

[gcb70252-bib-0012] Bas, M. , J. Ouled‐Cheikh , A. Fuster‐Alonso , et al. 2025. “Potential Spatial Mismatches Between Marine Predators and Their Prey in the Southern Hemisphere in Response to Climate Change.” Global Change Biology 31: e70080. 10.1111/gcb.70080. 39968629

[gcb70252-bib-0013] Bean, W. T. , R. Stafford , and J. S. Brashares . 2012. “The Effects of Small Sample Size and Sample Bias on Threshold Selection and Accuracy Assessment of Species Distribution Models.” Ecography 35: 250–258. 10.1111/j.1600-0587.2011.06545.x.

[gcb70252-bib-0014] Beck, J. , M. Böller , A. Erhardt , and W. Schwanghart . 2014. “Spatial Bias in the GBIF Database and Its Effect on Modeling Species' Geographic Distributions.” Ecological Informatics 19: 10–15.

[gcb70252-bib-0015] Belmaker, J. , P. Zarnetske , M.‐N. Tuanmu , et al. 2015. “Empirical Evidence for the Scale Dependence of Biotic Interactions.” Global Ecology and Biogeography 24: 750–761. 10.1111/geb.12311.

[gcb70252-bib-0016] Blanchet, F. G. , K. Cazelles , and D. Gravel . 2020. “Co‐Occurrence Is Not Evidence of Ecological Interactions.” Ecology Letters 23: 1050–1063. 10.1111/ele.13525. 32429003

[gcb70252-bib-0017] Blois, J. L. , P. L. Zarnetske , M. C. Fitzpatrick , and S. Finnegan . 2013. “Climate Change and the Past, Present, and Future of Biotic Interactions.” Science 341: 499–504. 10.1126/science.1237184. 23908227

[gcb70252-bib-0018] Bogdanović, N. , A. Zedrosser , A. G. Hertel , A. Zarzo‐Arias , and D. Ćirović . 2023. “Where to Go? Habitat Preferences and Connectivity at a Crossroad of European Brown Bear Metapopulations.” Global Ecology and Conservation 43: e02460. 10.1016/j.gecco.2023.e02460.

[gcb70252-bib-0019] Boulangeat, I. , D. Gravel , and W. Thuiller . 2012. “Accounting for Dispersal and Biotic Interactions to Disentangle the Drivers of Species Distributions and Their Abundances.” Ecology Letters 15: 584–593. 10.1111/j.1461-0248.2012.01772.x. 22462813 PMC3999639

[gcb70252-bib-0020] Bozinovic, F. , P. Calosi , and J. I. Spicer . 2011. “Physiological Correlates of Geographic Range in Animals.” Annual Review of Ecology, Evolution, and Systematics 42: 155–179. 10.1146/annurev-ecolsys-102710-145055.

[gcb70252-bib-0021] Braga, J. , L. J. Pollock , C. Barros , et al. 2019. “Spatial Analyses of Multi‐Trophic Terrestrial Vertebrate Assemblages in Europe.” Global Ecology and Biogeography 28: 1636–1648. 10.1111/geb.12981.

[gcb70252-bib-0022] Braz, A. G. , C. E. de Viveiros Grelle , M. de Souza Lima Figueiredo , and M. M. Weber . 2020. “Interspecific Competition Constrains Local Abundance in Highly Suitable Areas.” Ecography 43: 1560–1570. 10.1111/ecog.04898.

[gcb70252-bib-0023] Carroll, G. , B. Abrahms , S. Brodie , and M. A. Cimino . 2024. “Spatial Match–Mismatch Between Predators and Prey Under Climate Change.” Nature Ecology & Evolution 8: 1593–1601. 10.1038/s41559-024-02454-0. 38914712

[gcb70252-bib-0024] Chefaoui, R. M. , and J. M. Lobo . 2008. “Assessing the Effects of Pseudo‐Absences on Predictive Distribution Model Performance.” Ecological Modelling 210: 478–486. 10.1016/j.ecolmodel.2007.08.010.

[gcb70252-bib-0025] Chen, I. C. C. , J. K. Hill , R. Ohlemüller , D. B. Roy , and C. D. Thomas . 2011. “Rapid Range Shifts of Species Associated With High Levels of Climate Warming.” Science 333: 1024–1026. 10.1126/science.1206432. 21852500

[gcb70252-bib-0026] Chibeya, D. , H. Wood , S. Cousins , K. Carter , M. A. Nyirenda , and H. Maseka . 2021. “How Do African Elephants Utilize the Landscape During Wet Season? A Habitat Connectivity Analysis for Sioma Ngwezi Landscape in Zambia.” Ecology and Evolution 11: 14916–14931. 10.1002/ece3.8177. 34765150 PMC8571614

[gcb70252-bib-0027] Chynoweth, M. W. , E. Çoban , Ç. Altın , and Ç. H. Şekercioğlu . 2016. “Human‐Wildlife Conflict as a Barrier to Large Carnivore Management and Conservation in Turkey.” Turkish Journal of Zoology 40: 972–983.

[gcb70252-bib-0028] Cimatti, M. , N. Ranc , A. Benítez‐López , et al. 2021. “Large Carnivore Expansion in Europe Is Associated With Human Population Density and Land Cover Changes.” Diversity and Distributions 27: 602–617. 10.1111/ddi.13219.

[gcb70252-bib-0029] Clark Labs . 2012. Idrisi 17.02. Clark Labs.

[gcb70252-bib-0030] Coelho, M. T. P. , E. Barreto , T. F. Rangel , et al. 2023. “The Geography of Climate and the Global Patterns of Species Diversity.” Nature 622: 537–544. 10.1038/s41586-023-06577-5. 37758942 PMC10584679

[gcb70252-bib-0031] Coxen, C. L. , J. K. Frey , S. A. Carleton , and D. P. Collins . 2017. “Species Distribution Models for a Migratory Bird Based on Citizen Science and Satellite Tracking Data.” Global Ecology and Conservation 11: 298–311. 10.1016/j.gecco.2017.08.001.

[gcb70252-bib-0032] Dambach, J. , and D. Rödder . 2011. “Applications and Future Challenges in Marine Species Distribution Modeling.” Aquatic Conservation: Marine and Freshwater Ecosystems 21: 92–100. 10.1002/aqc.1160.

[gcb70252-bib-0033] Delgado‐Baquerizo, M. , F. T. Maestre , P. B. Reich , et al. 2016. “Microbial Diversity Drives Multifunctionality in Terrestrial Ecosystems.” Nature Communications 7: 10541. 10.1038/ncomms10541. PMC4738359 26817514

[gcb70252-bib-0034] Dormann, C. F. , M. Bobrowski , D. M. Dehling , et al. 2018. “Biotic Interactions in Species Distribution Modelling: 10 Questions to Guide Interpretation and Avoid False Conclusions.” Global Ecology and Biogeography 27: 1004–1016. 10.1111/geb.12759.

[gcb70252-bib-0035] Dormann, C. F. , J. Elith , S. Bacher , et al. 2013. “Collinearity: A Review of Methods to Deal With It and a Simulation Study Evaluating Their Performance.” Ecography 36, no. 1: 27–46.

[gcb70252-bib-0036] Dormann, C. F. , J. M. McPherson , M. B. Araújo , et al. 2007. “Methods to Account for Spatial Autocorrelation in the Analysis of Species Distributional Data: A Review.” Ecography 30: 609–628. 10.1111/j.2007.0906-7590.05171.x.

[gcb70252-bib-0037] Dudenhöffer, J.‐H. , N. C. Luecke , and K. M. Crawford . 2022. “Changes in Precipitation Patterns Can Destabilize Plant Species Coexistence via Changes in Plant–Soil Feedback.” Nature Ecology & Evolution 6: 546–554. 10.1038/s41559-022-01700-7. 35347257

[gcb70252-bib-0038] Durant, J. M. , D. Ø. Hjermann , G. Ottersen , and N. C. Stenseth . 2007. “Climate and the Match or Mismatch Between Predator Requirements and Resource Availability.” Climate Research 33: 271–283.

[gcb70252-bib-0039] Esri Inc . 2016. ArcMap 10.5. Esri Inc.

[gcb70252-bib-0040] Faurby, S. , and M. B. Araújo . 2018. “Anthropogenic Range Contractions Bias Species Climate Change Forecasts.” Nature Climate Change 8: 252–256. 10.1038/s41558-018-0089-x.

[gcb70252-bib-0041] Ferreras, P. , A. Travaini , S. Cristina Zapata , and M. Delibes . 2011. “Short‐Term Responses of Mammalian Carnivores to a Sudden Collapse of Rabbits in Mediterranean Spain.” Basic and Applied Ecology 12: 116–124. 10.1016/j.baae.2011.01.005.

[gcb70252-bib-0042] Fisher, R. A. , A. S. Corbet , and C. B. Williams . 1943. “The Relation Between the Number of Species and the Number of Individuals in a Random Sample of an Animal Population.” Journal of Animal Ecology 12, no. 1: 42–58. 10.2307/1411.

[gcb70252-bib-0043] Galiana, N. , M. Lurgi , B. Claramunt‐López , et al. 2018. “The Spatial Scaling of Species Interaction Networks.” Nature Ecology & Evolution 2: 782–790. 10.1038/s41559-018-0517-3. 29662224

[gcb70252-bib-0044] GBIF . 2018. “The Global Biodiversity Information Facility. What is GBIF?” https://www.gbif.org/what‐is‐gbif.

[gcb70252-bib-0045] GBIF . 2023. “Derived Dataset GBIF.org (2023) Filtered Export of GBIF Occurrence Data.” 10.15468/dd.4whdmm.

[gcb70252-bib-0046] Goodrich, B. , J. Gabry , I. Ali , and B. S . 2022. “rstanarm: Bayesian Applied Regression Modeling via Stan.” R package version 2.21.3. https://mc‐stan.org/rstanarm/.

[gcb70252-bib-0047] Grilo, C. , P. M. Lucas , A. Fernández‐Gil , et al. 2019. “Refuge as Major Habitat Driver for Wolf Presence in Human‐Modified Landscapes.” Animal Conservation 22: 59–71. 10.1111/acv.12435.

[gcb70252-bib-0048] Guisan, A. , and W. Thuiller . 2005. “Predicting Species Distribution: Offering More Than Simple Habitat Models.” Ecology Letters 8: 993–1009. 10.1111/j.1461-0248.2005.00792.x. 34517687

[gcb70252-bib-0049] Guisan, A. , W. Thuiller , and N. E. Zimmermann . 2017. Habitat Suitability and Distribution Models: With Applications in R. Cambridge University Press.

[gcb70252-bib-0050] Hao, X. , M. Holyoak , Z. Zhang , and C. Yan . 2025. “Global Projection of Terrestrial Vertebrate Food Webs Under Future Climate and Land‐Use Changes.” Global Change Biology 31: e70061. 10.1111/gcb.70061. 39895400

[gcb70252-bib-0051] Hayward, M. W. , and G. I. H. Kerley . 2008. “Prey Preferences and Dietary Overlap Amongst Africa's Large Predators.” South African Journal of Wildlife Research 38, no. 2: 93–108. 10.3957/0379-4369-38.2.93.

[gcb70252-bib-0052] Hewitt, D. G. , and C. T. Robbins . 1996. “Estimating Grizzly Bear Food Habits From Fecal Analysis.” Wildlife Society Bulletin (1973–2006) 24: 547–550.

[gcb70252-bib-0053] Hoeks, S. , M. A. J. Huijbregts , M. Busana , M. B. J. Harfoot , J.‐C. Svenning , and L. Santini . 2020. “Mechanistic Insights Into the Role of Large Carnivores for Ecosystem Structure and Functioning.” Ecography 43: 1752–1763. 10.1111/ecog.05191.

[gcb70252-bib-0054] Holloway, P. , and J. A. Miller . 2017. “A Quantitative Synthesis of the Movement Concepts Used Within Species Distribution Modelling.” Ecological Modelling 356: 91–103. 10.1016/j.ecolmodel.2017.04.005.

[gcb70252-bib-0055] Hortal, J. , F. Bello , J. A. F. Diniz‐Filho , T. M. Lewinsohn , J. M. Lobo , and R. J. Ladle . 2015. “Seven Shortfalls That Beset Large‐Scale Knowledge of Biodiversity.” Annual Review of Ecology, Evolution, and Systematics 46, no. 1: 523–549. 10.1146/annurev-ecolsys-112414-054400.

[gcb70252-bib-0056] Hurlbert, S. H. 1971. “The Nonconcept of Species Diversity: A Critique and Alternative Parameters.” Ecology 52: 577–586. 10.2307/1934145. 28973811

[gcb70252-bib-0057] Hutchinson, G. E. 1957. “Population Studies—Animal Ecology and Demography—Concluding Remarks.” Cold Spring Harbor Symposia on Quantitative Biology 22: 415–427.

[gcb70252-bib-0058] IPCC . 2014. Climate Change 2013: The Physical Science Basis. Contribution of Working Group I to the Fifth Assessment Report of the Intergovermental Panel on Climate Change. Cambridge University Press.

[gcb70252-bib-0059] Iturbide, M. , J. Bedia , and J. M. Gutiérrez . 2018. “Background Sampling and Transferability of Species Distribution Model Ensembles Under Climate Change.” Global and Planetary Change 166: 19–29. 10.1016/j.gloplacha.2018.03.008.

[gcb70252-bib-0060] Iturbide, M. , J. Bedia , S. Herrera , O. del Hierro , M. Pinto , and J. M. Gutiérrez . 2015. “A Framework for Species Distribution Modelling With Improved Pseudo‐Absence Generation.” Ecological Modelling 312: 166–174. 10.1016/j.ecolmodel.2015.05.018.

[gcb70252-bib-0061] Karger, D. N. , O. Conrad , J. Böhner , et al. 2017. “Climatologies at High Resolution for the Earth's Land Surface Areas.” Scientific Data 4: 170122. 10.1038/sdata.2017.122. 28872642 PMC5584396

[gcb70252-bib-0062] Kittle, A. M. , A. C. Watson , A. C‐S , and D. W. Macdonald . 2018. “Forest Cover and Level of Protection Influence the Island‐Wide Distribution of an Apex Carnivore and Umbrella Species, the Sri Lankan Leopard (*Panthera pardus kotiya*).” Biodiversity and Conservation 27, no. 1: 235–263. 10.1007/s10531-017-1431-8.

[gcb70252-bib-0063] Kramer‐Schadt, S. , J. Niedballa , J. D. Pilgrim , et al. 2013. “The Importance of Correcting for Sampling Bias in MaxEnt Species Distribution Models.” Diversity and Distributions 19: 1366–1379. 10.1111/ddi.12096.

[gcb70252-bib-0064] Kurth, K. A. , K. C. Malpeli , J. D. Clark , H. E. Johnson , and F. T. van Manen . 2024. “A Systematic Review of the Effects of Climate Variability and Change on Black and Brown Bear Ecology and Interactions With Humans.” Biological Conservation 291: 110500. 10.1016/j.biocon.2024.110500.

[gcb70252-bib-0065] Laufenberg, J. S. , H. E. Johnson , P. F. Doherty , and S. W. Breck . 2018. “Compounding Effects of Human Development and a Natural Food Shortage on a Black Bear Population Along a Human Development‐Wildland Interface.” Biological Conservation 224: 188–198. 10.1016/j.biocon.2018.05.004.

[gcb70252-bib-0066] Lavergne, S. , N. Mouquet , W. Thuiller , and O. Ronce . 2010. “Biodiversity and Climate Change: Integrating Evolutionary and Ecological Responses of Species and Communities.” Annual Review of Ecology and Systematics 41: 321–350. 10.1146/annurev-ecolsys-102209-144628.

[gcb70252-bib-0067] Le Luherne, E. , L. Pawlowski , and M. Robert . 2024. “Northeast Atlantic Species Distribution Shifts Over the Last Two Decades.” Global Change Biology 30: e17383. 10.1111/gcb.17383. 38932518

[gcb70252-bib-0068] Leroy, B. , R. Delsol , B. Hugueny , et al. 2018. “Without Quality Presence–Absence Data, Discrimination Metrics Such as TSS Can Be Misleading Measures of Model Performance.” Journal of Biogeography 45, no. 9: 1994–2002. 10.1111/jbi.13402.

[gcb70252-bib-0069] Li, S. , W. J. McShea , D. Wang , et al. 2020. “Retreat of Large Carnivores Across the Giant Panda Distribution Range.” Nature Ecology & Evolution 4: 1327–1331. 10.1038/s41559-020-1260-0. 32747773

[gcb70252-bib-0070] Liu, C. , P. M. Berry , T. P. Dawson , and R. G. Pearson . 2005. “Selecting Thresholds of Occurrence in the Prediction of Species Distributions.” Ecography 25, no. 3: 385–393. 10.1111/j.0906-7590.2005.03957.x.

[gcb70252-bib-0071] Lucas, P. M. , M. González‐Suárez , and E. Revilla . 2019. “Range Area Matters, and So Does Spatial Configuration: Predicting Conservation Status in Vertebrates.” Ecography 42: 1103–1114. 10.1111/ecog.03865.

[gcb70252-bib-0072] Lucas, P. M. , M. González–Suárez , and E. Revilla . 2016. “Toward Multifactorial Null Models of Range Contraction in Terrestrial Vertebrates.” Ecography 39, no. 11: 1100–1108. 10.1111/ecog.01819.

[gcb70252-bib-0073] Lucas, P. M. , J. Herrero , and O. Fernández‐Arberas . 2016. “Modelling the Habitat of a Wild Ungulate in a Semi‐Arid Mediterranean Environment in Southwestern Europe: Small Cliffs Are Key Predictors of the Presence of Iberian Wild Goat.” Journal of Arid Environments 129: 56–63. 10.1016/j.jaridenv.2016.02.008.

[gcb70252-bib-0074] Luna‐Aranguré, C. , J. Soberón , and E. Vázquez‐Domínguez . 2020. “A Tale of Four Bears: Environmental Signal on the Phylogeographical Patterns Within the Extant Ursus Species.” Journal of Biogeography 47, no. 2: 472–486. 10.1111/jbi.13752.

[gcb70252-bib-0075] Maiorano, L. , L. Boitani , A. Monaco , E. Tosoni , and P. Ciucci . 2015. “Modeling the Distribution of Apennine Brown Bears During Hyperphagia to Reduce the Impact of Wild Boar Hunting.” European Journal of Wildlife Research 61: 241–253. 10.1007/s10344-014-0894-0.

[gcb70252-bib-0076] McElreath, R. 2020. Statistical Rethinking: A Bayesian Course With Examples in R and STAN. 2nd ed. Chapman and Hall/CRC.

[gcb70252-bib-0077] McLellan, B. N. , M. F. Proctor , D. Huber , and S. Michel . 2017. “*Ursus arctos* (Amended Version of 2017 Assessment).” The IUCN Red List of Threatened Species. https://www.iucnredlist.org/species/41688/121229971.

[gcb70252-bib-0078] Meier, E. S. , F. Kienast , P. B. Pearman , et al. 2010. “Biotic and Abiotic Variables Show Little Redundancy in Explaining Tree Species Distributions.” Ecography 33: 1038–1048. 10.1111/j.1600-0587.2010.06229.x.

[gcb70252-bib-0079] Meyer, C. , H. Kreft , R. Guralnick , and W. Jetz . 2015. “Global Priorities for an Effective Information Basis of Biodiversity Distributions.” Nature Communications 6: 8221. 10.1038/ncomms9221. PMC4569846 26348291

[gcb70252-bib-0080] Mignot, J. , and S. Bony . 2013. “Presentation and Analysis of the IPSL and CNRM Climate Models Used in CMIP5.” Climate Dynamics 40: 2089. 10.1007/s00382-013-1720-1.

[gcb70252-bib-0081] Morales‐González, A. , H. Ruiz‐Villar , A. Ordiz , and V. Penteriani . 2020. “Large Carnivores Living Alongside Humans: Brown Bears in Human‐Modified Landscapes.” Global Ecology and Conservation 22: e00937. 10.1016/j.gecco.2020.e00937.

[gcb70252-bib-0082] Naimi, B. 2017. “Package ‘usdm’ 1.1‐18. Uncertainty Analysis for Species Distribution Models.” CRAN.

[gcb70252-bib-0083] Niedziałkowska, M. , M. W. Hayward , T. Borowik , W. Jędrzejewski , and B. Jędrzejewska . 2019. “A Meta‐Analysis of Ungulate Predation and Prey Selection by the Brown Bear *Ursus arctos* in Eurasia.” Mammal Research 64: 1–9. 10.1007/s13364-018-0396-7.

[gcb70252-bib-0084] Nogues‐Bravo, D. 2009. “Predicting the Past Distribution of Species Climatic Niches.” Global Ecology and Biogeography 18: 521–531. 10.1111/j.1466-8238.2009.00476.x.

[gcb70252-bib-0085] O'Gorman, E. J. , L. Zhao , R. L. Kordas , S. Dudgeon , and G. Woodward . 2023. “Warming Indirectly Simplifies Food Webs Through Effects on Apex Predators.” Nature Ecology & Evolution 7: 1983–1992. 10.1038/s41559-023-02216-4. 37798434 PMC10697836

[gcb70252-bib-0086] Oksanen, J. , F. G. Blanchet , R. Kindt , et al. 2013. “Vegan‐Package. Community Ecology Package: Ordination, Diversity and Dissimilarities.” https://r‐forge.r‐project.org/projects/vegan/.

[gcb70252-bib-0087] O'Neill, B. C. , M. Oppenheimer , R. Warren , et al. 2017. “IPCC Reasons for Concern Regarding Climate Change Risks.” Nature Climate Change 7: 28–37. 10.1038/nclimate3179.

[gcb70252-bib-0088] Pacifici, M. , C. Rondinini , J. R. Rhodes , et al. 2020. “Global Correlates of Range Contractions and Expansions in Terrestrial Mammals.” Nature Communications 11: 2840. 10.1038/s41467-020-16684-w. PMC7275054 32504033

[gcb70252-bib-0089] Pacifici, M. , P. Visconti , S. H. M. Butchart , J. E. M. Watson , F. M. Cassola , and C. Rondinini . 2017. “Species' Traits Influenced Their Response to Recent Climate Change.” Nature Climate Change 7: 205–208. 10.1038/nclimate3223.

[gcb70252-bib-0090] Payne, A. R. D. , P. D. Mannion , G. T. Lloyd , and K. E. Davis . 2024. “Decoupling Speciation and Extinction Reveals Both Abiotic and Biotic Drivers Shaped 250 Million Years of Diversity in Crocodile‐Line Archosaurs.” Nature Ecology & Evolution 8: 121–132. 10.1038/s41559-023-02244-0. 38049481 PMC10781641

[gcb70252-bib-0091] Penteriani, V. , and M. Melletti . 2020. Bears of the World: Ecology, Conservation and Management. Cambridge University Press, Cambridge.

[gcb70252-bib-0092] Penteriani, V. , A. Zarzo‐Arias , A. Novo‐Fernández , G. Bombieri , and C. A. López‐Sánchez . 2019. “Responses of an Endangered Brown Bear Population to Climate Change Based on Predictable Food Resource and Shelter Alterations.” Global Change Biology 25: 1133–1151. 10.1111/gcb.14564. 30609226

[gcb70252-bib-0093] Pereira, H. M. , I. S. Martins , I. M. D. Rosa , et al. 2024. “Global Trends and Scenarios for Terrestrial Biodiversity and Ecosystem Services From 1900 to 2050.” Science 384: 458–465. 10.1126/science.adn3441. 38662818

[gcb70252-bib-0094] Phillips, S. J. , M. Dudik , J. Elith , et al. 2009. “Sample Selection Bias and Presence‐Only Distribution Models: Implications for Background and Pseudo‐Absence Data.” Ecological Applications 19: 181–197. 10.1890/07-2153.1. 19323182

[gcb70252-bib-0095] Pollock, L. J. , R. Tingley , W. K. Morris , et al. 2014. “Understanding Co‐Occurrence by Modelling Species Simultaneously With a Joint Species Distribution Model (JSDM).” Methods in Ecology and Evolution 5, no. 5: 397–406. 10.1111/2041-210X.12180.

[gcb70252-bib-0096] Pooley, S. , M. Barua , W. Beinart , et al. 2017. “An Interdisciplinary Review of Current and Future Approaches to Improving Human‐Predator Relations.” Conservation Biology 31: 513–523. 10.1111/cobi.12859. 27783450

[gcb70252-bib-0097] Preisser, E. L. , J. L. Orrock , and O. J. Schmitz . 2007. “Predator Hunting Mode and Habitat Domain Alter Nonconsumptive Effects in Predator‐Prey Interactions.” Ecology 88: 2744–2751. 10.1890/07-0260.1. 18051642

[gcb70252-bib-0098] R Development Core Team . 2017. R: A Language and Environment for Statistical Computing. R Foundation for Statistical Computing. http://www.R‐project.org.

[gcb70252-bib-0099] R Development Core Team . 2020. R: A Language and Environment for Statistical Computing. R Foundation for Statistical Computing. http://www.R‐project.org.

[gcb70252-bib-0100] Ramírez‐Delgado, J. P. , M. Di Marco , J. E. M. Watson , et al. 2022. “Matrix Condition Mediates the Effects of Habitat Fragmentation on Species Extinction Risk.” Nature Communications 13: 595. 10.1038/s41467-022-28270-3. PMC8807630 35105881

[gcb70252-bib-0101] Rogelj, J. , M. Meinshausen , and R. Knutti . 2012. “Global Warming Under Old and New Scenarios Using IPCC Climate Sensitivity Range Estimates.” Nature Climate Change 2: 248–253. 10.1038/nclimate1385.

[gcb70252-bib-0102] Rogers, S. A. , C. T. Robbins , P. D. Mathewson , et al. 2021. “Thermal Constraints on Energy Balance, Behaviour and Spatial Distribution of Grizzly Bears.” Functional Ecology 35: 398–410. 10.1111/1365-2435.13727.

[gcb70252-bib-0103] Romero, G. Q. , T. Gonçalves‐Souza , P. Kratina , et al. 2018. “Global Predation Pressure Redistribution Under Future Climate Change.” Nature Climate Change 8: 1087–1091. 10.1038/s41558-018-0347-y.

[gcb70252-bib-0104] Rondinini, C. , K. A. Wilson , L. Boitani , H. Grantham , and H. P. Possingham . 2006. “Tradeoffs of Different Types of Species Occurrence Data for Use in Systematic Conservation Planning.” Ecology Letters 9: 1136–1145. 10.1111/j.1461-0248.2006.00970.x. 16972877

[gcb70252-bib-0105] Schipper, A. M. , J. P. Hilbers , J. R. Meijer , et al. 2020. “Projecting Terrestrial Biodiversity Intactness With GLOBIO 4.” Global Change Biology 26: 760–771. 10.1111/gcb.14848. 31680366 PMC7028079

[gcb70252-bib-0106] Sıkdokur, E. , M. Naderi , E. Çeltik , et al. 2024. “Human‐Brown Bear Conflicts in Türkiye Are Driven by Increased Human Presence Around Protected Areas.” Ecological Informatics 81: 102643. 10.1016/j.ecoinf.2024.102643.

[gcb70252-bib-0107] Sıkdokur, E. , İ. K. Sağlam , Ç. H. Şekercioğlu , I. Kandemir , A. O. Sayar , and M. Naderi . 2025. “Climate‐Driven Range Shifts and Conservation Challenges for Brown Bears in Türkiye.” Ecology and Evolution 15: e71019. 10.1002/ece3.71019. 40177690 PMC11962206

[gcb70252-bib-0108] Sinclair, A. R. 2003. “Mammal Population Regulation, Keystone Processes and Ecosystem Dynamics.” Philosophical Transactions of the Royal Society of London. Series B, Biological Sciences 358: 1729–1740. 10.1098/rstb.2003.1359. 14561329 PMC1693264

[gcb70252-bib-0109] Stan Development Team . 2020. “RStan: The R Interface to Stan.” R package version 2.21.2. http://mc‐stan.org/.

[gcb70252-bib-0110] Stan Development Team . 2023. “Stan: Software for Bayesian Data Analysis.” mc‐stan.org.

[gcb70252-bib-0111] Talluto, M. V. , L. Talluto , I. Boulangeat , et al. 2016. “Cross‐Scale Integration of Knowledge for Predicting Species Ranges: A Metamodelling Framework.” Global Ecology and Biogeography 25, no. 2: 238–249. 10.1111/geb.12395. 27499698 PMC4975518

[gcb70252-bib-0112] Thuiller, W. , L. Brotons , M. B. Araujo , and S. Lavorel . 2004. “Effects of Restricting Environmental Range of Data to Project Current and Future Species Distributions.” Ecography 27: 165–172. 10.1111/j.0906-7590.2004.03673.x.

[gcb70252-bib-0113] Thuiller, W. , D. Georges , R. Engler , and F. Breiner . 2016. “Package ‘biomod2’. Ensemble Platform for Species Distribution Modeling.” CRAN.

[gcb70252-bib-0114] Thuiller, W. , M. Guéguen , M. Bison , et al. 2018. “Combining Point‐Process and Landscape Vegetation Models to Predict Large Herbivore Distributions in Space and Time—A Case Study of *Rupicapra rupicapra* .” Diversity and Distributions 24, no. 3: 352–362. 10.1111/ddi.12684.

[gcb70252-bib-0115] UNEP , and IUCN . 2017. “The World Database on Protected Areas (WDPA).” www.protectedplanet.net.

[gcb70252-bib-0116] Urban, M. C. , G. Bocedi , A. P. Hendry , et al. 2016. “Improving the Forecast for Biodiversity Under Climate Change.” Science 353: aad8466. 10.1126/science.aad8466. 27609898

[gcb70252-bib-0117] Vehtari, A. , J. Gabry , M. Magnusson , et al. 2022. “Loo: Efficient Leave‐One‐Out Cross‐Validation and WAIC for Bayesian Models.” R package version 2.5.0. https://mc‐stan.org/loo/.

[gcb70252-bib-0118] Vehtari, A. , A. Gelman , and J. Gabry . 2017. “Practical Bayesian Model Evaluation Using Leave‐One‐Out Cross‐Validation and WAIC.” Statistics and Computing 27: 1413–1432. 10.1007/s11222-016-9696-4.

[gcb70252-bib-0119] White, C. R. , and R. S. Seymour . 2003. “Mammalian Basal Metabolic Rate Is Proportional to Body Mass2/3.” Proceedings of the National Academy of Sciences of the United States of America 100: 4046–4049. 10.1073/pnas.0436428100. 12637681 PMC153045

[gcb70252-bib-0120] Willis, K. J. , and R. J. Whittaker . 2002. “Species Diversity: Scale Matters.” Science 295: 1245–1248. 11847328 10.1126/science.1067335

[gcb70252-bib-0121] Wisz, M. S. , and A. Guisan . 2009. “Do Pseudo‐Absence Selection Strategies Influence Species Distribution Models and Their Predictions? An Information‐Theoretic Approach Based on Simulated Data.” BMC Ecology 9: 8. 10.1186/1472-6785-9-8. 19393082 PMC2680809

[gcb70252-bib-0122] Wisz, M. S. , J. Pottier , W. D. Kissling , et al. 2013. “The Role of Biotic Interactions in Shaping Distributions and Realised Assemblages of Species: Implications for Species Distribution Modelling.” Biological Reviews 88: 15–30. 10.1111/j.1469-185X.2012.00235.x. 22686347 PMC3561684

[gcb70252-bib-0123] Zedrosser, A. , S. M. J. G. Steyaert , H. Gossow , and J. E. Swenson . 2011. “Brown Bear Conservation and the Ghost of Persecution Past.” Biological Conservation 144: 2163–2170. 10.1016/j.biocon.2011.05.005.

