# Supplementary material for: Trophic Interactions Are Key to Understanding the Effects of Global Change on the Distribution and Functional Role of the Brown Bear
Source: Glob Chang Biol. 2025 Jun 4;31(6):e70252. doi: 10.1111/gcb.70252 (PMC12134973; doi:10.1111/gcb.70252)
Supplement: Supplementary file 1 — Data S1. [file GCB-31-e70252-s001.zip › Supplementary_document.pdf]

Supplementary information

Title: Trophic interactions are key to understanding the effects of global change on the distribution and functional role of the brown bear

Running title: Trophic links shape global change effects

List of Authors: Pablo M. Lucas, Wilfried Thuiller, Lauren Talluto, Ester Polaina, Jörg Albrecht, Nuria Selva, Marta De Barba, Vincenzo Penteriani, Maya Guéguen, Niko Balkenhol, Trishna Dutta, Ancuta Fedorca, Shane C. Frank, Andreas Zedrosser, Ivan Afonso-Jordana, Hüseyin Ambarlı, Fernando Ballesteros, Andriy-Taras Bashta, Cemal Can Bilgin, Neda Bogdanović, Edgars Bojārs, Katarzyna Bojarska, Natalia Bragalanti, Henrik Brøseth, Mark W. Chynoweth, Duško Ćirović, Paolo Ciucci, Andrea Corradini, Daniele De Angelis, Miguel de Gabriel Hernando, Csaba Domokos, Aleksander Dutsov, Alper Ertürk, Stefano Filacorda, Lorenzo Frangini, Claudio Groff, Samuli Heikkinen, Bledi Hoxha, Djuro Huber, Otso Huitu, Georgeta Ionescu, Ovidiu Ionescu, Klemen Jerina, Ramon Jurj, Alexandros A. Karamanlidis, Jonas Kindberg, Ilpo Kojola, José Vicente López-Bao, Peep Männil, Dime Melovski, Yorgos Mertzanis, Paolo Molinari, Anja Molinari-Jobin, Andrea Mustoni, Javier Naves, Sergey Ogurtsov, Deniz Özüt, Santiago Palazón, Luca Pedrotti, Aleksandar Perović, Vladimir N. Piminov, Ioan-Mihai Pop, Marius Popa, Maria Psaralexi, Pierre-Yves Quenette, Georg Rauer, Slaven Reljic, Eloy Revilla, Urmas Saarma, Alexander P. Saveljev, Ali Onur Sayar, Çagan H. Şekercioğlu, Agnieszka Sergiel, George Sîrbu, Tomaž Skrbinšek, Michaela Skuban, Anil Soyumert, Aleksandar Stojanov, Egle Tammeleht, Konstantin Tirronen, Aleksandër Trajçe, Igor Trbojević, Tijana Trbojević, Filip Zięba, Diana Zlatanova, Tomasz Zwijacz-Kozica, Laura J. Pollock

TABLE OF CONTENT

Table of content for all items included in the supplementary information (in this and other files). We indicate the item, a description for each item, the name of the file where the item is included, it can be included in this file (Supplementary_document.pdf) or in an excel file and the thematic of the item.

**Item**	**Description**	**Name of the file where is located**	**Thematic**
Supplementary Appendix 1	Supplementary methods:<br>Calculation of the energy available from food items<br>Methodology to calculate the historical bioclimatic variables<br>Calculation of a representative diet for each subpopulation<br>Calculation of habitat suitability for each wild food species<br>Modelling the potential energy available to the brown bear across space<br>Brown bear presence databases<br>Bayesian brown bear species distribution model	Supplementary_document.pdf	Bear diet, brown bear SDMs
Supplementary Appendix 2	Supplementary results for diet	Supplementary_document.pdf	Supplementary results for diet
Supplementary Table 1	List of brown bear diet studies included in our database	Supplementary Table 1.xlsx	Bear diet
Supplementary Table 2	Database of diet of brown bear	Supplementary Table 2.xlsx	Bear diet
Supplementary Table 3	Coefficient factors (Cf1 and Cf2) associated to each category of diet used to convert *rV* to *rEDEC*	Supplementary Table 3.xlsx	Bear diet
Supplementary Table 4	This table is a subset of Supplementary Table 2 presenting only the infromation for the selected studies to account for current biotic intercations (See Supplementay Table 1)	Supplementary Table 4.xlsx	Bear diet
Supplementary Table 5	List of all species in the diet of brown bear considered with GBIF data of number of occurrences, species key, cleaned occurrences	Supplementary Table 5.xlsx	Bear diet
Supplementary Table 6	Name of bioclimatic variables and definition	Supplementary_document.pdf	Variables names
Supplementary Table 7	Categories of land cover/land use which were used in the analysis and original categories	Supplementary_document.pdf	Variables names
Supplementary Table 8	List of the 236 species in the diet of the brown for which it has been fitted a species distribution model	Supplementary Table 8.xlsx	SDMs species in the diet
Supplementary Table 9	Research groups which are sharing brown bear occurrences and description of the datasets received and processed	Supplementary Table 9.xlsx	Bear presences
Supplementary Table 10	The number of raw occurrences of brown bear by each type of data/source and group	Supplementary_document.pdf	Bear presences
Supplementary Table 11	The number of pixels of 1x1 km with presnce of brown bear by subpopulation	Supplementary_document.pdf	Bear presences
Supplementary Table 12	Comparasion of univarible models for historical bioclimatic variables	Supplementary_document.pdf	Range models brown bear
Supplementary Table 13	Results for the species distribution model at range scale, SDM_{Range} based on bayesian GLM predicting presence (using historical distribution in Eurasia) as a function of bioclimatic variables	Supplementary_document.pdf	Range models brown bear
Supplementary Table 14	Sample average posterior predictive distribution of the outcome for the species distribution model at range scale, SDM_{Range}	Supplementary_document.pdf	Range models brown bear

Item	Description	Name of the file where is located	Thematic
Supplementary Table 15	Estimates and standard error (SE) for the expected log pointwise predictive density (elpd_waic), the effective number of parameters (p_waic) and the information criterion waic (which is just -2 * elpd_waic, i.e., converted to deviance scale) for the species distribution model at range scale, SDM_{Range}.	Supplementary_document.pdf	Range models brown bear
Supplementary Table 16	Estimates and standard error (SE) for the expected log pointwise predictive density (elpd_waic), the effective number of parameters (p_waic) and the information criterion waic (which is just -2 * elpd_waic, i.e., converted to deviance scale) for a null species distribution model at range scale, SDM_{Range_Null}	Supplementary_document.pdf	Range models brown bear
Supplementary Table 17	Correlation of the posterior samples among the predictors used in the SDMRange	Supplementary Table 17.xlsx	Range models brown bear
Supplementary Table 18	Fitted bayesian models (BMs) explaining brown bear distribution using abiotic and/or biotic variables	Supplementary_document.pdf	Habitat models brown bear
Supplementary Table 19	Correlation among land use variables with current data of brown bear	Supplementary_document.pdf	Models land use
Supplementary Table 20	Univariable models of land use for select the best land use variables	Supplementary_document.pdf	Models land use
Supplementary Table 21	Comparasion of AIC values for univarible models including the biotic variables and the biotic variables binary and the null model with only the intercept	Supplementary_document.pdf	Biotic variables
Supplementary Table 22	Change in biotic variables	Supplementary Table 22.xlsx	Biotic variables
Supplementary Table 23	Results for the Bayesian hierarchical model using abiotic and biotic factors to explain brown bear distribution and the lower bound of biotic variables	Supplementary_document.pdf	Error propagation
Supplementary Table 24	Results for the Bayesian hierarchical model using abiotic and biotic factors to explain brown bear distribution and the upper bound of biotic variables	Supplementary_document.pdf	Error propagation
Supplementary Table 25	Correlation of the posterior samples among the predictors used in the Bayesian hierarchical model using abiotic and biotic factors to explain brown bear distribution and the lower bound of biotic variables	Supplementary Table 25.xlsx	Error propagation
Supplementary Table 26	Correlation of the posterior samples among the predictors used in the Bayesian hierarchical model using abiotic and biotic factors to explain brown bear distribution and the upper bound of biotic variables	Supplementary Table 26.xlsx	Error propagation
Supplementary Table 27	Matrix showing the summarized data of rEDEC for each species by subpopulation.	Supplementary Table 27.xlsx	Bear diet
Supplementary Table 28	rEDEC by subpopulation	Supplementary_document.pdf	Bear diet
Supplementary Table 29	rEDEC by subpopulation and diet category	Supplementary_document.pdf	Bear diet
Supplementary Table 30	rEDEC by subpopulation and human origin	Supplementary_document.pdf	Bear diet
Supplementary Table 31	Univariable models explaining the percentage of diet categories in all locations of studies	Supplementary Table 31.xlsx	Models explaining percentage of diet categories
Supplementary Table 32	Univariable models explaining the percentage of diet categories in the locations of selected studies	Supplementary Table 32.xlsx	Models explaining percentage of diet categories
Supplementary Table 33	Uncorrelated variables based on VIF with a threshold of 10	Supplementary Table 33.xlsx	Models explaining percentage of diet categories
Supplementary Table 34	Best multivariable models explaining the percentage of invertebrates obtained from the combination of all uncorrelated variables from table 33	Supplementary Table 34.xlsx	Models explaining percentage of diet categories
Supplementary Table 35	Best multivariable models explaining the percentage of reproductive plant material obtained from the combination of all uncorrelated variables from table 33	Supplementary Table 35.xlsx	Models explaining percentage of diet categories

Item	Description	Name of the file where is located	Thematic
Supplementary Table 36	Best multivariable models explaining the percentage of unknown plant material and others obtained from the combination of all uncorrelated variables from table 33	Supplementary Table 36.xlsx	Models explaining percentage of diet categories
Supplementary Table 37	Best multivariable models explaining the percentage of vegetative plant material obtained from the combination of all uncorrelated variables from table 33	Supplementary Table 37.xlsx	Models explaining percentage of diet categories
Supplementary Table 38	Best multivariable models explaining the percentage of vertebrates obtained from the combination of all uncorrelated variables from table 33	Supplementary Table 38.xlsx	Models explaining percentage of diet categories
Supplementary Table 39	Averaged model using the best multivariable models from table 34 explaining % invertebrates obtained from the combination of all uncorrelated variables from table 33	Supplementary Table 39.xlsx	Models explaining percentage of diet categories
Supplementary Table 40	Averaged model using the best multivariable models from table 35 explaining % reproductive plant material obtained from the combination of all uncorrelated variables from table 33	Supplementary Table 40.xlsx	Models explaining percentage of diet categories
Supplementary Table 41	Averaged model using the best multivariable models from table 36 explaining % unknown plant material and others obtained from the combination of all uncorrelated variables from table 33	Supplementary Table 41.xlsx	Models explaining percentage of diet categories
Supplementary Table 42	Averaged model using the best multivariable models from table 37 explaining % vegetative plant material obtained from the combination of all uncorrelated variables from table 33	Supplementary Table 42.xlsx	Models explaining percentage of diet categories
Supplementary Table 43	Averaged model using the best multivariable models from table 38 explaining % vertebrates obtained from the combination of all uncorrelated variables from table 33	Supplementary Table 43.xlsx	Models explaining percentage of diet categories
Supplementary Table 44	Diversity indexes of broen bear diet (Simpson, Shannon and inverse Simpsons) for study sites	Supplementary Table 44.xlsx	Models explaining diversity of diet
Supplementary Table 45	Best multivariable models explaining Shimpson doversity obtained from the combination of all uncorrelated variables from table 33	Supplementary Table 45.xlsx	Models explaining diversity of diet
Supplementary Table 46	Best multivariable models explaining Shanon doversity obtained from the combination of all uncorrelated variables from table 33	Supplementary Table 46.xlsx	Models explaining diversity of diet
Supplementary Table 47	Best multivariable models explaining inverse Simpsons doversity obtained from the combination of all uncorrelated variables from table 33	Supplementary Table 47.xlsx	Models explaining diversity of diet
Supplementary Table 48	Averaged model explaining the diet diversity of brown bear, with the Simpsons' diversity index, as a function of climate and land use variables.	Supplementary Table 48.xlsx	Models explaining diversity of diet
Supplementary Table 49	Averaged model explaining the diet diversity of brown bear, with the Shanon diversity index, as a function of climate and land use variables.	Supplementary Table 49.xlsx	Models explaining diversity of diet
Supplementary Table 50	Averaged model explaining the diet diversity of brown bear, with the inverse Simpsons' diversity index, as a function of climate and land use variables.	Supplementary Table 50.xlsx	Models explaining diversity of diet
Supplementary Table 51	Statistics, minimum, median, mean and maximum of the evaluations of species distribution models of species in the diet	Supplementary_document.pdf	SDMs species in the diet

Item	Description	Name of the file where is located	Thematic
Supplementary Table 52	Variable importance mean for the fitted species distribution models of species in the diet	Supplementary Table 52.xlsx	SDMs species in the diet
Supplementary Table 53	Variable importance mean for the fitted species distribution models of species in the diet	Supplementary_document.pdf	SDMs species in the diet
Supplementary Table 54	Habitat suitability change by species and subpopulation for the SSP1-2.6, SSP2-6.0 and SSP3-8.5 scenarios	Supplementary Table 54.xlsx	SDMs species in the diet
Supplementary Table 55	Mean habitat suitability change (in percentage) for each diet category and scenario	Supplementary_document.pdf	SDMs species in the diet
Supplementary Table 56	Mean habitat suitability change (in percentage) for all species by subpopulation and scenario.	Supplementary Table 56.xlsx	SDMs species in the diet
Supplementary Table 57	Performance for bayesian models (BMs) explaining brown bear distribution using abiotic and/or biotic variables	Supplementary_document.pdf	Habitat models brown bear
Supplementary Table 58	Results for the Bayesian hierarchical model using abiotic and biotic factors to explain brown bear distribution	Supplementary_document.pdf	Habitat models brown bear
Supplementary Table 59	Results for the simple Bayesian model (no hierarchical) using abiotic and biotic factors to explain brown bear distribution	Supplementary_document.pdf	Habitat models brown bear
Supplementary Table 60	Results for the Bayesian hierarchical model using abiotic factors to explain brown bear distribution	Supplementary_document.pdf	Habitat models brown bear
Supplementary Table 61	Results for the simple Bayesian model using abiotic factors to explain brown bear distribution	Supplementary_document.pdf	Habitat models brown bear
Supplementary Table 62	Results for the simple Bayesian model using biotic factors to explain brown bear distribution	Supplementary_document.pdf	Habitat models brown bear
Supplementary Table 63	Correlation of the posterior samples among the predictors used in the Bayesian hierarchical model using abiotic and biotic factors to explain brown bear distribution	Supplementary Table 63.xlsx	Habitat models brown bear
Supplementary Table 64	Correlation of the posterior samples among the predictors used in the simple Bayesian model (no hierarchical) using abiotic and biotic factors to explain brown bear distribution	Supplementary Table 64.xlsx	Habitat models brown bear
Supplementary Table 65	Correlation of the posterior samples among the predictors used in the Bayesian hierarchical model using abiotic factors to explain brown bear distribution	Supplementary Table 65.xlsx	Habitat models brown bear
Supplementary Table 66	Correlation of the posterior samples among the predictors used in the simple Bayesian model (no hierarchical) using abiotic factors to explain brown bear distribution	Supplementary Table 66.xlsx	Habitat models brown bear
Supplementary Table 67	Correlation of the posterior samples among the predictors used in the Biotic_current	Supplementary Table 67.xlsx	Habitat models brown bear
Supplementary Table 68	Results for the validation of the best Bayesian model (BM) explaining brown bear distribution, the Bayesian hierarchical model using abiotic and biotic factors	Supplementary Table 68.xlsx	Habitat models brown bear
Supplementary Table 69	Current potential habitat area, % of occupation, %protected areas in Europe	Supplementary Table 69.xlsx	Predictions best habitat model brown bear
Supplementary Table 70	Current potential habitat area, % of occupation, %protected areas by subpopulation	Supplementary Table 70.xlsx	Predictions best habitat model brown bear

Item	Description	Name of the file where is located	Thematic
Supplementary Table 71	Change in potential habitat area, % of occupation, %protected areas in Europe	Supplementary Table 71.xlsx	Predictions best habitat model brown bear
Supplementary Table 72	Change in potential habitat area, % of occupation, %protected areas by subpopulation	Supplementary Table 72.xlsx	Predictions best habitat model brown bear
Supplementary Figure 1	Detailed map showing the location of the brown bear diet studies with a code to join the list of sutides in Europe found in the review and the subpopulations	Supplementary_document.pdf	Bear diet
Supplementary Figure 2	Diagram showing the methodology	Supplementary_document.pdf	Methodology
Supplementary Figure 3	Bootstrapped correlation between relative frequency and relative volume	Supplementary_document.pdf	Bear diet
Supplementary Figure 4	Empirical relationships between relative frequency and relative volume	Supplementary_document.pdf	Bear diet
Supplementary Figure 5	Relationships between relative volume with relative frequency of observed and imputed data	Supplementary_document.pdf	Bear diet
Supplementary Figure 6	Historical distribution of brown bear in Eurasia used in the Species Distribution Model range	Supplementary_document.pdf	Range models brown bear
Supplementary Figure 7	Map showing the location of the brown bear presence data	Supplementary_document.pdf	Bear presences
Supplementary Figure 8	Chains for the Species Distribution Model at range scale, the SDM_{Range}	Supplementary_document.pdf	Range models brown bear
Supplementary Figure 9	Map at World scale with mean predicted probabilities brown bear distribution using model at range scale, SDM_{Range}	Supplementary_document.pdf	Range models brown bear
Supplementary Figure 10	Map at European scale with mean predicted probabilities brown bear distribution using model at range scale, SDM_{Range}	Supplementary_document.pdf	Range models brown bear
Supplementary Figure 11	Chains for the Bayesian hierarchical model using abiotic and biotic factors to explain brown bear distribution and the lower bound of biotic variables	Supplementary_document.pdf	Habitat models brown bear
Supplementary Figure 12	Chains for the Bayesian hierarchical model using abiotic and biotic factors to explain brown bear distribution and the upper bound of biotic variables	Supplementary_document.pdf	Habitat models brown bear
Supplementary Figure 13	Response plot of the three models, range scale, habitat scale and habitat combined	Supplementary_document.pdf	Habitat models brown bear
Supplementary Figure 14	Chains for the Bayesian model of brown bear habitat with abiotic and biotic factors combining data	Supplementary_document.pdf	Habitat models brown bear
Supplementary Figure 15	Chains for the Bayesian model of brown bear habitat with abiotic and biotic factors using current data	Supplementary_document.pdf	Habitat models brown bear
Supplementary Figure 16	Chains for the Bayesian model of brown bear habitat with abiotic factors combining data	Supplementary_document.pdf	Habitat models brown bear
Supplementary Figure 17	Chains for the Bayesian model of brown bear habitat with abiotic factors using current data	Supplementary_document.pdf	Habitat models brown bear
Supplementary Figure 18	Chains for the Bayesian model of brown bear habitat with biotic factors	Supplementary_document.pdf	Habitat models brown bear
Supplementary Figure 19	Maps with change in biotic variables for future scenarios	Supplementary_document.pdf	Biotic variables

Appendix S1

SUPPLEMENTARY METHODS

Calculation of the energy available from food items

We modelled the relationship between *rV* and *rF* based on studies in which both variables were available with a Bayesian hierarchical model that was fitted using the R package MCMCglmm (Hadfield 2010). We modelled the log(rV_{ij}) of food item *i* in study *j* (so one prey item can have multiple entries if it was found in multiple studies) using a normal distribution. We modelled log(rV_{ij}) as a function of log(rF_{ij}), diet category (i.e., vertebrate, invertebrate, seeds, fruits, vegetation or other [i.e., unidentified material or garbage]), and their interaction. We included a random intercept for study ID. We placed uninformative normal priors on the coefficients of the fixed explanatory variables (i.e., [0, 10^{10}]), and weakly informative inverse-gamma priors with shape = rate = 0.001 on the variance components (i.e., IG[0.001, 0.001]). The model was run for 130,000 iterations with a burn-in of 30,000 and a thinning interval of 100 iterations, resulting in 1,000 posterior samples. Convergence was checked using the potential scale reduction factor (PSRF < 1.1) (Gelman & Rubin 1992) and temporal autocorrelation (r < 0.1) using the R package coda (Plummer M et al. 2006). We used this model to impute *rV* for those studies with missing data. Then we applied two sets of correction factors to the estimates of *rV* to account for differences in digestibility and energy content between food items. We first applied correction factors for digestibility (CF_D; Supplementary Table 3) to calculate the relative dry weight of each food item *i* in each study (*rEDC*)(Pritchard & Robbins 1990) using the formula: $rEDC_i = CF_{Di} \times rV_i/\Sigma(CF_{Di} \times rV_i)$. Then we used a second set of correction factors (CF_E) to convert dry matter to digestible energy, and calculated the relative estimated dietary energy content (*rEDEC*) (Hewitt & Robbins 1996) of each food item *i* in each study: $rEDEC_i = CF_{Ei} \times rEDC_i/\Sigma(CF_{Ei} \times rEDC_i)$.

In diet studies, *rVi* and/or rF_i are often provided for groups of several species, and within these groups the species are usually described as present but without quantification of *rF* and/or *rV*, and thus the $rEDEC_i$ provides a description for a broad taxonomic group. To improve the

description at the species level, we followed a similar approach to previous food-web studies (Elgmork & Kaasa 1992; Stenset et al. 2016). For each group containing several species, we assigned its described $rEDEC_i$ to the species (s) that was most frequent in the bear diet, $rEDEC_s = rEDEC_i$, when explicitly stated in the paper. If several species belonging to the group were consumed by bears, but none explicitly mentioned as the most frequently eaten or occurring in scat, we divided equally the $rEDEC_i$ among the species consumed by bears and present in the area, $rEDEC_s = rEDEC_i/n_{species}$. For example, in Naves et al. (2006), we equally assigned $rEDEC_{Quercus}$ among the three *Quercus* species present and reported in the article as being part of the diet, $rEDEC_s$ = 22.07/3, thus each one was assigned 7.36%. We applied this method to items grouped at the genus level for plants and animals when the result was $rEDEC_s$ > 3% (Supplementary Tables 5 and 7).

Calculation of a representative diet for each subpopulation

The brown bear is a generalist omnivore species that shows high variation in its diet across its geographic distribution (Bojarska & Selva 2012). For example, the diet of bears in Scandinavia has a relatively higher proportion of vertebrates compared to individuals living in southern Europe, where the consumption of vegetation is comparatively much higher (Bojarska & Selva 2012). To consider this spatial variation in the brown bear diet, we calculated a representative diet of the brown bear for each *subpopulation* (*Subp*; Supplementary Figure 7). To differentiate brown bear subpopulations in Europe we used the definition of subpopulations provided by the IUCN, i.e., "geographically or otherwise distinct groups in a population between which there is little exchange". We utilized the current geographic distribution of brown bears (Linnell J. et al. 2008). Separated polygons were characterised as subpopulations and continuous polygons showing important differences in climate, habitat and conservation status of different spatial entities were split into subpopulations in order to account for this variability (e.g., Scandinavian, Karelian and Baltic subpopulations) (Linnell J. et al. 2008). Our assignment of subpopulations was similar to Chapron *et al. 2014*'s subdivisions for the brown bear, but we included two new entities for Europe. First, we considered *Pindos* a distinct subpopulation given its differences in climate and

habitat from the *Dinaric* and *East Balkan* subpopulations. Second, we considered *Western Carpathian* and *Eastern Carpathian* brown bears to represent different subpopulations on the basis of differences in habitat, climate and conservation status (Linnell J. et al. 2008; Straka et al. 2012). Third, two new subpopulations were considered for Turkey, *Turkey* and *Caucasus*. We assigned to each *subpopulation* a unique diet (D_S) on the basis of the reviewed studies for each subpopulation.

Calculation of habitat suitability for each wild food species

Global Biodiversity Information Facility (GBIF) data

To model habitat suitability of food species we used GBIF data. To this, we used the R package rgbif(Chamberlain S et al. 2018) to download occurrences of each food species from GBIF, a database which is widely used to assess the effects of climate change on biodiversity (Klonner et al. 2017; Dyderski et al. 2018) for species distribution modelling (Dallas et al. 2017; Filazzola et al. 2018) and species conservation (Mounce et al. 2017; Roll et al. 2017). We selected all occurrences of food species with coordinates, obtained from human or machine observations (e.g. camera traps), an uncertainty in meters of < 564 m to match the circumference radius of a circle of an area of our cell size (1km^2), located in Europe, North Africa and the Middle East (between decimal latitude “15, 75” and decimal longitude “-20, 105”) and for the period 1989–2018. Then, we used package CoordinateCleaner (Zizka et al. 2019) to remove usual errors in occurrences (i.e. country centroids).

Variable selection

We preselected a total of 11 environmental explanatory variables, 4 bioclimatic variables from the CHELSA dataset (Karger et al. 2017) following previous studies (Thuiller et al. 2019) (Supplementary Table 6), and then we preselected 7 land use/land cover variables representing the percent cover of different land use categories (Supplementary Table 7), obtained from the GLOBIO 4 dataset (Schipper et al. 2020). We excluded land use/land cover representing the percent cover of urban areas to avoid the introduction of a land cover category potentially

oversampled. We filtered, for each species, correlated variables by calculating the variance inflation factor (VIF) (Dormann et al. 2013) and excluding highly correlated variables (VIF > 10) using a stepwise procedure in the R package usdm (Naimi et al. 2014). We selected the final variables to include in the habitat models by fitting univariate Generalized Linear Models (GLMs) from the remaining uncorrelated variables and selecting the best 6 variables using an information theoretic approach based on Akaike Information Criterion (AIC) (Burnham & Anderson 2004).

Ensemble modelling

We applied ensemble modelling using for each species 12 models: (a) we repeated the selection of pseudo-absences twice, each time selecting the same number of random pseudo-absences as presences inside the 3-km and 10-km radius plots (as in the univariate models); (b) for each selection of pseudo-absences we repeated the process of data splitting twice (taking 70% of pseudo-absences to fit the model and 30% to evaluate); and (c) for each dataset of data splitting we fitted three different modelling algorithms: GLM with quadratic and second order polynomials allowed (for all predictors), Generalized Boosting Model/Boosted Regression Trees (GBM) with 3,000 trees and Random Forest (RF) with 750 trees. Each of the 12 fitted models (2 pseudo-absences selection x 2 data splitting x 3 modelling algorithms) was evaluated using the true skill statistic (TSS) (Allouche et al. 2006) and we selected models with TSS > 0.2 to include in the ensemble-model building.

Future scenarios to predict species habitat

Future scenarios to predict future habitat were obtained by using the climate data from the Institut Pierre Simon Laplace Model CM5A-MR (IPSL-CM5A-MR) (Mignot & Bony 2013) from the CHELSA database (Karger et al. 2017) and land use forecasts from the GLOBIO 4 database (Schipper et al. 2020) for the year 2050. GLOBIO 4 database uses three different scenarios, the SPP1_RCP2.6 scenario, the SPP3_RCP7.0 scenario and the SPP5_RCP8.5 scenario. These scenarios are based on a combination of three Shared Socioeconomic Pathways (SSPs), which are scenarios of projected socioeconomic global change up to the year 2100, including (1) the

Sustainability scenario, SSP1; (2) the Regional Rivalry scenario, SPP3; and (3) the Fossil-Fuelled Development scenario, SPP5 (Riahi et al. 2011; Dellink et al. 2017); with three Representative Concentration Pathways (RCPs) (Meinshausen et al. 2011): (1) the very stringent scenario, RCP2.6; (2) the intermediate stabilisation pathway scenario, RCP6.0; and (3) the scenario of comparatively high greenhouse gas emissions, RCP8.5 (Riahi et al. 2011; van Vuuren et al. 2011; IPCC 2014).

Modelling the potential energy available to the brown bear across space

Calculating potential biotic interactions on the basis of quantitative links: Biotic variables

We used the previously calculated $rEDEC_{SubpS}$ (See section *Calculation of a representative diet for each subpopulation*) as a link representing the interaction strength between food species *S* and the consumer, the brown bear, among the subpopulation *Subp*.

The spatial distribution of these interactions is heterogeneous across the landscape as it depends on the co-occurrence of the food species and the consumer, and a higher co-occurrence of both species will be positively associated with interaction strength (Banašek-Richter et al. 2009). Thus, within each subpopulation, we assigned the calculated $rEDEC_{SubpS}$ as a potential link across the predicted habitat of food species *S*, multiplying the $rEDEC_{SubpS}$ by the habitat suitability of food species *S*. Thus, for each grid cell (*C*) of each *subpopulation* we calculated a term called *Potential energy* (*Pe*) as:

$$Pe_{SubpSC} = rEDEC_{SubpS}\, Hs_{SC} \times 100$$

where:

Pe_{SubpSC} is the potential energy in the subpopulation *Subp* for species *S* in cell *C*,

Hs_{SC} is the habitat suitability of species *S* in cell *C*.

We considered groups of food species (i.e., reproductive plants, vegetative plants, unknown plants, invertebrates and vertebrates, and a group including all food species) which are vital for the brown bear and calculated the *Sum of the Potential energy* for all food species in each group *G* as:

$$SPe_{SubpGC} = \sum_{S=1}^{n} Pe_{SubpSC}$$

where:

SPe_{SubpGC} is the sum of the potential energy in the subpopulation *Subp* for all food species in group *G* in cell *C*,

n is the number of food species in each subpopulation *Subp* and group *G*,

G represents each of the groups of food species in the diet considered (reproductive plants, vegetative plants, unknown plants, invertebrates and vertebrates) and a group including all food species.

Using the SPe_{SubpGC} over all cells of our study area, we obtained the *Biotic variables*, which represent a spatial downscaling of the potential interaction strength among the food species of each diet group considered. Thus, we obtained six *Biotic variables:* $Bio_{All\_species}$, Bio_{Reprod_plant}, Bio_{Veget_plant}, $Bio_{Unknown\_plant}$, $Bio_{Invertebrates}$, and $Bio_{Vertebrates}$.

Calculating potential biotic interactions on the basis of binary links: Biotic_binary variables

We used a categorical/binary description for each subpopulation, $Binary_{SubpS}$, as a link representing the interaction strength between food species *S* and the brown bear within the subpopulation *Subp*. $Binary_{SubpS}$ has the binary value 1 when there was consumption of food species *S* by brown bear in the *Subp*, and 0 when there was no consumption of food species *S* by brown bears in the *Subp*.

In this case, within each *subpopulation*, we assigned the calculated $Binary_{SubpS}$ as a potential link over the predicted habitat of food species *S*, multiplying the $Binary_{SubpS}$ by the habitat suitability of food species *S*. Thus, for each grid cell (*C*) of each *subpopulation* we calculated a term called *Potential energy* (*Pe*) as:

$$Pe_{SubpSCBinary} = Binary_{SubpS} \times Hs_{SC}\ \ 100$$

where:

$Pe_{SubpSCBinary}$ is the potential energy in the subpopulation *Subp* for food species *S* in cell *C*,

Hs_{SC} is the habitat suitability of food species *S* in cell *C*.

We used the same diet groups of food species considered above for *Biotic variables*, and we calculated the *Sum of the Potential energy Binary* for all food species in each group *G* as:

$$SPe_{SubpGCBinary} = \sum_{S=1}^{n} Pe_{SubpSCBinary}$$

where:

$SPe_{SubpGCBinary}$ is the sum of the potential energy in the subpopulation *Subp* for all food species in group *G* in cell *C*,

n is the number of food species in each subpopulation *Subp* and group *G*,

G represents each of the groups of food species in the diet considered (reproductive plants, vegetative plants, unknown plants, invertebrates and vertebrates) and a group including all species.

Using the $SPe_{SubpGCBinary}$ over all cells of our study area we obtained the *Biotic_binary variables*, which represents a spatial downscaling of the potential interaction strength among the food species in each diet group considered. Thus, we obtained six *Biotic_binary variables:* $Bio_{B\_All\_species}$, $Bio_{B\_Reprod\_plant}$, $Bio_{B\_Veget\_plant}$, $Bio_{B\_Unknown\_plant}$, $Bio_{B\_Invertebrates}$, and $Bio_{B\_Vertebrates}$.

Comparison of biotic proxies explaining brown bear distribution

To assess which proxies of biotic interactions better explained brown bear distribution, we compared for each group *G*, the univariate models for *Biotic variables and Biotic_binary variables*. Models were fitted using a frequentist binomial GLMM with subpopulation as random factor and the brown bear *Occurrence Database* as presences/pseudo-absences (Supplementary Table 11, Supplementary Figure 7). We compared the univariate models using Akaike Information Criterion (AIC) (Burnham & Anderson 2004).

Brown bear presence databases

Range Database

We used the IUCN historical distribution of the brown bear (McLellan et al. 2017) and from several sources of published data and historical references (Parde & Camarra 1992; Servheen et al. 1999; Posillico et al. 2004; Valverde 2009; Álvares & Domingues 2010; Naves & Fernández-Gil 2017; Bencatel et al. 2018) (Supplementary Figure 6). The brown bear is a species that has suffered intense human persecution resulting in multiple local extinctions over the last two millennia(Lorenzen et al. 2011). Thus, we included information of past brown bear distribution since the 1st century, even though human-induced local extirpation of brown bears could have occurred before, e.g., in areas surrounding the Mediterranean region (Albrecht et al. 2017).

We used 10 different sources of historical data for brown bear, below we report the area of study, the map/figure/data within the publication and the century: for Spain we used data obtained from "*Anotaciones al "Libro de la Montería" del Rey Alfonso XI*. "(Valverde 2009) (XIV Century), for North West of Spain (Galicia) the figure 1 from "*A conservación do oso pardo en Galicia*" (XVIII Century) (Naves & Fernández-Gil 2017), for North of Spain (Cantabrian Mountains) the figure 6.16 from "*Bears. Status Survey and Conservation Action Plan*" (XIX Century) (Servheen et al. 1999) (XIX Century), for Portugal we used figure 1 from "*Research trends and geographical distribution of mammalian carnivores in Portugal*" (SW Europe) (Bencatel et al. 2018) (XVII Century), for Portugal we also used the figure 1 from "*Historical presence of Brown bear in Portugal and evidence of its relation with human communities*" (Álvares & Domingues 2010) (XVI-XVIII Centuries)(Álvares & Domingues 2010), for France we used the data from figure "*Répartition de lÓurs brun en France" from the "Encyclopédie des carnivores de France*"(Parde & Camarra 1992) (XV-XVI Centuries), figure 1 from "*A habitat model for brown bear conservation and land use planning in the central Apennines*" (XVI-XVIII Centuries)(Posillico et al. 2004), for the former Yugoslavian countries from figure 6.19 from "*Bears. Status Survey and Conservation Action Plan*" (XIX Century) (Servheen et al. 1999), for Greece from figure 6.8 from "*Bears. Status Survey and Conservation Action Plan*" (II Century)

(Servheen et al. 1999), and the historical distribution for brown bear for Eurasia from the IUCN Red List (XVI-XX Centuries) (McLellan et al. 2017).

Absences were extracted randomly within each study area and in the case of the historical distribution from the IUCN Red List we selected the absences within 2000 km buffer around pixels with brown bear presences in the historical distribution, and we selected the same number of absences as presences. Over these groups of presences and absences we will apply the random environmentally stratified sampling procedure.

Occurrence Database

To construct the *Occurrence Database*, we used 53 original datasets from 36 research groups with more than 3.2 million occurrences from diverse sources: 97.56% from GPS collars, 0.53% from VHF collars, 1.12% from tracks, 0.01% from camera traps and 0.01% from unspecified sources (Supplementary Tables 9 and 10). We only used data obtained from systematic survey methods, e.g. excluding locations of occasional sightings of bear damage, hunted bears and road/train casualties and/or monitoring data from problematic bears (e.g., bears selected because they frequently approach human settlements, or frequently attack livestock and/or cause other types of damage, but we did not exclude randomly monitoring bears that exhibited problematic behaviour) to avoid bias in the data towards problematic bears and/or more human used areas.

Bayesian brown bear species distribution model

Calculation of historical bioclimatic variables

We download data from the CHELSA-TraCE21k (Karger et al 2023) which provides monthly climate data for temperature and precipitation at 30 arcsec spatial resolution in 100-yeartime steps for the last 21,000 years (https://chelsa-climate.org/chelsa-trace21k/). For each historical distribution we obtained bioclimatic historical variables matching the centuries of the historical distribution and averaging the values of the bioclimatic variables in such period (See above *Range Database* section within Supplementary Appendix 1). Data originally at 1x1 km resolution were

projected into Cylindrical Equal Area and then aggregated at the resolution of the range scale (50x50 km).

Species distribution model of the historical range

Data at the range scale are of low resolution but larger extent, which capture a greater variability of the effect on species presence of factors acting at larger scales, e.g., climate, and avoid bias or the truncation of the environmental space (Thuiller et al. 2004; Faurby & Araújo 2018). Data at the habitat scale are of high resolution but small extent, which improve spatial precision and our understanding of the effect on species presence of factors acting at smaller scales. As we know that the brown bear distribution has contracted as a result of human activity, we wanted to explore how the historical relationship of brown bear occurrences with climate variables might influence our distribution models. For example, if the brown bear historically thrived in warmer conditions, then this information could also help us better predict its current and future distribution by avoiding a truncation of environmental variables (Faurby & Araújo 2018).

Appendix S2

SUPPLEMENTARY RESULTS

Associations between diet and environmental variables

Among climate variables, the mean diurnal temperature range (*Clim_2*) had the most important role in explaining the share of different food categories in brown bear dietary energy content (Supplementary Tables 39-43). In areas with higher diurnal temperature range, bears consumed less vegetative plant material, less unknown plant material and fewer vertebrates. The precipitation affected bear feeding habits to a lesser extent than temperature. The share of the food category "unknown plant material and others" in bear dietary content was positively related to the mean temperature of the warmest quarter, the seasonality of the precipitation (*Clim_15*) and to

mean diurnal temperature range, while it correlated negatively with the precipitation of the wettest month and to annual temperature range (*Clim_7*; Supplementary Tables 31-50).

Among land use variables, the percentage of broadleaf forests (*LC_3*), of bare areas (*LC_9*) and of sparse vegetation (*LC_*7) were the most important in shaping the brown bear dietary energy content. In turn, in areas where bare areas were common, bears ingested more invertebrates but less reproductive plant parts. The proportion of rangeland (*LC_8*) had the most significant impact on the brown bear dietary content; it related positively to the share of vertebrates and vegetative plant material, as well as the overall dietary diversity. Cultivated areas and pastures (*LC_2*) negatively affected the consumption of vertebrates and vegetative plant material. The urban areas (*LC_1*) affected only, and tentatively, the percentage of vegetative plant parts in the bear diet. The proportion of the food category "unknown plant material and others" in bear diet related positively to the percentage of natural landscape (*Nat. Landscape*), urban areas (*LC_1*) and bare habitats (*LC_9*), and negatively to broadleaf forest (*LC_3*) and rangeland (*LC_8*;

Moreover, bears had a more diverse dietary content in areas with lower mean diurnal temperature range (*Clim_2*), and a less cover of broadleaved forest (*LC_3*). The diet diversity was, however, positively related to the percentage of rangeland areas (*LC_8*). In areas where broadleaf forests had higher contribution to land cover, bears displayed a more diverse diet (Supplementary Tables 31-50).

Supplementary Tables

Supplementary Table 6. Name of climatic variables and definition.

Name	Definition
Clim_1	Annual mean temperature [1/10°C]
Clim_2	Mean diurnal range [1/10°C]
Clim_3	Isothermality * 100
Clim_4	Temperature seasonality * 100
Clim_5	Maximum Temperature of warmest month [1/10°C]
Clim_6	Minimum Temperature of coldest month [1/10°C]
Clim_7	Temperature Annual Range [1/10°C]
Clim_8	Mean Temperature of wettest quarter [1/10°C]
Clim_9	Mean Temperature of driest quarter [1/10°C]
Clim_10	Mean Temperature of warmest quarter [1/10°C]
Clim_11	Mean Temperature of coldest quarter [1/10°C]
Clim_12	Annual precipitation amount [mm]
Clim_13	Precipitation of wettest month [mm]
Clim_14	Precipitation of driest month [mm]
Clim_15	Precipitation Seasonality [coefficient of variation * 100]
Clim_16	Precipitation of wettest quarter [mm]
Clim_17	Precipitation of driest quarter [mm]
Clim_18	Precipitation of warmest quarter [mm]
Clim_19	Precipitation of coldest quarter [mm]

Supplementary Table 7. Name of land cover variables, definition and original categories.

Name	Definition	ID CCI-LC classification included
LC_1	Percentage of urban areas	1, 190
LC_2	Percentage of cultivated and pasture areas	2, 230,231, 232, 3
LC_3	Percentage of broadleaved forest	50, 60, 61, 62
LC_4	Percentage of needle-leaved forest	70, 71, 72, 80, 82, 90
LC_5	Percentage of Shrubland	100, 110, 120, 121, 122
LC_7	Sparse vegetation	150, 152, 153
LC_8	Percentage of rangeland areas	4
LC_9	Percentage of bare areas	200, 201, 202
Nat. Landscape	Percentage of natural areas at landscape scale. Value obtained from the application of a gaussian smoothing kernel function moving window of 11 x 11 km to a layer of percentage of natural areas.	50, 60, 61, 62, 70, 71, 72, 80, 81, 82, 90, 100, 110, 120, 121, 122, 130, 140, 150, 152, 153, 160, 170, 180, 200, 201, 202

Supplementary Table 10. Number of brown bear occurrences (n = 3,226,206) and percentage by each type of data/source and group. We include the identifier of the research group (ID).

ID	Tracks and Observations	GPS telemetry	VHF telemetry	Genetic and hairtraps	Cameras	Mixed
1	13103	0	0	0	0	0
2	0	88617	0	0	0	0
3	0	13106	0	356	0	0
4	0	109414	0	0	0	0
5	0	0	0	1598	0	0
6	0	0	0	0	0	375
7	2654	38222	0	346	0	0
8	0	0	0	22247	0	0
9	0	0	0	0	0	1150
10	3918	108672	0	334	0	0
11	556	3737	473	0	0	0
12	2234	5583	5584	0	0	0
13	0	29933	0	0	0	0
14	47	0	0	0	0	0
15	1859	115397	0	0	0	0
16	201	0	0	0	130	0
17	103	0	0	0	0	0
18	114	0	0	0	0	0
19	0	1959497	0	0	0	0
20	0	0	0	0	0	2094
21	2318	0	0	0	0	0
22	0	1309	0	0	0	0
23	0	183133	0	0	0	0
24	1736	0	10165	0	0	0
25	0	165165	0	0	0	0
26	0	0	700	0	0	0
27	270	36728	0	0	0	0
28	6	1438	0	0	91	0
29	2861	124561	0	0	0	0
30	640	0	0	0	0	0
31	91	0	0	0	0	0
32	2408	0	0	0	0	0
33	401	0	0	0	0	0
34	0	0	0	0	106	0
35	598	0	0	0	0	0
36	0	159827	0	0	0	0
TOTAL	36118	3144339	16922	24881	327	1151
Percentage	1.12	97.46	0.52	0.77	0.01	0.11

Supplementary Table 11. Number of pixels of 1x1 km with presence of brown bear by subpopulation (n presences all systems), the number of pixels in terrestrial systems (n presences in terrestrial), the number of presences used to fit the brown bear models at habitat scale (including models comparing biotic variables and brown bear model at habitat scale; n presences selected to train the habitat models), the number of presences used to validate the brown bear models at habitat scale (brown bear model at habitat scale; n presences selected to validate the habitat model).

**Subpopulation**	**n presences all systems**	**n presences in terrestrial**	**n presences selected to train the habitat models**	**n presences selected to validate the habitat model**
Alpine	3091	3087	1600	400
Baltic	2449	2447	1600	400
Cantabrian	2194	2186	1600	400
Eastern Carpathian	8208	8202	1600	400
Western Carpathian	3191	3190	1600	400
Caucasian	2132	2132	1600	400
Apennine	1151	1148	918	230
East Balkan	2278	2278	1600	400
Pindus	3612	3603	1600	400
Dinaric	8954	8947	1600	400
Karelian	18490	1793	1600	400
Pyrenees	1289	1289	1031	258
Scandinavian	45119	44151	1600	400
Turkey	471	471	377	94

Supplementary Table 12. Evaluation of univariable models for no correlated bioclimatic variables at range scale using historical distribution and historical climate. We indicate with a grey shadow the best four models based on Akaike information criterion (AIC). These variables were used to model the habitat of brown bear in all models including climatic variables.

**Model/Variable**	**AIC**
Clim_2	32031.61
Clim_3	24660.51
Clim_4	26890.17
Clim_8	27219.84
Clim_9	23133.48
Clim_13	29054.25
Clim_14	33658.36
Clim_15	32727.45
Clim_18	29809.06
Clim_19	35855.12

Supplementary Table 13. Results for the brown bear species distribution model for the historical range, based on bayesian GLM predicting presence (using historical distribution in Eurasia) as a function of bioclimatic variables. We report model coefficients (best estimates and their SE), Monte Carlo standard error (MCSE), confident intervals (10%, 50% and 90%), number of effective sample size (Neff) and the potential scale reduction factor on split chains (Rhat; at convergece Rhat=1).

	**mean**	**mcse**	**sd**	**10%**	**50%**	**90%**	**Neff**	**Rhat**
Intercept	-9.55	0.01	0.772	-10.53	-9.55	-8.56	9631.00	1.00
Clim_3	18.10	0.03	2.854	14.49	18.07	21.75	8364.00	1.00
Clim_4	7.52	0.01	0.814	6.49	7.50	8.57	7706.00	1.00
Clim_8	0.62	0.00	0.121	0.46	0.62	0.78	9577.00	1.00
Clim_9	2.95	0.00	0.268	2.61	2.94	3.30	7431.00	1.00
Clim_3_c	-17.76	0.04	3.902	-22.81	-17.68	-12.85	7933.00	1.00
Clim_4_c	-2.57	0.00	0.329	-3.00	-2.56	-2.15	7501.00	1.00
Clim_8_c	-0.38	0.00	0.057	-0.45	-0.37	-0.30	9753.00	1.00
Clim_9_c	-1.10	0.00	0.085	-1.21	-1.09	-0.99	7544.00	1.00

Supplementary Table 14. Sample average posterior predictive distribution of the outcome for the for the brown bear species distribution model for the historical range. A plausible mean indicates when compared with the mean (y; y_{mean} = 0.3794) does not mean that it is a good model, but a not plausible mean indicates a wrong model.

	**mean**	**sd**	**10%**	**50%**	**90%**
mean_PPD	0.38	0.013	0.36	0.38	0.40

Supplementary Table 15. Estimates and standard error (SE) for the expected log pointwise predictive density (elpd_waic), the effective number of parameters (p_waic) and the information criterion waic (which is just -2 * elpd_waic, i.e., converted to deviance scale) for the brown bear species distribution model for the historical range. Diagnostics for Pareto smoothed importance sampling (PSIS) indicated that all pareto k estimates were good (k < 0.5).

	**Estimate**	**SE**
**elpd_WAIC**	-1030.0	19.7
**p_WAIC**	8.0	0.3
**WAIC**	2060.0	39.5

Supplementary Table 16. Estimates and standard error (SE) for the expected log pointwise predictive density (elpd_waic), the effective number of parameters (p_waic) and the information criterion waic (which is just -2 * elpd_waic, i.e., converted to deviance scale) for a null species distribution model at range scale, based on bayesian GLM predicting the distribution with only the intercept.

	**Estimate**	**SE**
**elpd_WAIC**	-1388.3	10.9
**p_WAIC**	1.0	0.0
**WAIC**	2776.5	21.8

Supplementary Table 18. Fitted bayesian models (BMs) explaining brown bear distribution. We show the name for each model (Model name), whether abiotic and/or biotic variables are included in the models (Predictors included) and the type of model, which indicate if it is a simple Bayesian model (with uninformative priors, BSM) or a hierarchical Bayesian model (where climatic variables are using priors based in the model of brown bear at range scale, BHM). In addition to the three BMs described in the main text: (1) a model with abiotic and biotic predictors, (2) a model with abiotic predictors only, and (3) a model with biotic predictors only, we calculated two other alternative models in order to evaluate the effect of combining different data. Those alternative models (shaded in grey) were alternatives to both BHMs, the model with abiotic and biotic predictors (SHM_{ABI}), and the model with abiotic predictors only (SHM_{AI}), the SHM_{ABC} and SHM_{AC} respectively which used the same predictors but were BSMs only using data from brown bear *Occurrence Database* (Methods).

**Model name**	**Predictors included**	**Type of model**
SHM_{ABI}	Abiotic and biotic	BHM
SHM_{ABC}	Abiotic and biotic	BSM
SHM_{AI}	Abiotic	BHM
SHM_{AC}	Abiotic	BSM
SHM_{BC}	Biotic	BSM
SHM_{Null}	Only the intercept	BSM

Supplementary Table 19. Correlation among land use variables with current data of brown bear.

	LC_1	***LC_2***	***LC_3***	***LC_4***	***LC_5***	***LC_8***	***LC_9***	***Nat. Landscape***
LC_1	1.00							
LC_2	0.05	1.00						
LC_3	-0.04	-0.27	1.00					
LC_4	-0.05	-0.37	-0.39	1.00				
LC_5	-0.02	-0.19	-0.13	-0.11	1.00			
LC_8	-0.01	0.00	-0.12	-0.18	-0.08	1.00		
LC_9	-0.01	-0.05	-0.07	-0.08	-0.01	-0.03	1.00	
Nat. Landscape	-0.08	**-0.71**	0.24	0.57	0.13	-0.30	0.01	1.00

Supplementary Table 20. Evaluation of univariable models for land use variables at using the *Ocurrence Database*. We indicate with a grey shadow the best four models based in Akaike information criterion (AIC). These variables were used to model the habitat of brown bear in all models including land use variables.

**Variable**	**AIC**	
LC_1	55156.95	
LC_2	54887.07	Correlated with *Nat. Landscape*
LC_3	55019.35	
LC_4	54640.32	
LC_5	55240.58	
LC_8	55223.67	
LC_9	55147.85	
Nat. Landscape	54342.63	
Null	55250.60	

Supplementary Table 21. Performance for univariable species distribution models (frequentist binomial GLMMs) fitted for each food category explaining the distribution of brown bear. For each food category (All species, Reproductive plants, Vegetative plants, Unknown plants, Invertebrate and Vertebrates), we fitted two models, one with *Biotic variables* and other using *Biotic_binary variables*. To select variables representing the biotic interaction for the brown bear model at habitat scale we excluded the group including all species as it was highly correlated with other biotic variables. We used 19,926 presences and 19,926 absences to fit these models (Supplementary Table 3). We show the Akaike information criterion (AIC; a lower AIC value indicates a better fit of the model) for each model, and for each diet category, we show the difference in AIC between both univariable models (AIC *Biotic_binary variables* – AIC *Biotic variables*; positive values in the difference indicate that *Biotic variables* models have a better fit than *Biotic_binary variables*) and the difference of the AIC *Biotic variables* with the best univariable model considering biotic information (Delta AIC *Biotic variables*). In addition we calculated a *Null* model (a GLMM with only the interaction term) as a reference and we report the AIC and delta of this model.

**Group of species**	**Variable name**		**AIC models**		**AIC *Biotic_binary variables* – AIC *Biotic variables***	**Delta AIC *Biotic variables***
	Biotic variables	***Biotic_binary variables***	***Biotic variables***	***Biotic_binary variables***		
All species	$Bio_{All\_species}$	$Bio\_binary_{All\_species}$	54594.33	55070.34	476.01	0.00
Reprod plant	Bio_{Reprod_plant}	$Bio\_binary_{Reprod\_plant}$	54604.92	54959.81	354.89	10.59
Veget plant	$Bio_{Unknown\_plant}$	$Bio\_binary_{Unknown\_plant}$	55238.03	55180.96	-57.08	643.70
Unknown plant	$Bio_{Unknown\_plant}$	$Bio\_binary_{Unknown\_plant}$	55029.78	55141.39	111.61	435.45
Invertebrates	$Bio_{Invertebrates}$	$Bio\_binary_{Invertebrates}$	55199.62	55252.57	52.95	605.28
Vertebrates	$Bio_{Vertebrates}$	$Bio\_binary_{Vertebrates}$	55165.40	55227.99	62.59	571.07
Null	-	-	55250.60		-	656.27

Supplementary Table 23. Results for the Bayesian hierarchical model using abiotic and biotic factors to explain brown bear distribution and the lower bound of biotic variables ($SHM_{ABI\ Lower\ Bound}$). We report model coefficients (best estimates and their SE), Monte Carlo standard error (MCSE), confident intervals (10%, 50% and 90%), number of effective sample size (Neff) and the potential scale reduction factor on split chains (Rhat; at convergence Rhat=1).

	**mean**	**mcse**	**sd**	**10%**	**50%**	**90%**	**Neff**	**Rhat**
Intercept	-4.28	0.02	0.550	-4.88	-4.25	-3.66	1194	1.002
Clim_3	22.57	0.02	1.749	20.35	22.56	24.81	10157	1.000
Clim_4	2.83	0.01	0.610	2.04	2.84	3.61	9106	1.000
Clim_8	1.06	0.00	0.054	1.00	1.06	1.13	10042	1.001
Clim_9	0.05	0.00	0.025	0.02	0.05	0.09	11262	1.000
Clim_3_c	-43.91	0.03	3.271	-48.10	-43.89	-39.77	10536	1.000
Clim_4_c	-1.42	0.00	0.346	-1.87	-1.43	-0.98	9376	1.000
Clim_8_c	-0.63	0.00	0.030	-0.67	-0.63	-0.59	10060	1.000
Clim_9_c	-0.19	0.00	0.015	-0.21	-0.19	-0.17	12237	1.000
LC_1	-0.08	0.00	0.018	-0.10	-0.08	-0.06	18451	1.000
LC_3	0.21	0.00	0.016	0.19	0.21	0.23	11411	1.000
LC_4	0.31	0.00	0.018	0.29	0.31	0.34	11270	1.000
Nat. Landscape	0.10	0.00	0.019	0.07	0.10	0.12	11505	1.000
Bio_{Reprod_plant}	0.16	0.00	0.016	0.14	0.16	0.18	16465	1.000
$Bio_{Invertebrates}$	-0.14	0.00	0.017	-0.17	-0.14	-0.12	18091	1.000
$Bio_{Unknown\_plant}$	0.25	0.00	0.037	0.21	0.25	0.30	13631	1.000
$Bio_{Vertebrates}$	0.09	0.00	0.014	0.07	0.09	0.11	16251	1.000

Supplementary Table 24. Results for the Bayesian hierarchical model using abiotic and biotic factors to explain brown bear distribution and the upper bound of biotic variables (*$SHM_{ABI\ Upper\ Bound}$*). We report model coefficients (best estimates and their SE), Monte Carlo standard error (MCSE), confident intervals (10%, 50% and 90%), number of effective sample size (Neff) and the potential scale reduction factor on split chains (Rhat; at convergence Rhat=1).

	**mean**	**mcse**	**sd**	**10%**	**50%**	**90%**	**Neff**	**Rhat**
Intercept	-4.27	0.01	0.514	-4.87	-4.26	-3.67	2475	1.001
Clim_3	22.56	0.02	1.726	20.36	22.54	24.77	10528	1.000
Clim_4	2.83	0.01	0.615	2.04	2.83	3.62	10305	1.000
Clim_8	1.06	0.00	0.055	0.99	1.06	1.13	9587	1.000
Clim_9	0.05	0.00	0.025	0.02	0.05	0.09	11147	1.000
Clim_3_c	-43.90	0.03	3.216	-48.05	-43.88	-39.81	10327	1.000
Clim_4_c	-1.42	0.00	0.349	-1.87	-1.43	-0.97	10551	1.000
Clim_8_c	-0.63	0.00	0.031	-0.67	-0.63	-0.59	9875	1.000
Clim_9_c	-0.19	0.00	0.015	-0.21	-0.19	-0.17	11174	1.000
LC_1	-0.08	0.00	0.018	-0.11	-0.08	-0.06	18058	1.000
LC_3	0.21	0.00	0.016	0.19	0.21	0.23	11074	1.000
LC_4	0.31	0.00	0.018	0.29	0.31	0.34	11058	1.000
Nat. Landscape	0.10	0.00	0.019	0.07	0.10	0.12	11774	1.000
Bio_{Reprod_plant}	0.16	0.00	0.017	0.14	0.16	0.18	15125	1.000
$Bio_{Invertebrates}$	-0.14	0.00	0.017	-0.17	-0.14	-0.12	17817	1.000
$Bio_{Unknown\_plant}$	0.25	0.00	0.037	0.21	0.25	0.30	13062	1.000
$Bio_{Vertebrates}$	0.09	0.00	0.014	0.07	0.09	0.11	15226	1.000

Supplementary Table 28. Sum of rates of Estimated Dietary Energy Content (*rEDEC*) described at the species level for each subpopulation ($rEDEC_{Subp}$).

Subpopulation	$rEDEC_{Subp}$
Alpine	0.33
Baltic	0.75
Cantabrian	0.96
Eastern Carpathian	0.77
Western Carpathian	0.80
Caucasian	0.20
Apennine	0.83
East Balkan	1.00
Pindus	0.51
Dinaric	0.81
Karelian	0.90
Pyrenees	0.50
Scandinavian	0.86
Turkey	0.63

Supplementary Table 29. Sum of rates of Estimated Dietary Energy Content, *rEDEC*, described at the species level for each subpopulation by diet group.

Subpopulation	Invertebr.	Reprod. plant material	Unknown plant material and others	Veget. plant material	Vertebr.
Apennine	0.29	87.46	0.00	1.49	10.75
Cantabrian	1.30	69.49	8.73	2.60	17.80
Scandinavian	3.35	39.97	0.34	5.18	51.16
Alpine	16.85	23.75	21.36	5.05	32.99
Eastern Carpathian	0.00	87.73	0.00	0.71	11.56
East Balkan	1.50	94.12	0.00	0.00	4.37
Dinaric	0.00	91.76	0.00	0.00	8.24
Turkey	0.00	17.55	67.18	0.00	15.27
Pyrenees	0.00	51.65	5.87	4.04	38.44
Pindos	0.00	86.86	0.00	1.25	11.89
Baltic	0.53	88.42	0.04	0.38	10.63
Karelian	6.37	56.69	0.00	0.00	36.94
Western Carpathian	0.60	80.10	0.00	0.00	19.29
Caucasian	0.00	32.15	0.00	0.00	67.85

Supplementary Table 30. Rates (from the total described at the species level for each subpopulation) of Estimated Dietary Energy Content, *rEDEC*, by origin (Wild/Human) for each subpopulation.

Subpopulation	Wild	Human
Apennine	76.58	21.67
Cantabrian	83.20	16.70
Scandinavian	61.66	38.34
Alpine	85.05	14.95
Eastern Carpathian	47.48	52.52
East Balkan	7.02	92.98
Dinaric	43.57	56.43
Turkey	94.40	5.60
Pyrenees	79.89	20.11
Pindos	59.21	40.72
Baltic	41.03	58.97
Karelian	97.64	2.36
Western Carpathian	57.23	42.77
Caucasian	49.24	50.76

Supplementary Table 51. Statistics, minimum, median, mean and maximum of the evaluations of species distribution models for wild food species.

	TSS	Sensitivity	Specificity
Minimum	0.11	36.23	44.33
Median	0.47	77.88	69.37
Mean	0.47	78.04	69.43
Maximum	0.74	97.47	91.27

Supplementary Table 53. Average importance for the variables used to fit species distribution models for wild food species.

Variable	Importance
Clim_1	0.32
Clim_12	0.12
Clim_15	0.12
Clim_7	0.24
LC_2	0.25
LC_3	0.04
LC_4	0.14
LC_5	0.03
LC_7	0.01
LC_8	0.00
LC_9	0.00

Supplementary Table 55. Mean habitat suitability change (in percentage) for each food category and scenario.

Scenario	Diet category	Habitat suitability change (%)
SSP1-2.6	reproductive_plant_material	-42.87
SSP1-2.6	unknown_plant_material_and_others	-31.83
SSP1-2.6	vegetative_plant_material	-32.44
SSP1-2.6	invertebrates	18.44
SSP1-2.6	vertebrates	-46.94
SSP3-6.0	reproductive_plant_material	-45.72
SSP3-6.0	unknown_plant_material_and_others	-34.20
SSP3-6.0	vegetative_plant_material	-36.32
SSP3-6.0	invertebrates	25.77
SSP3-6.0	vertebrates	-44.65
SSP5-8.5	reproductive_plant_material	-62.08
SSP5-8.5	unknown_plant_material_and_others	-53.49
SSP5-8.5	vegetative_plant_material	-38.03
SSP5-8.5	invertebrates	8.21
SSP5-8.5	vertebrates	-52.06

Supplementary Table 57. Performance for bayesian models (BMs) explaining brown bear distribution. For each model we show the model name (See Supplementary Table 18), estimates, standard error (SE) the information criterion WAIC (which is just -2 * elpd_waic, i.e., converted to deviance scale), the expected log pointwise predictive density (elpd_waic), the effective number of parameters (p_waic), and for the mean of the sample average posterior predictive distribution of the outcome (mean_PPD); and delta WAIC values. A plausible mean indicates when compared with the mean (y; y_{mean} = 0.5) does not mean that it is a good model, but a not plausible mean indicates a wrong model. Diagnostics for Pareto smoothed importance sampling (PSIS) indicated that all pareto k estimates were good (k < 0.5). We used 19,926 presences and 19,926 absences to fit these models (Supplementary Table 11). Alternative models are shaded in grey (See Supplementary Table 18).

	WAIC			elpd_WAIC		p_WAIC		mean_PPD	
**Model name**	**Estimate**	**SE**	**Delta WAIC**	**Estimate**	**SE**	**Estimate**	**SE**	**Estimate**	**SD**
SHM_{ABI}	52675.0	93.1	0.0	-26337.5	46.6	26.7	0.3	0.50	0.003
SHM_{ABC}	52766.0	95.5	91.0	-26383.0	47.7	28.1	0.3	0.50	0.003
SHM_{AI}	53015.0	86.1	340.0	-26507.5	43.0	22.9	0.3	0.50	0.003
SHM_{AC}	53100.9	89.0	425.9	-26550.5	44.5	24.2	0.3	0.50	0.003
SHM_{BC}	54354.9	59.1	1679.9	-27177.4	29.5	18.2	0.1	0.50	0.003
SHM_{Null}	55250.6	0.0	2539.7	-27625.3	0.0	2.0	0.0	0.50	0.004

Supplementary Table 58. Results for the Bayesian hierarchical model using abiotic and biotic factors to explain brown bear distribution (SHM_{ABI}; see Supplementary Table 18). We report model coefficients (best estimates and their SE), Monte Carlo standard error (MCSE), confident intervals (10%, 50% and 90%), number of effective sample size (Neff) and the potential scale reduction factor on split chains (Rhat; at convergence Rhat=1).

	**mean**	**mcse**	**sd**	**10%**	**50%**	**90%**	**Neff**	**Rhat**
Intercept	-15.51	0.02	0.919	-16.72	-15.49	-14.35	3619	1.000
Clim_3	18.85	0.03	1.655	16.70	18.89	20.95	3786	0.999
Clim_4	6.01	0.01	0.504	5.36	6.01	6.65	3838	1.000
Clim_8	0.96	0.00	0.053	0.89	0.96	1.03	3527	1.001
Clim_9	3.34	0.00	0.172	3.12	3.34	3.56	3207	1.000
Clim_3_c	-38.03	0.05	2.984	-41.82	-38.05	-34.23	3725	0.999
Clim_4_c	-3.18	0.00	0.275	-3.53	-3.18	-2.84	3808	1.000
Clim_8_c	-0.50	0.00	0.030	-0.54	-0.50	-0.46	3661	1.000
Clim_9_c	-1.16	0.00	0.051	-1.22	-1.16	-1.09	3118	1.000
LC_1	-0.06	0.00	0.016	-0.08	-0.06	-0.04	6188	0.999
LC_3	0.20	0.00	0.017	0.18	0.20	0.22	3701	0.999
LC_4	0.28	0.00	0.019	0.25	0.28	0.30	3836	1.000
Nat. Landscape	0.09	0.00	0.019	0.07	0.09	0.11	3949	1.000
Bio_{Reprod_plant}	0.18	0.00	0.016	0.15	0.18	0.20	5758	1.000
$Bio_{Invertebrates}$	-0.14	0.00	0.017	-0.17	-0.14	-0.12	6011	0.999
$Bio_{Unknown\_plant}$	0.22	0.00	0.038	0.17	0.22	0.27	4489	1.000
$Bio_{Vertebrates}$	0.09	0.00	0.014	0.08	0.09	0.11	5715	1.000

Supplementary Table 59. Results for the simple Bayesian model (no hierarchical) using abiotic and biotic factors to explain brown bear distribution (*SHM_{ABC}*; see Supplementary Table 18). We report model coefficients (best estimates and their SE), Monte Carlo standard error (MCSE), confident intervals (10%, 50% and 90%), number of effective sample size (Neff) and the potential scale reduction factor on split chains (Rhat; at convergence Rhat=1).

	**mean**	**mcse**	**sd**	**10%**	**50%**	**90%**	**Neff**	**Rhat**
Intercept	-6.28	0.02	0.768	-7.26	-6.28	-5.30	2498	1.001
Clim_3	4.17	0.02	1.327	2.45	4.18	5.86	3155	1.001
Clim_4	5.54	0.03	1.562	3.55	5.54	7.56	2312	1.000
Clim_8	1.17	0.00	0.071	1.08	1.17	1.26	3743	1.000
Clim_9	4.42	0.01	0.358	3.96	4.42	4.89	2952	1.001
Clim_3_c	-9.04	0.04	2.185	-11.87	-8.99	-6.32	3536	1.000
Clim_4_c	-3.10	0.02	0.908	-4.27	-3.10	-1.94	2411	0.999
Clim_8_c	-0.62	0.00	0.040	-0.67	-0.62	-0.57	3662	1.000
Clim_9_c	-1.47	0.00	0.107	-1.60	-1.47	-1.33	2959	1.000
LC_1	-0.06	0.00	0.016	-0.08	-0.06	-0.04	5557	1.000
LC_3	0.19	0.00	0.016	0.17	0.19	0.21	4279	1.000
LC_4	0.26	0.00	0.018	0.24	0.26	0.28	3755	1.001
Nat. Landscape	0.10	0.00	0.018	0.07	0.10	0.12	4202	1.000
Bio_{Reprod_plant}	0.17	0.00	0.017	0.15	0.17	0.19	5231	1.000
$Bio_{Invertebrates}$	-0.15	0.00	0.017	-0.17	-0.15	-0.13	5790	1.000
$Bio_{Unknown\_plant}$	0.18	0.00	0.038	0.14	0.18	0.23	3451	1.000
$Bio_{Vertebrates}$	0.10	0.00	0.014	0.08	0.10	0.12	5635	1.000

Supplementary Table 60. Results for the Bayesian hierarchical model using abiotic factors to explain brown bear distribution (*SHM_{AI}*; see Supplementary Table 18). We report model coefficients (best estimates and their SE), Monte Carlo standard error (MCSE), confident intervals (10%, 50% and 90%), number of effective sample size (Neff) and the potential scale reduction factor on split chains (Rhat; at convergence Rhat=1).

	**mean**	**mcse**	**sd**	**10%**	**50%**	**90%**	**Neff**	**Rhat**
Intercept	-3.99	0.03	0.784	-4.95	-3.85	-3.21	608	1.008
Clim_3	21.76	0.02	1.735	19.52	21.77	24.00	8695	1.000
Clim_4	2.69	0.01	0.605	1.92	2.69	3.47	8625	1.000
Clim_8	1.04	0.00	0.053	0.97	1.04	1.11	9707	1.000
Clim_9	0.08	0.00	0.025	0.05	0.08	0.11	9904	1.000
Clim_3_c	-44.81	0.04	3.326	-49.15	-44.80	-40.53	7847	1.000
Clim_4_c	-1.65	0.00	0.345	-2.10	-1.65	-1.22	8835	1.000
Clim_8_c	-0.64	0.00	0.030	-0.67	-0.64	-0.60	9616	1.000
Clim_9_c	-0.22	0.00	0.014	-0.24	-0.22	-0.20	10895	1.000
LC_1	-0.09	0.00	0.019	-0.11	-0.09	-0.06	15879	1.000
LC_3	0.24	0.00	0.015	0.22	0.24	0.25	9899	1.000
LC_4	0.33	0.00	0.018	0.31	0.33	0.35	10101	1.000
Nat. Landscape	0.14	0.00	0.019	0.11	0.14	0.16	10237	1.000

Supplementary Table 61. Results for the simple Bayesian model using abiotic factors to explain brown bear distribution (SHM_{AC}; see Supplementary Table 18). We report model coefficients (best estimates and their SE), Monte Carlo standard error (MCSE), confident intervals (10%, 50% and 90%), number of effective sample size (Neff) and the potential scale reduction factor on split chains (Rhat; at convergence Rhat=1).

	**mean**	**mcse**	**sd**	**10%**	**50%**	**90%**	**Neff**	**Rhat**
Intercept	-2.30	0.01	0.710	-3.21	-2.30	-1.40	8627	1.001
Clim_3	4.27	0.01	1.333	2.57	4.28	5.97	9724	1.000
Clim_4	4.65	0.02	1.550	2.67	4.63	6.67	6879	1.001
Clim_8	1.35	0.00	0.072	1.25	1.35	1.44	8639	1.000
Clim_9	0.21	0.00	0.035	0.17	0.21	0.26	9993	1.000
Clim_3_c	-9.19	0.02	2.162	-11.96	-9.17	-6.45	11220	1.000
Clim_4_c	-2.82	0.01	0.904	-3.99	-2.81	-1.67	6939	1.001
Clim_8_c	-0.79	0.00	0.041	-0.85	-0.79	-0.74	8756	1.000
Clim_9_c	-0.29	0.00	0.021	-0.32	-0.29	-0.26	9983	1.000
LC_1	-0.09	0.00	0.018	-0.11	-0.09	-0.06	14894	1.000
LC_3	0.23	0.00	0.015	0.21	0.23	0.25	10046	1.000
LC_4	0.33	0.00	0.018	0.30	0.33	0.35	9824	1.000
Nat. Landscape	0.14	0.00	0.019	0.12	0.14	0.16	9838	1.000

Supplementary Table 62. Results for the simple Bayesian model using biotic factors to explain brown bear distribution (*SHM_{BC}*; see Supplementary Table 18). We report model coefficients (best estimates and their SE), Monte Carlo standard error (MCSE), confident intervals (10%, 50% and 90%), number of effective sample size (Neff) and the potential scale reduction factor on split chains (Rhat; at convergence Rhat=1).

	**mean**	**mcse**	**sd**	**10%**	**50%**	**90%**	**Neff**	**Rhat**
Intercept	0.01	0.00	0.172	-0.21	0.01	0.22	1514	1.000
Bio_{Reprod_plant}	0.31	0.00	0.016	0.29	0.31	0.33	8576	1.000
$Bio_{Invertebrates}$	-0.19	0.00	0.017	-0.21	-0.19	-0.17	9234	1.000
$Bio_{Unknown\_plant}$	0.37	0.00	0.038	0.32	0.37	0.42	7257	1.000
$Bio_{Vertebrates}$	0.05	0.00	0.014	0.03	0.05	0.07	8534	1.000

Supplementary Table 68. Results for the validation of the best Bayesian model (BM) explaining brown bear distribution, the Bayesian hierarchical model using abiotic and biotic factors (SHM_{ABI}; see Supplementary Table 18). to validate the model. We show the values to correctly classify the pseudo-absences of brown bear (true negative rate; TNR), to correctly classify the presences of brown bear (true positive rate; TPR) and classification accuracy (Acc.) at European scale and by subpopulation. We used an independent subset of data from the *Ocurrence Database*, the validation subset, which contains 4,982 presences and 4,982 absences to validate these models (Supplementary Table 11).

	**Europe**	**Alpine**	**Baltic**	**Cantabrian**	**Eastern Carpathian**	**Western Carpathian**	**Caucasian**	**Apennine**	**East Balkan**	**Pindus**	**Dinaric**	**Karelian**	**Pyrenees**	**Scandinavian**	**Turkey**
**TNR**	0.21	0.24	0.16	0.27	0.33	0.12	0.24	0.05	0.25	0.26	0.24	0.04	0.41	0.15	0.43
**TPR**	0.90	0.92	0.93	0.92	0.82	0.95	0.91	0.98	0.89	0.83	0.91	0.99	0.79	0.90	0.73
**Acc.**	0.56	0.58	0.54	0.59	0.57	0.53	0.58	0.52	0.57	0.54	0.57	0.51	0.60	0.53	0.58

Supplementary Figures

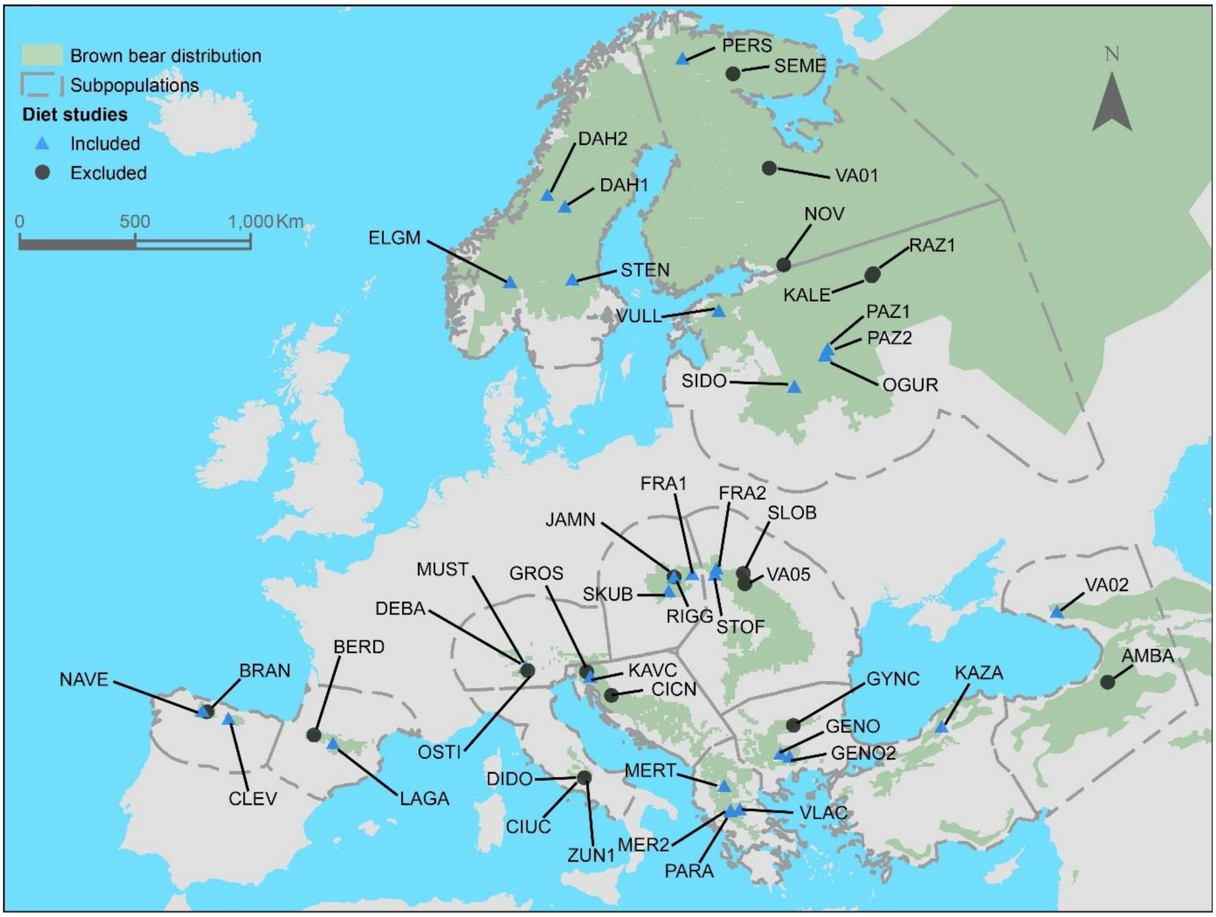

Supplementary Figure 1. Map showing the location of 47 studies of brown bear diet found in the review. For consistency with climate and land use data, we selected 31 studies conducted between 1989 and 2018 which had sufficient taxonomic resolution (genus and/or species; Supplementary Table 1).

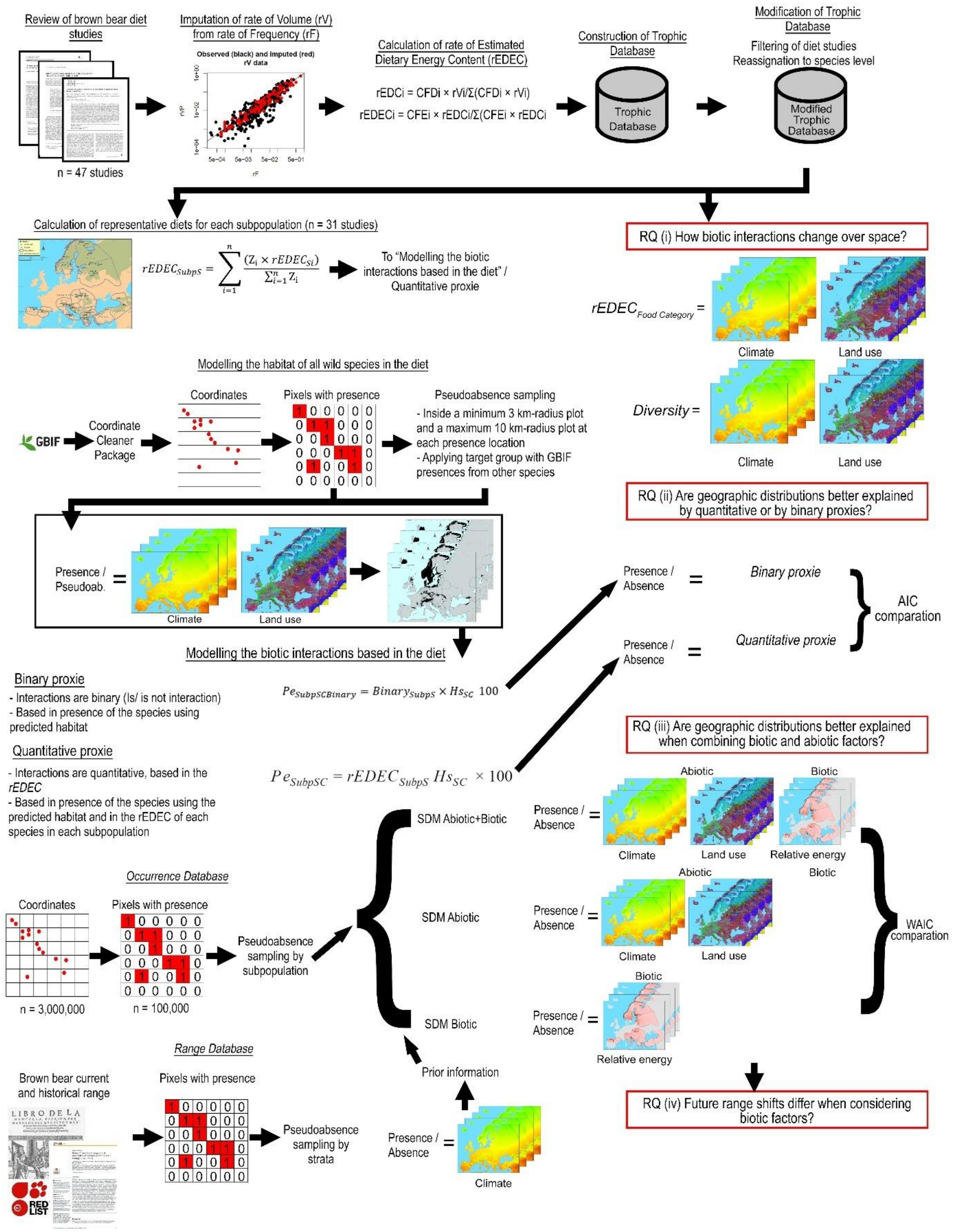

Supplementary Figure 2. Diagram showing the methodology with the process of data collection, cleaning and the analysis link to each specific research question.

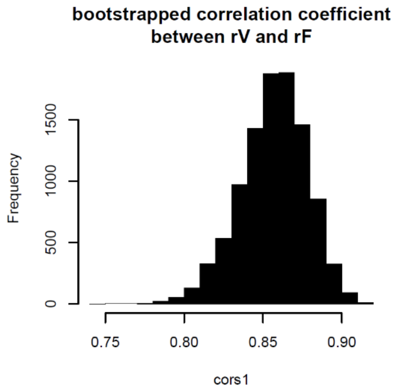

Supplementary Figure 3. Bootstrapped correlation between relative frequency and relative volume.

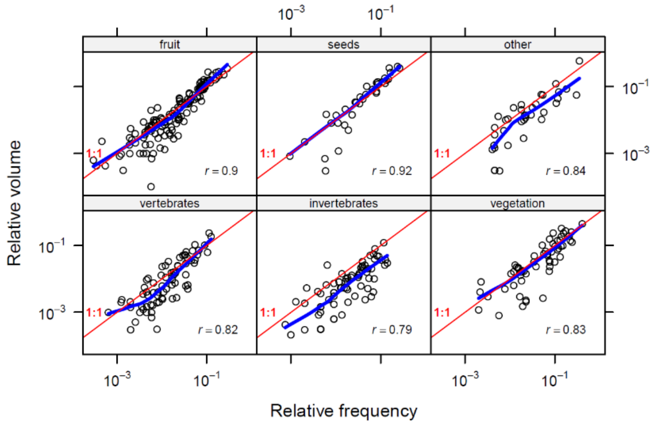

Supplementary Figure 4. Empirical relationships between relative frequency and relative volume.

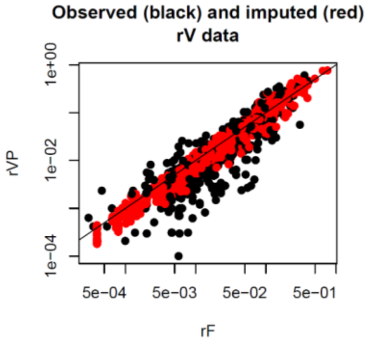

Supplementary Figure 5. Relationships between relative volume with relative frequency of observed and imputed data.

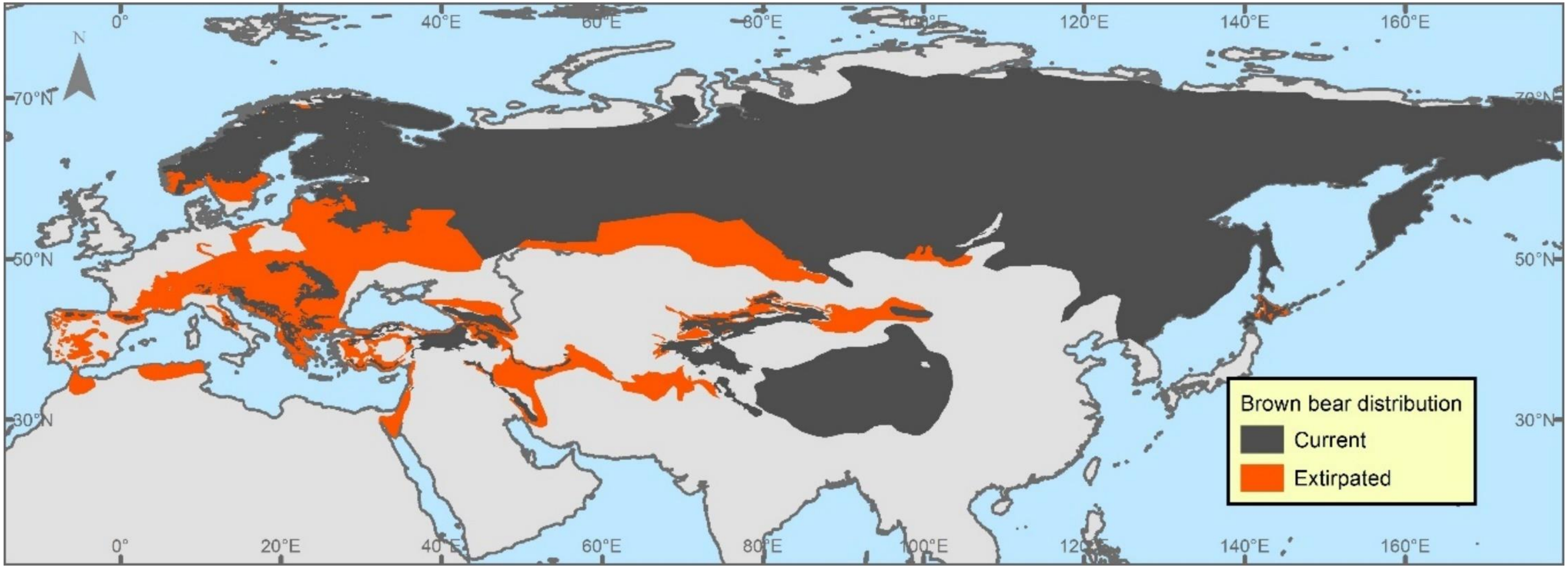

Supplementary Figure 6. Historical distribution (Current and Extirpated areas) of brown bear in Eurasia used in the Species Distribution Model range. Current distribution was obtained from the IUCN Red List spatial data and extirpated distribution was build based on IUCN data in its majority and completed with other sources (See methods section).

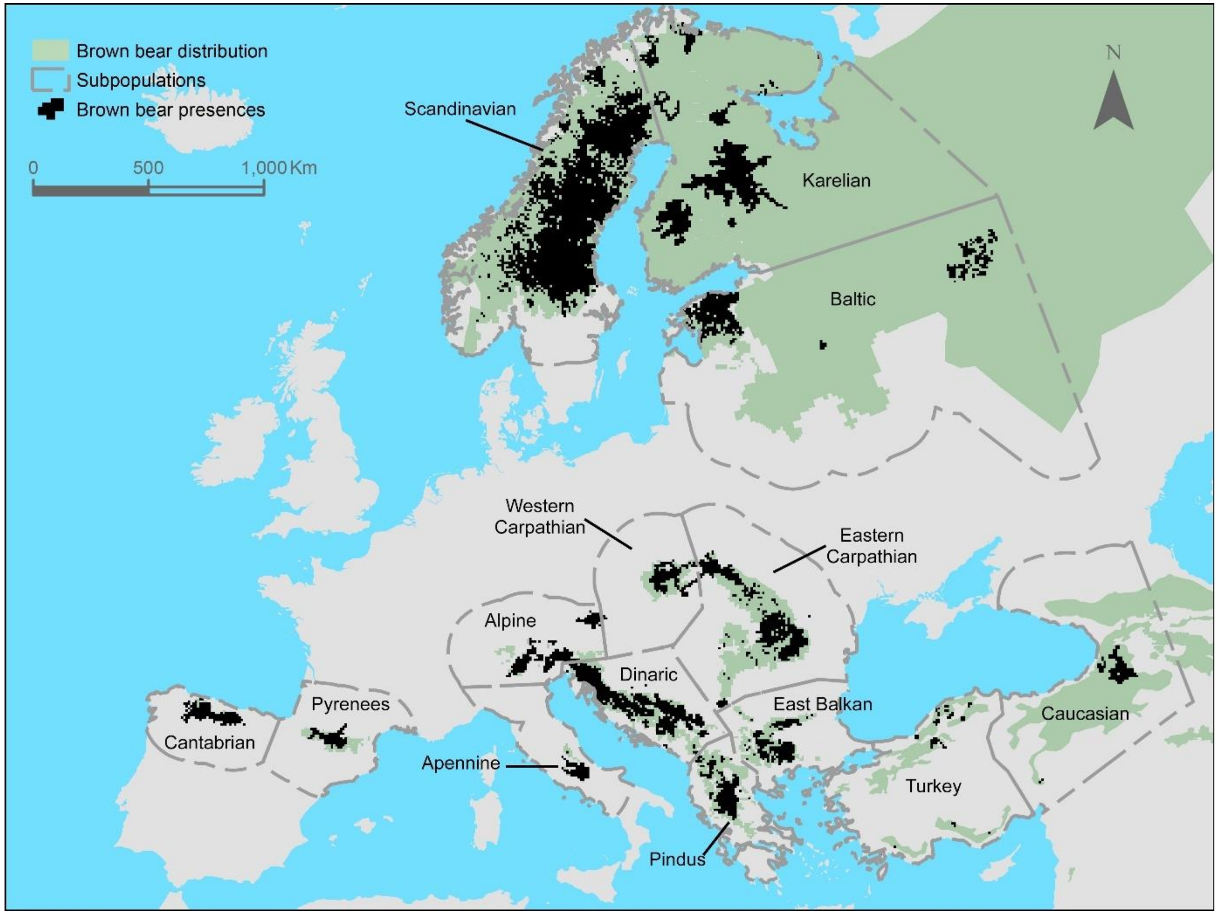

Supplementary Figure 7. Map showing 10×10 km cells with presence of brown bear from the *Occurrence Database* which includes brown bear presences at high resolution (1×1km).

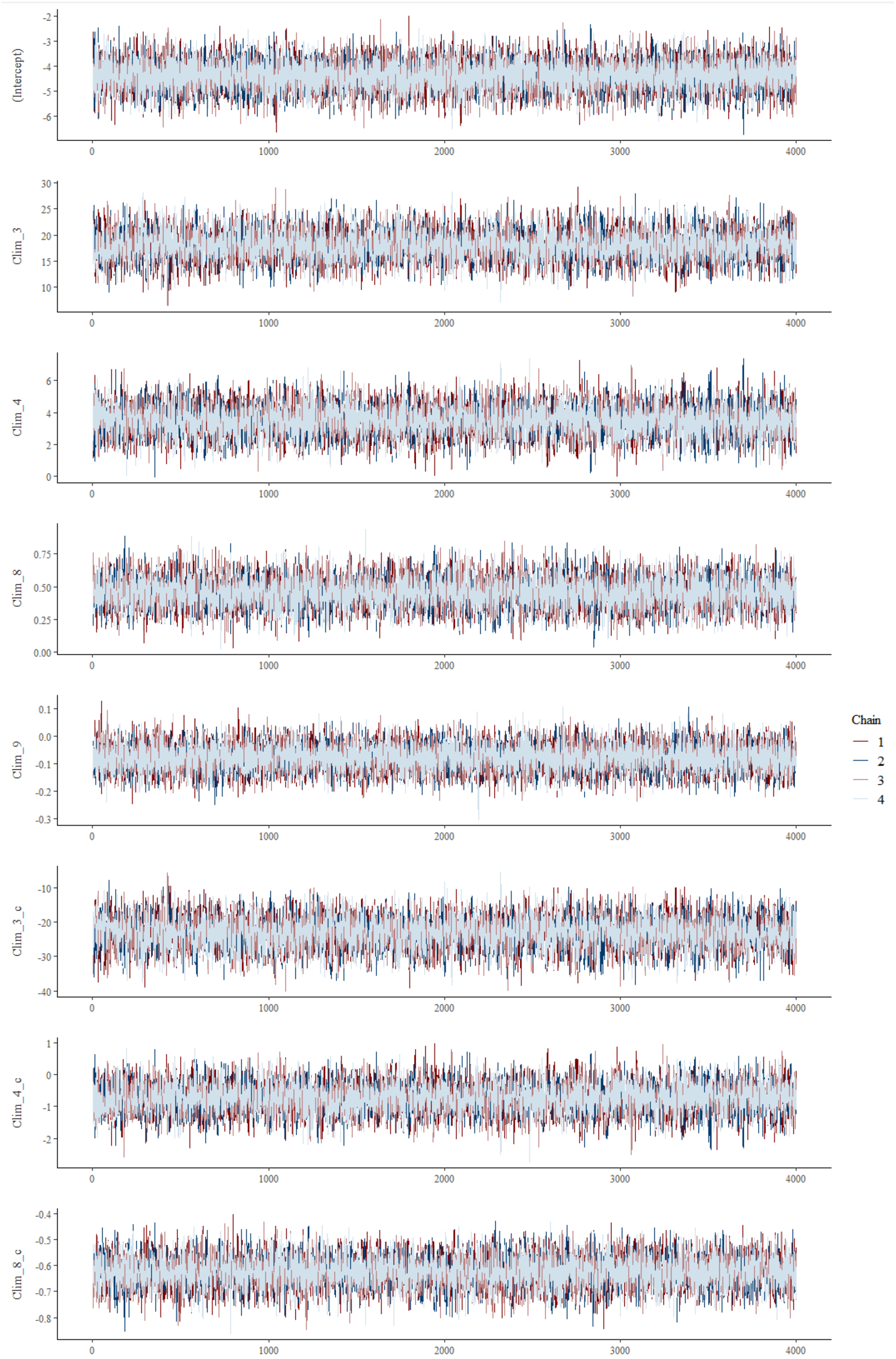

Supplementary Figure 8. Chains for the Species Distribution Model at range scale, the SDM_{Range}

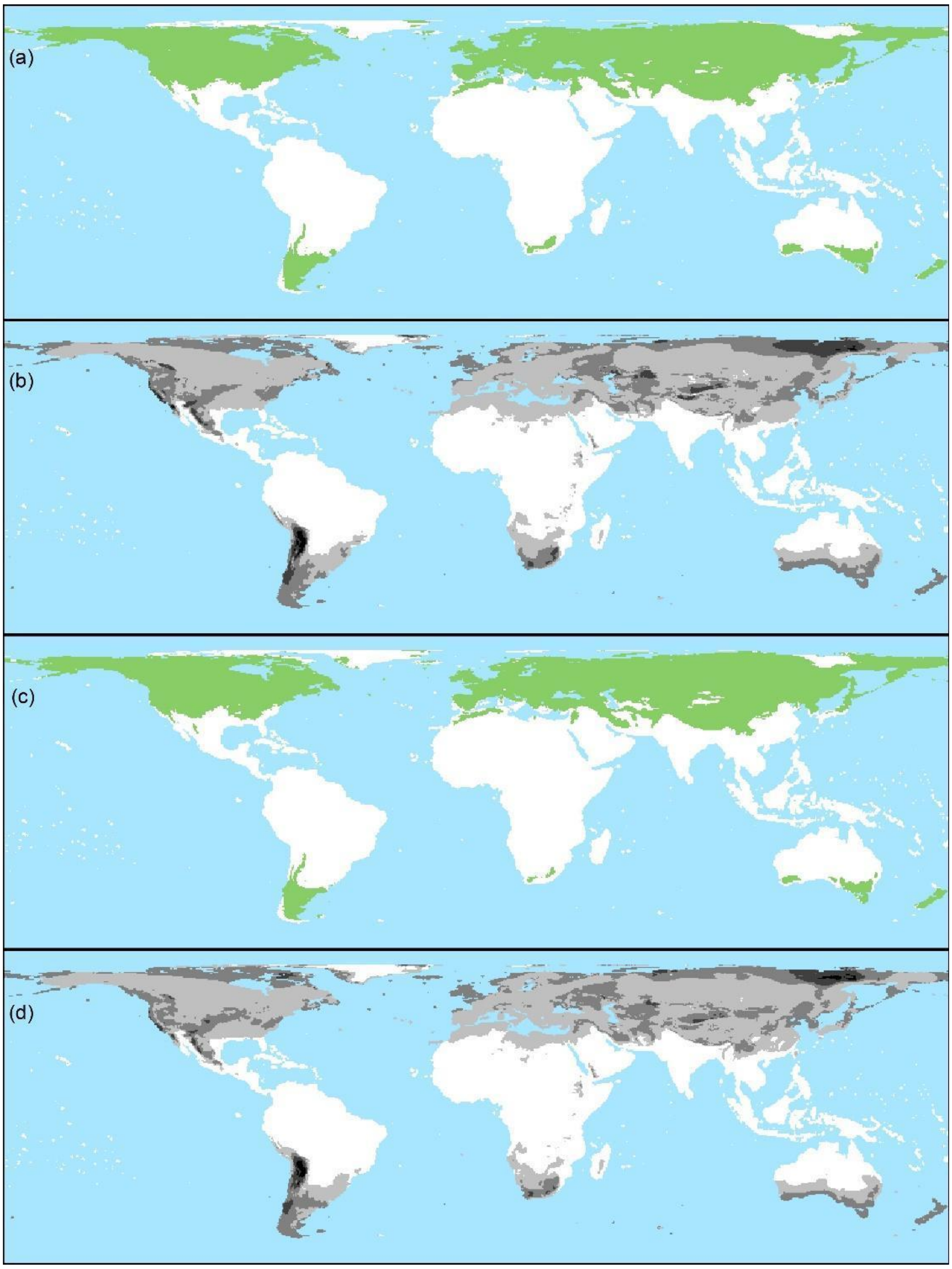

Supplementary Figure 9. Maps for the Bayesian model at range scale explaining brown bear distribution. We show the predicted distribution (a), and uncertainty in mean predicted (b) for historical climate data. And the predicted distribution (c), and uncertainty in mean predicted (d) for current climate data.

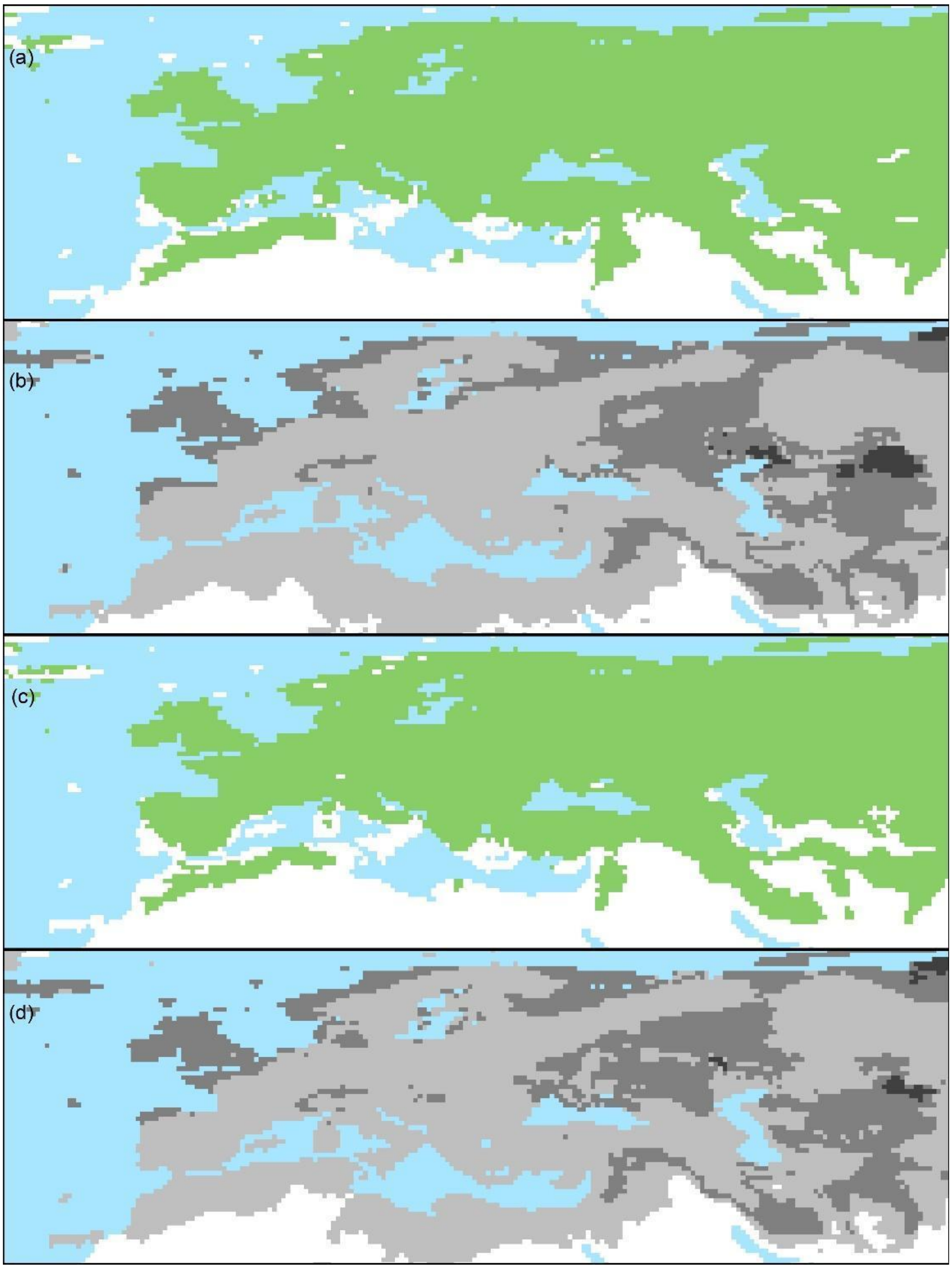

Supplementary Figure 10. Maps for the Bayesian model at range scale explaining brown bear distribution. We show the predicted distribution (a), and uncertainty in mean predicted (b) for historical climate data. And the predicted distribution (c), and uncertainty in mean predicted (d) for current climate data.

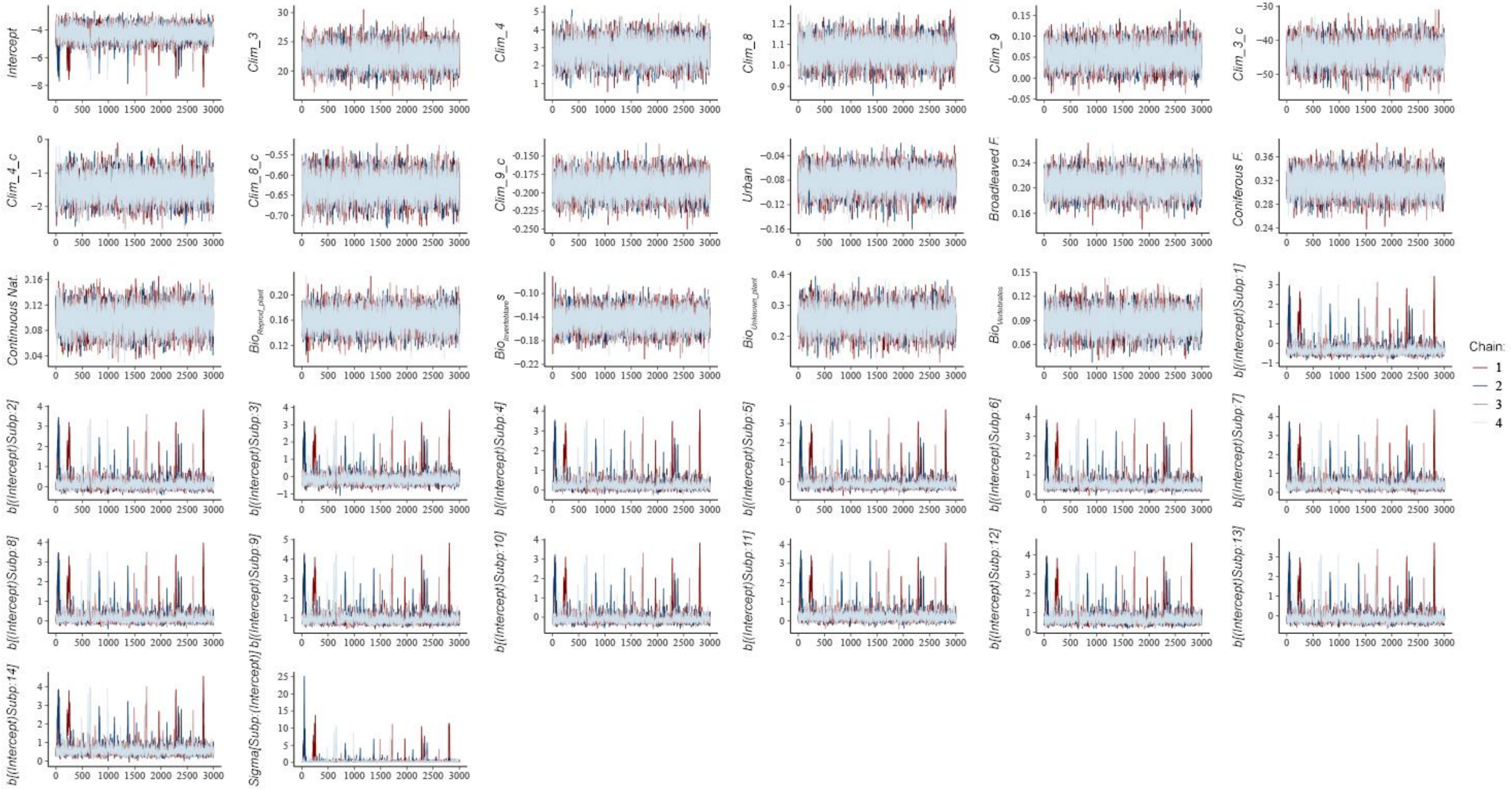

Supplementary Figure 11. Chains for the Bayesian model of brown bear habitat with abiotic and biotic factors combining current and historical data and using the lower bound (95% C.I.) from the biotic variables to assess the error propagation.

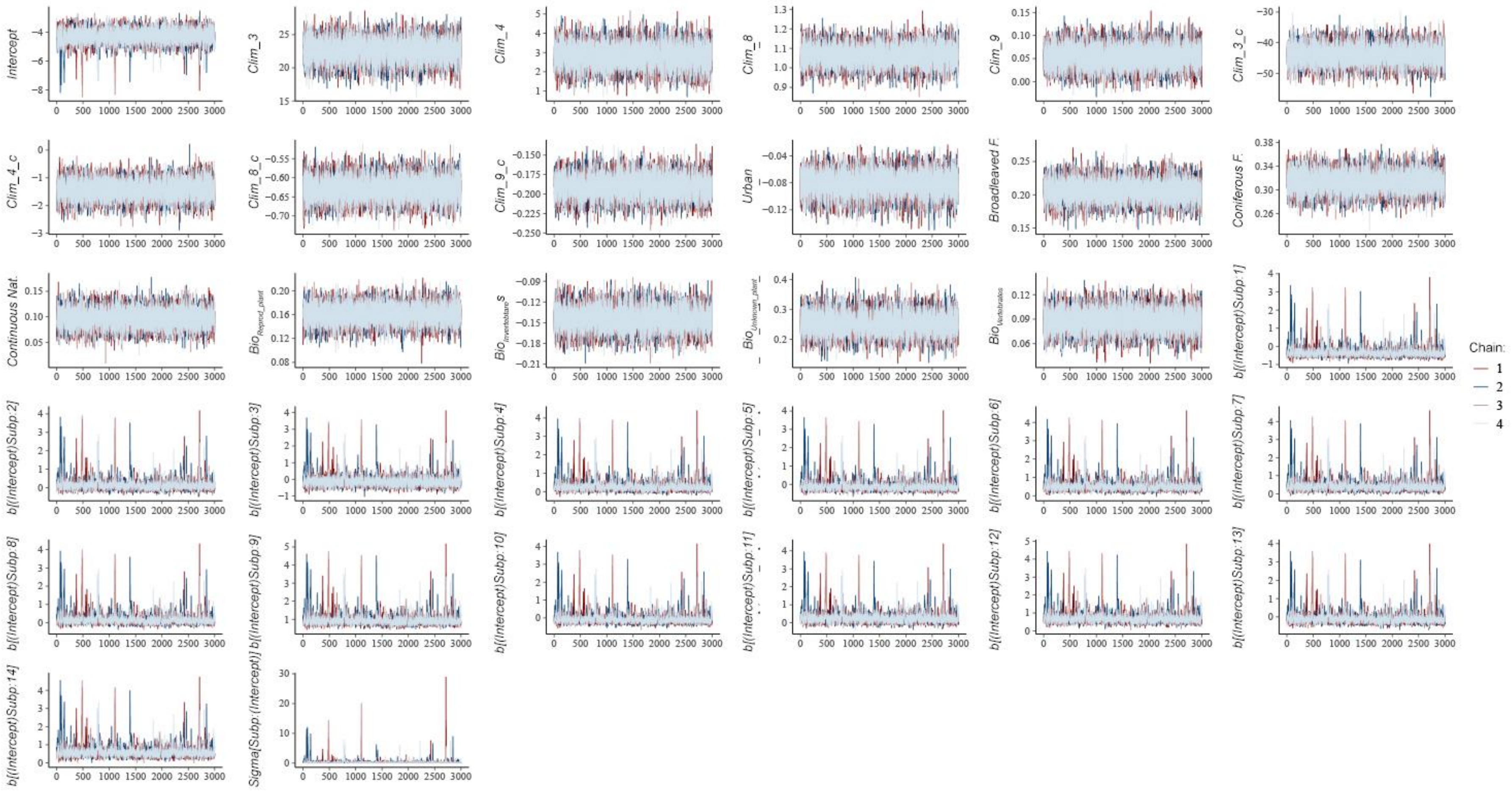

Supplementary Figure 12. Chains for the Bayesian model of brown bear habitat with abiotic and biotic factors combining current and historical data and using the upper bound (95% C.I.) from the biotic variables to assess the error propagation.

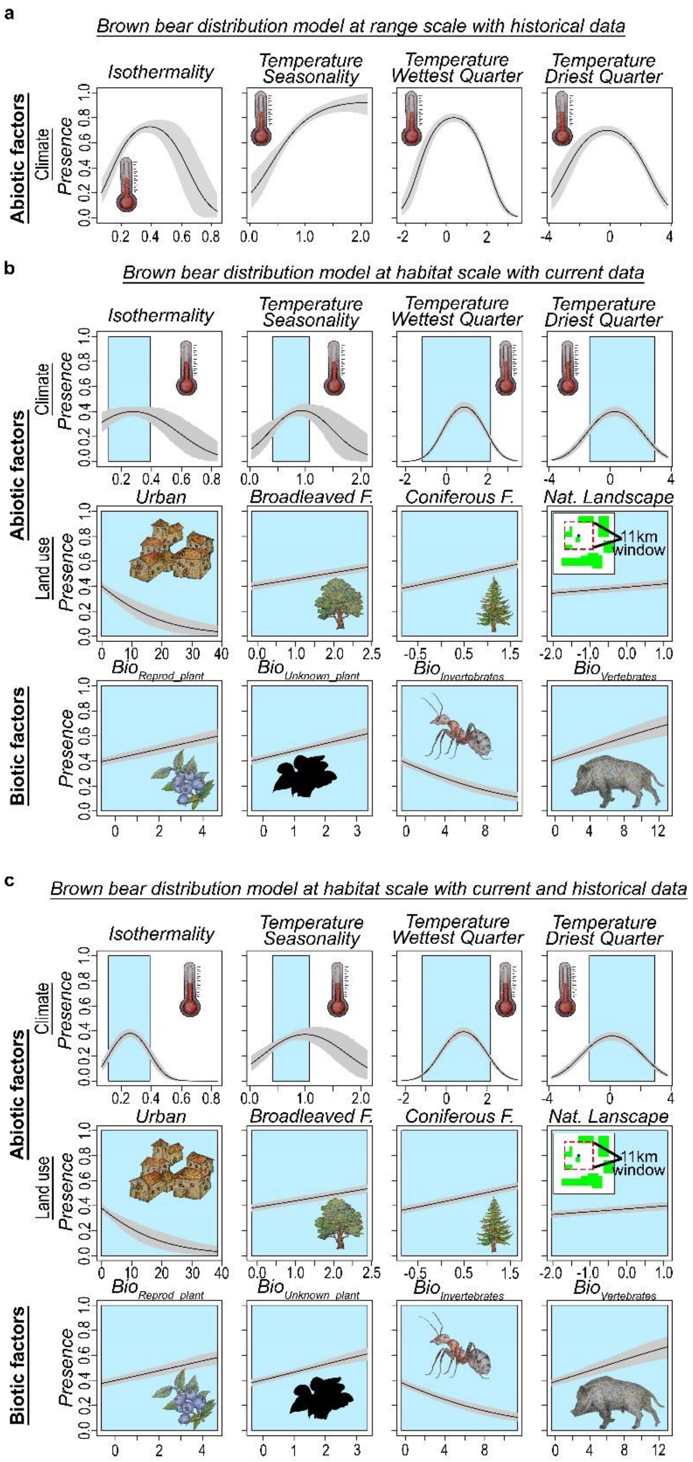

Supplementary Figure 13. Response plot of the three Bayesian models explaining the distribution of the brown bear. **a** Bayesian model at range scale using the historical distribution of brown bear and historical climate variables as predictors. **b** Simple Bayesian model (no hierarchical) using abiotic and biotic factors to explain brown bear distribution. **c** Distribution model for brown bear including both

abiotic and biotic factors was fitted combining both historical (*Range Database*) and current data (*Occurrence Database*). The continuous line represents the mean response value, and the grey area shows the model uncertainty (95% confidence interval). The blue area indicates the range of values of the current data. Isothermality (*Clim_3*), temperature seasonality (*Clim_4*), mean temperature of the wettest quarter (*Clim_8*) and mean temperature of the driest quarter (*Clim_9*).

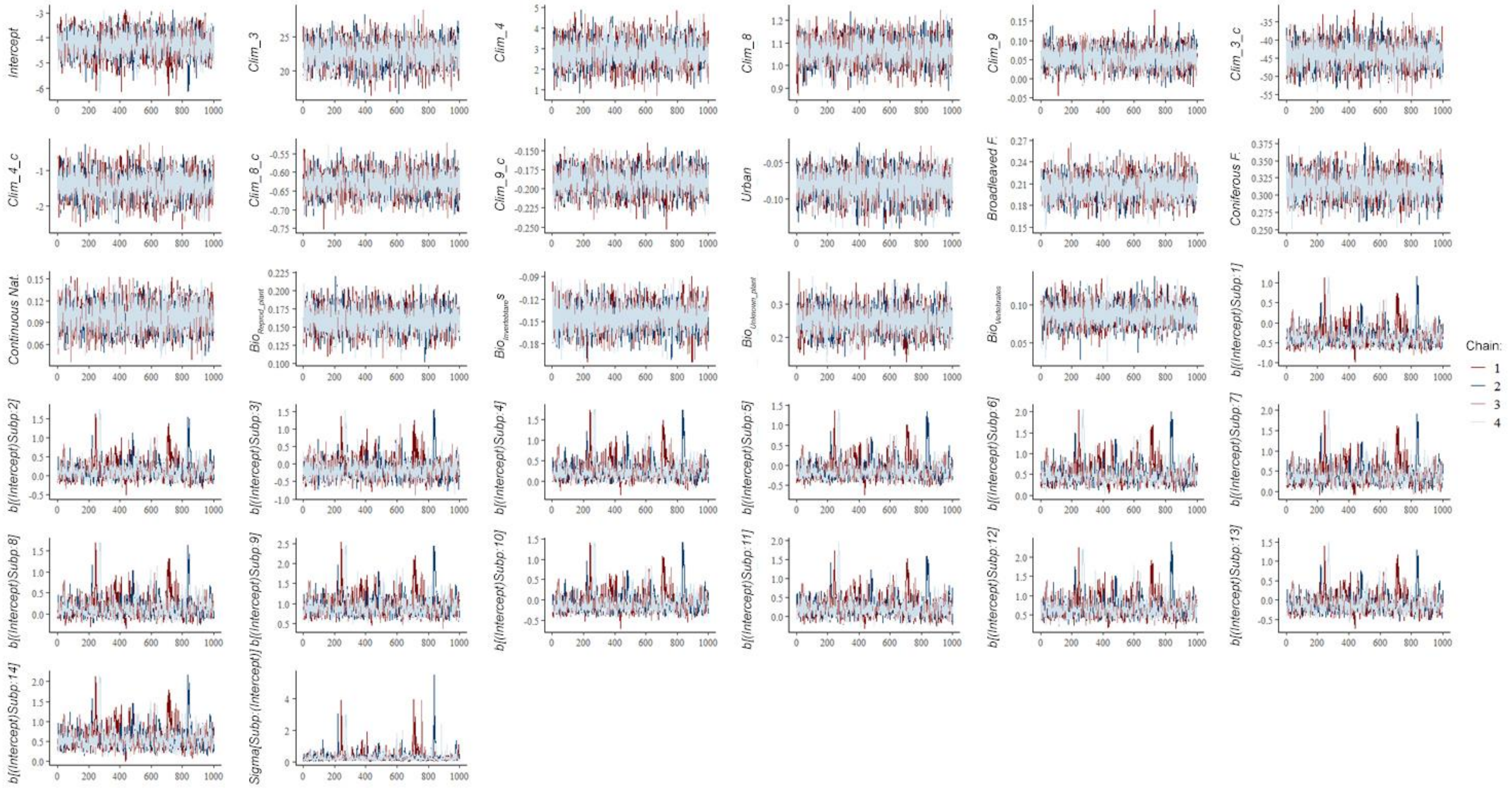

Supplementary Figure 14. Chains for the Bayesian model of brown bear habitat with abiotic and biotic factors combining data.

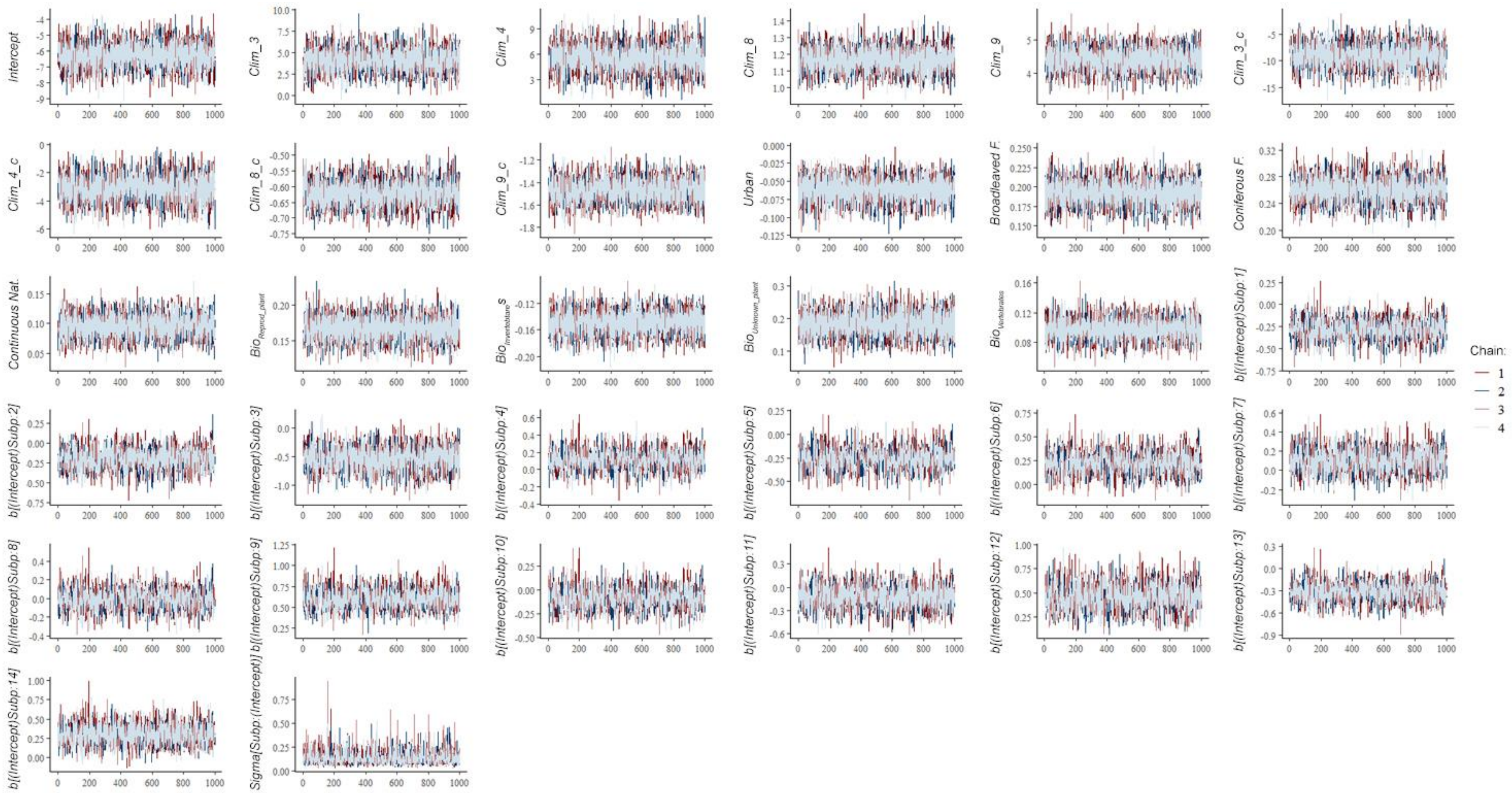

Supplementary Figure 15. Chains for the Bayesian model of brown bear habitat with abiotic and biotic factors using current data.

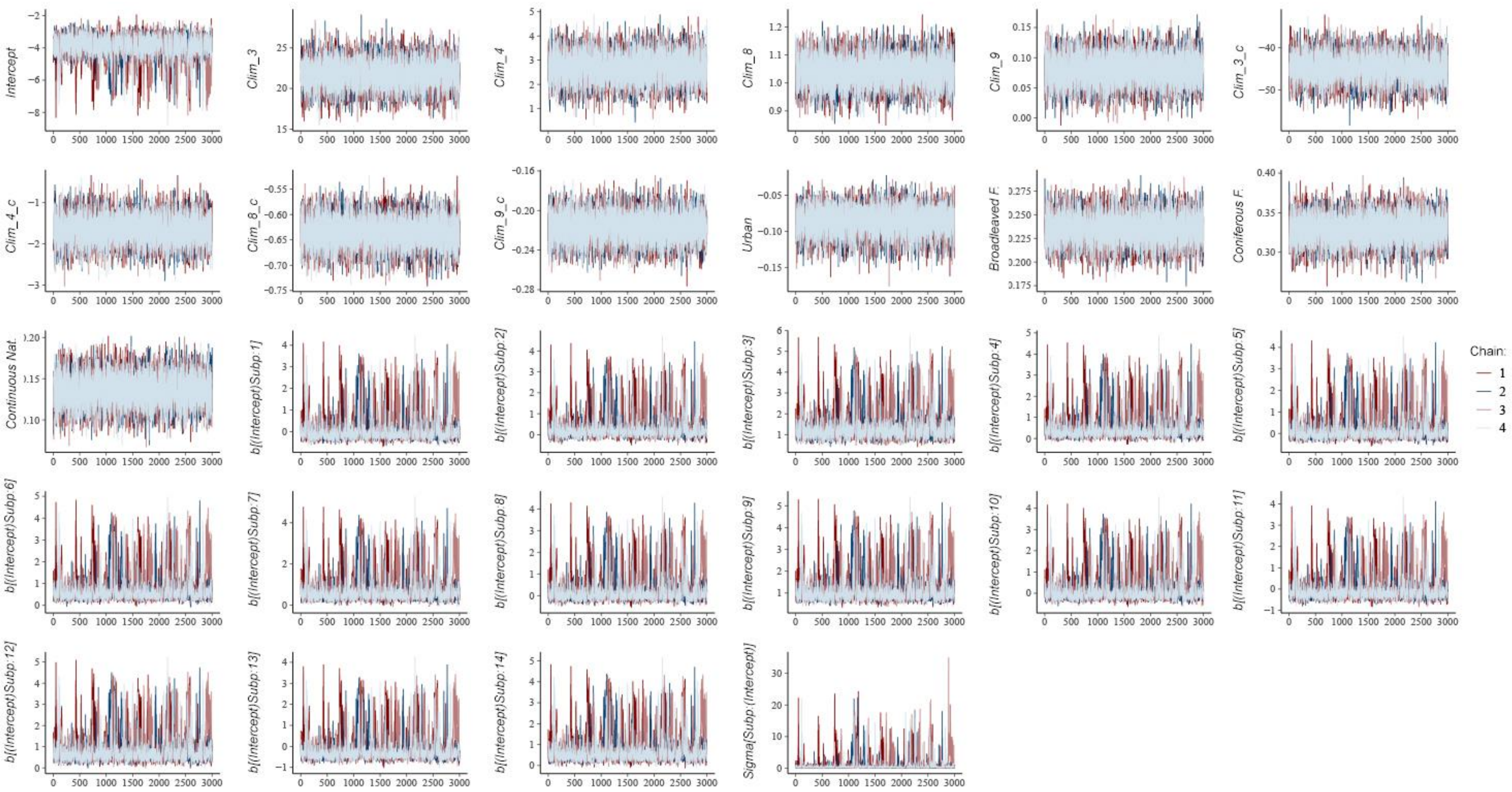

Supplementary Figure 16. Chains for the Bayesian model of brown bear habitat with abiotic factors combining data.

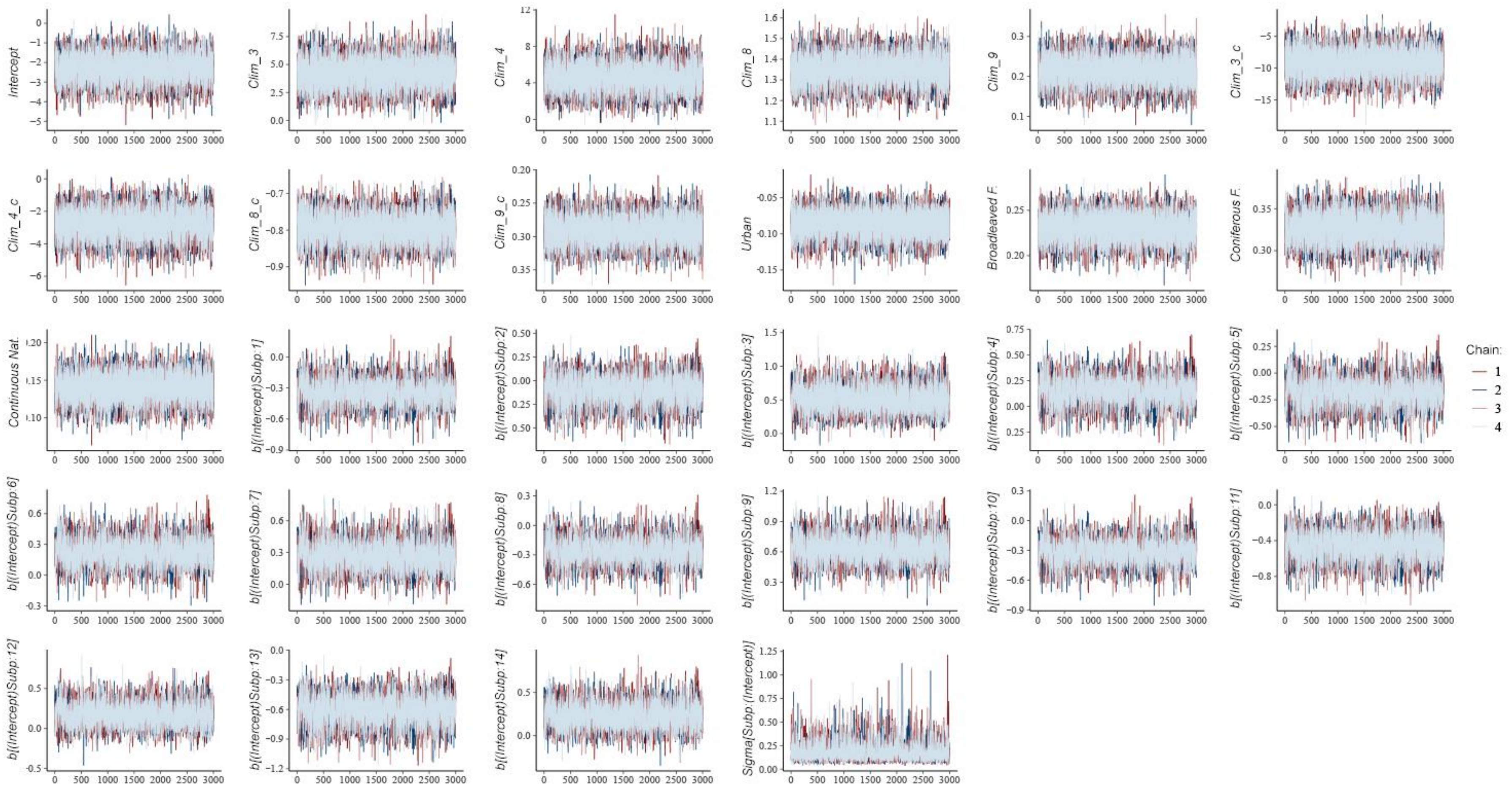

Supplementary Figure 17. Chains for the Bayesian model of brown bear habitat with abiotic factors using current data.

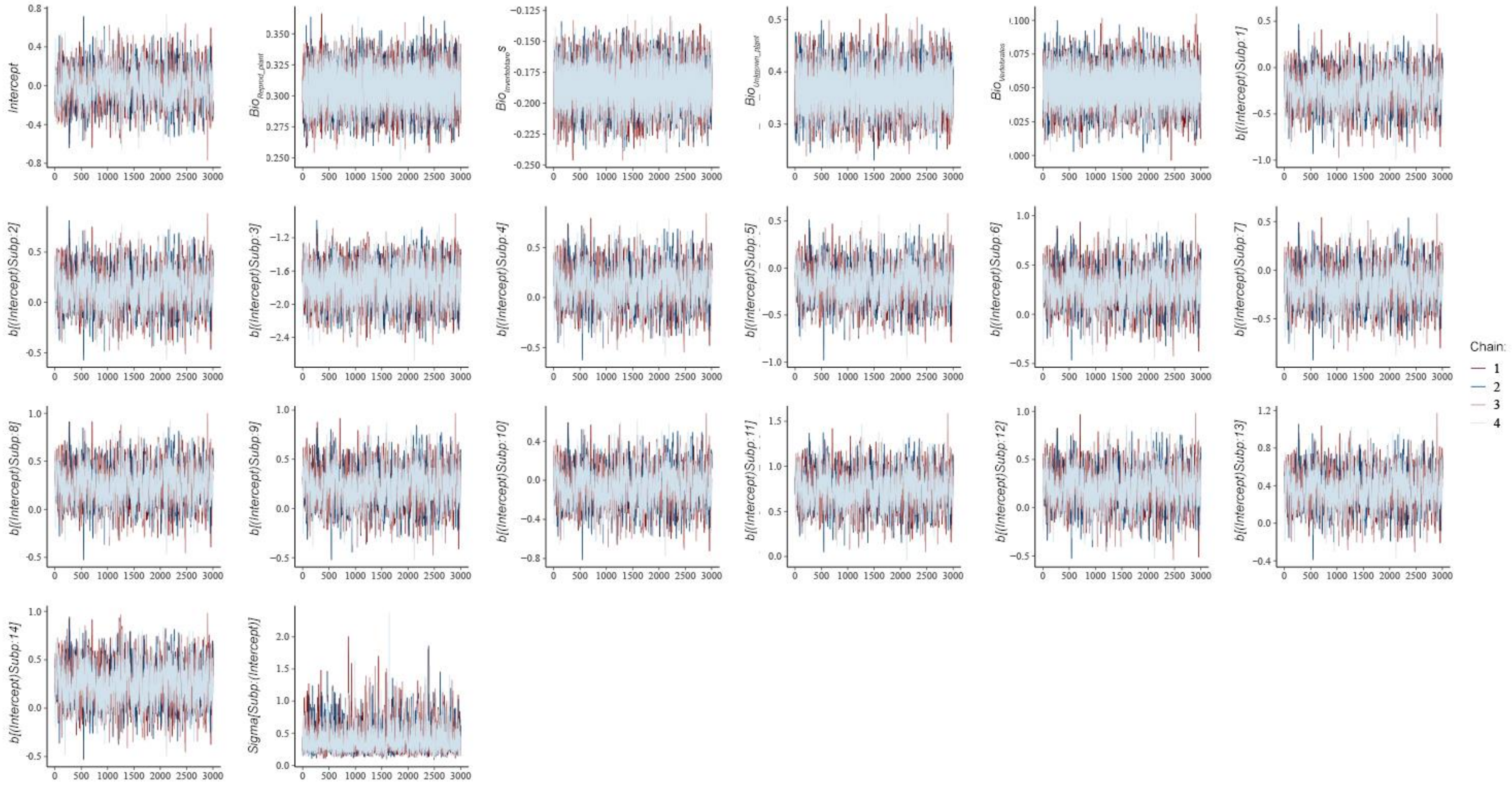

Supplementary Figure 18. Chains for the Bayesian model of brown bear habitat with biotic factors.

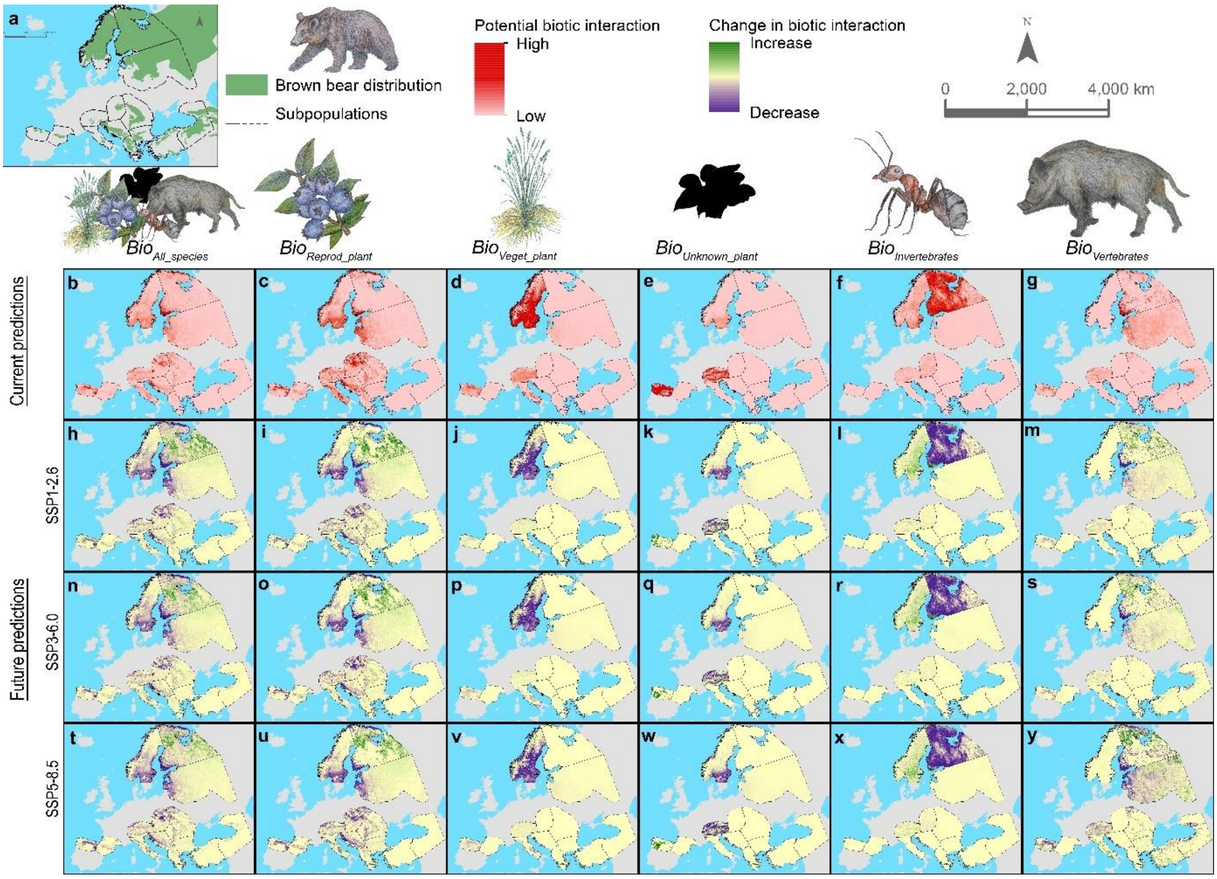

Supplementary Figure 19. Map showing the current biotic variables and the change in biotic variables for the future SSPs. **a** Current distribution of the brown bear in Europe. **b-g** Current prediction for *Biotic variables*. **h-y** Future prediction for *Biotic variables* for the three shared socioeconomic pathways considered.

REFERENCES

Albrecht J, Bartoń KA, Selva N, Sommer RS, Swenson J, Bischof R. 2017. Humans and climate change drove the Holocene decline of the brown bear. Scientific Reports.

Allouche O, Tsoar A, Kadmon R. 2006. Assessing the accuracy of species distribution models: prevalence, kappa and the true skill statistic (TSS). Journal of Applied Ecology **43**:1223-1232.

Álvares F, Domingues J. 2010. Presence histórica do urso em Portugal e testemunhos da sua relação com as comunidades rurais. AÇAFA On Line **3**.

Banašek-Richter C, Bersier LF, Ecology C-MF. 2009. Complexity in quantitative food webs. Ecology.

Bencatel J, Ferreira CC, Barbosa AM, Rosalino LM, Álvares F. 2018. Research trends and geographical distribution of mammalian carnivores in Portugal (SW Europe). PLoS One **13**:e0207866.

Bojarska K, Selva N. 2012. Spatial patterns in brown bear Ursus arctos diet: the role of geographical and environmental factors. Mammal Review **42**:120-143.

Burnham KP, Anderson DR. 2004. Multimodel inference - understanding AIC and BIC in model selection. Sociological Methods & Research **33**:261-304.

Chamberlain S, Barve V, Mcglinn D, Oldoni D, Desmet P, Geffert L, K R. 2018. rgbif: Interface to the Global Biodiversity Information Facility API. R package version 1.1.0, https://CRAN.R-project.org/package=rgbif.

Chapron G, et al. 2014. Recovery of large carnivores in Europe's modern human-dominated landscapes. Science **346**:1517-1519.

Dallas T, Decker RR, Hastings A. 2017. Species are not most abundant in the centre of their geographic range or climatic niche. Ecology letters **20**:1526-1533.

Dellink R, Chateau J, Lanzi E, Magné B. 2017. Long-term economic growth projections in the Shared Socioeconomic Pathways. Global Environmental Change **42**:200-214.

Dormann CF, Elith J, Bacher S, Buchmann C, Carl G, Carré G, Marquéz JR, Gruber B, Lafourcade B, Leitão PJ. 2013. Collinearity: a review of methods to deal with it and a simulation study evaluating their performance. Ecography **36**:27-46.

Dyderski MK, Pa S, Frelich LE, ski AM. 2018. How much does climate change threaten European forest tree species distributions? Global change biology **24**:1150-1163.

Elgmork K, Kaasa J. 1992. Food Habits and Foraging of the Brown Bear Ursus arctos in Central South Norway. Ecography **15**:101-110.

Faurby S, Araújo MB. 2018. Anthropogenic range contractions bias species climate change forecasts. Nature Climate Change **8**:252-256.

Filazzola A, Sotomayor DA, Lortie CJ. 2018. Modelling the niche space of desert annuals needs to include positive interactions. Oikos **127**:264-273.

Gelman A, Rubin DB. 1992. Inference from Iterative Simulation Using Multiple Sequences. Statistical Science **7**:457-472, 416.

Hadfield JD. 2010. MCMC Methods for Multi-Response Generalized Linear Mixed Models: The MCMCglmm R Package. Journal of Statistical Software **33**:1 - 22.

Hewitt DG, Robbins CT. 1996. Estimating Grizzly Bear Food Habits from Fecal Analysis. Wildlife Society Bulletin (1973-2006) **24**:547-550.

IPCC 2014. Climate Change 2013: The physical science basis. Contribution of Working Group I to the Fifth Assessment Report of the Intergovermental Panel on Climate Change. Cambridge University Press, Cambridge.

Karger DN, Conrad O, Böhner J, Kawohl T, Kreft H, Soria-Auza RW, Zimmermann NE, Linder HP, Kessler M. 2017. Climatologies at high resolution for the earth's land surface areas. Scientific Data **4**:170122.

Klonner G, Dullinger I, and … W-J. 2017. Will climate change increase hybridization risk between potential plant invaders and their congeners in Europe? Diversity and ….

Linnell J., V. Salvatori, Boitani L 2008. Guidelines for population level management plans for large carnivores in Europe. A Large Carnivore Initiative for Europe report prepared for the European Commission (contract 070501/2005/424162/MAR/B2).

Lorenzen ED, et al. 2011. Species-specific responses of Late Quaternary megafauna to climate and humans. Nature **479**:359-364.

McLellan BN, Proctor MF, Huber D, Michel S 2017. Ursus arctos (amended version of 2017 assessment). The IUCN Red List of Threatened Species. https://www.iucnredlist.org/species/41688/121229971.

Meinshausen M, et al. 2011. The RCP greenhouse gas concentrations and their extensions from 1765 to 2300. Climatic Change **109**:213.

Mignot J, Bony S. 2013. Presentation and analysis of the IPSL and CNRM climate models used in CMIP5. Climate Dynamics **40**:2089-2089.

Mounce R, Smith P, Brockington S. 2017. Ex situ conservation of plant diversity in the world's botanic gardens. Nature Plants **3**:795-802.

Naimi B, Hamm NAS, Groen TA, Skidmore AK, Toxopeus AG. 2014. Where is positional uncertainty a problem for species distribution modelling? Ecography **37**:191-203.

Naves J, Fernández-Gil A. 2017. A conservación do oso pardo en Galicia. Cerna **78**:43-45.

Naves J, Fernandez-Gil A, Rodriguez C, Delibes M. 2006. Brown bear food habits at the border of its range: A long-term study. Journal of Mammalogy **87**:899-908.

Parde J-M, Camarra J-J. 1992. L´ours (*Ursus arctos*, Linnaeus, 1758). Encyclopédie des carnivores de France. Société Française Pour l'Etude et la Protection des Mammifères.

Plummer M, Best N, Cowles K, K V. 2006. CODA: Convergence Diagnosis and Output Analysis for MCMC. R News **6**:7–11.

Posillico M, Meriggi A, Pagnin E, Lovari S, Russo L. 2004. A habitat model for brown bear conservation and land use planning in the central Apennines. Biological Conservation **118**:141-150.

Pritchard GT, Robbins CT. 1990. Digestive and metabolic efficiencies of grizzly and black bears. Canadian Journal of Zoology **68**:1645-1651.

Riahi K, Rao S, Krey V, Cho C, Chirkov V, Fischer G, Kindermann G, Nakicenovic N, Rafaj P. 2011. RCP 8.5—A scenario of comparatively high greenhouse gas emissions. Climatic Change **109**:33.

Roll U, et al. 2017. The global distribution of tetrapods reveals a need for targeted reptile conservation. Nature Ecology & Evolution **1**:1677-1682.

Schipper AM, et al. 2020. Projecting terrestrial biodiversity intactness with GLOBIO 4. Global Change Biology **26**:760-771.

Servheen C, Herrero S, Peyton B 1999. Bears. Status Survey and Conservation Action Plan. IUCN/SSC. IUCN, Gland, Switzerland, and Cambridge, UK.

Stenset NE, et al. 2016. Seasonal and annual variation in the diet of brown bears *Ursus arctos* in the boreal forest of southcentral Sweden. Wildlife Biology **22**:10.

Straka M, Paule L, Ionescu O, Štofík J, Adamec M. 2012. Microsatellite diversity and structure of Carpathian brown bears (Ursus arctos): consequences of human caused fragmentation. Conservation Genetics **13**:153-164.

Thuiller W, Brotons L, Araujo MB, Lavorel S. 2004. Effects of restricting environmental range of data to project current and future species distributions. Ecography **27**:165-172.

Thuiller W, Guéguen M, Renaud J, Karger DN, Zimmermann NE. 2019. Uncertainty in ensembles of global biodiversity scenarios. Nature Communications **10**:1446.

Valverde JA 2009. Anotaciones al "Libro de la Montería" del Rey Alfonso XI. Ediciones Universidad de Salamanca, Salamanca.

van Vuuren DP, et al. 2011. The representative concentration pathways: an overview. Climatic Change **109**:5.

Zizka A, et al. 2019. CoordinateCleaner: Standardized cleaning of occurrence records from biological collection databases. Methods in Ecology and Evolution **10**:744-751.
